# Immuno-inflammatory-metabolic interactions in cardiovascular diseases: a review from basic mechanisms to clinical translation

**DOI:** 10.3389/fimmu.2026.1818835

**Published:** 2026-04-15

**Authors:** Xingshun Zhu, Fengmei Zhang, Yuxin Wei, Yan Zhao, Jiawei Guo

**Affiliations:** 1 Department of Vascular and Endovascular Surgery, The First Affiliated Hospital of Yangtze University, Jingzhou, China; 2 Department of Pharmacology, School of Medicine, Yangtze University, Jingzhou, China

**Keywords:** Allostatic load, cardiovascular disease, immune inflammation, lifestyle intervention, metabolic reprogramming, thrombo-inflammation, trained immunity

## Abstract

Cardiovascular disease (CVD) remains the leading cause of mortality and disability worldwide, imposing a substantial burden on individuals, families, and healthcare systems. Despite major advances in controlling conventional risk factors (e.g., blood pressure, glycaemia, and lipids), a considerable residual risk persists, highlighting the need to elucidate additional pathogenic mechanisms and to develop more effective preventive and therapeutic strategies. Accumulating experimental and clinical evidence indicates that immune dysregulation and chronic low-grade inflammation are not merely associated with CVD but actively drive disease progression—from lesion initiation to acute thrombotic events. These processes are further shaped by metabolic status, lifestyle factors, psychosocial stress, and environmental exposures, and age-related genetic immune changes such as clonal hematopoiesis of indeterminate potential (CHIP). Atherosclerosis, the predominant pathological substrate of most CVDs, is now widely recognized as a chronic immune-inflammatory disease. Emerging concepts including immunometabolic reprogramming, trained immunity(distinguished by central and peripheral subtypes), the thrombo-inflammatory axis, and allostatic load provide an integrative framework for understanding CVD as a systemic disorder. Here, we synthesize recent advances in innate and adaptive immune mechanisms, immunometabolic dysregulation, and inflammation–thrombosis crosstalk that collectively govern plaque formation, destabilization, and clinical events. We also discuss how lifestyle-related factors (e.g., diet, fasting, physical activity, and stress) may modulate long-term cardiovascular risk through trained immunity and inflammatory pathways, and we highlight progress in immune biomarkers and anti-inflammatory interventions, and the immunometabolic effects of modern cardiometabolic drugs (GLP-1 receptor agonists, SGLT2 inhibitors). Additionally, we elaborate on the translational potential of short chain fatty acid derivatives in reversing innate immune inflammatory memory, and clarify the distinct cardiovascular toxic mechanisms of immune checkpoint inhibitors (ICIs) and chimeric antigen receptor T-cell (CAR-T) therapy in cardio-oncology. Conceptualizing CVD as a systemic immune–metabolic–inflammatory disease may facilitate improved risk stratification and inform precision prevention and treatment strategies.

## 1 Introduction

CVD research and clinical care have been centered on standard pathological disorders, such as dyslipidemia, hemodynamics and thrombosis. These treatments have focused on lipid lowering, blood pressure control, and anticoagulation. This model has resulted in a “lumen stenosis–ischemic event” model. It has been successful for many patients, but the model has become insufficient in clinical practice. A main difficulty of this model is that it does not take into account the heterogeneous progression of CVD among individuals; for example, some people develop recurring adverse cardiovascular diseases even if traditional risk factors have been known to be properly controlled, and others do not develop overt vascular disease for decades (1). Age-related genetic immune alterations represent a key yet underrecognized driver of this heterogeneity, with clonal hematopoiesis of indeterminate potential (CHIP) emerging as a major independent contributor to meta-inflammation. CHIP is characterized by age-related mutations in blood stem cells (e.g., TET2, DNMT3A) that drive the generation of highly inflammatory macrophages, accelerating atherosclerosis and heart failure progression (2, 3). It also does not consider the pathogenic influence of non-traditional risk factors such as psychological stress, environmental pollutants and chronic social deprivation. These factors are always to be viewed as major drivers of CVD development, and underscore the need for a more general pathological model which incorporates multiple biological systems (4–6).

Recent immunological and inflammatory studies have shown that persistent immune activation and low-grade chronic inflammation is key drivers of CVD at all stages – from the disease initiation and to the onset of the acute events. Atherosclerosis, once a simple lipid deposition disorder, is today a chronic inflammatory disease (7, 8). Immune-inflammatory cells (e.g., macrophages, T lymphocytes, neutrophils, dendritic cells), and inflammatory mediators (e.g., tumor necrosis factor-α (TNF-α), interleukin-6 [IL-6], interleukin-1β [IL1β] and interferon-γ [IFN-γ] are critical factors in atherogenesis as well, from early inflammation and lipid deposition through inflammatory cell infiltration and plaque formation, to late-stage plaque instability and rupture (9, 10). Acute cardiovascular events such as acute coronary syndromes (ACS) and ischemic stroke occur frequently due to inflammation-mediated plaque instability as well as thrombo-inflammatory processes (11).

In this context, inflammatory cytokines are triggers that translate chronic long-term vascular lesion into acute clinical events. Furthermore, many systemic factors (like metabolism, dietary, psychological load, environmental factors, and drug-drugs) regulate immune function by complex regulatory networks that, for each individual, alters specific long-time risk structures (12), for instance, chronic intake of a high fat, high-sugar Western diet suppresses immune cells and promotes pro-inflammatory phenotypes (13). Social stress causes vascular inflammation via neuro-endocrine-immune axis interactions, and environmental pollutants such as PM2.5 drive immune system sensitization and low-grade inflammation (6, 14, 15). These results have revolutionized the studies of CVD from single pathway analysis to multi-system integration.

**Figure 1:**
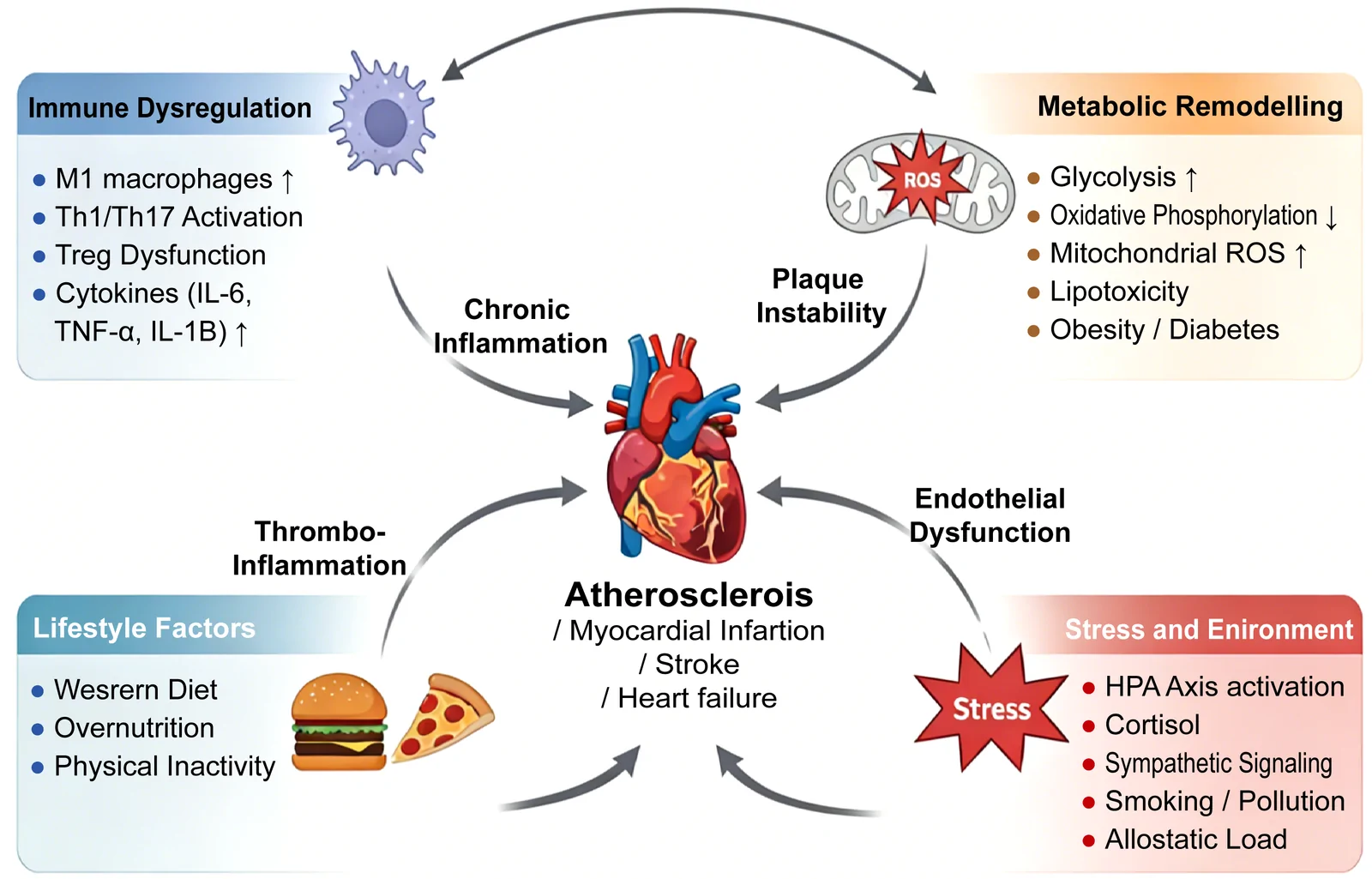
Integrated immuno-inflammatory-metabolic network driving the development of cardiovascular diseases. Legend: Immune dysregulation and metabolic remodeling form a self-reinforcing cycle that drives chronic inflammation and accelerates atherosclerosis and its complications. Immune dysregulation is characterized by increased M1 macrophage polarization, activation of Th1 and Th17 cells, impaired Treg function, and elevated pro-inflammatory cytokines. Metabolic remodeling involves enhanced glycolysis, reduced oxidative phosphorylation, mitochondrial dysfunction with increased ROS, lipotoxicity, and metabolic disorders such as obesity and diabetes. Lifestyle factors and stress-related environmental exposures, including hypothalamic–pituitary–adrenal (HPA) axis activation, cortisol release, sympathetic signaling, smoking, and pollution, exacerbate endothelial dysfunction, plaque instability, and thrombo-inflammation, ultimately promoting cardiovascular events. HPA axis, hypothalamic–pituitary–adrenal axis; IL, interleukin; ROS, reactive oxygen species; Th1, T helper 1; Th17, T helper 17; TNF-α, tumor necrosis factor-alpha; Treg, regulatory T cells.

Against this backdrop, we turn our current focus towards systems biology where we build upon the knowledge about immunology, metabolism, neuroscience and environmental health to understand the immune-inflammatory effects of CVD and clinical applications, as is shown in **Figure 1**.

## 2 Fundamental immune and inflammatory mechanisms in cardiovascular disease

The immune-inflammatory response to CVD is a very complex process which arises not due to the abnormality of a single cell or molecule but due to several immune cells, inflammatory mediators and vascular wall structural cells in specific metabolic and mechanical microenvironment. It takes place at a variety of levels, in terms of the interplay of innate and adaptive immunity, inflammation/thrombosis-coagulation and the interrelation between immunity and metabolism (16, 17). Notably, emerging concepts such as trained immunity and innate immune memory, as well as age-related genetic immune changes like Clonal Hematopoiesis of Indeterminate Potential (CHIP), are critical to understanding the persistence and individual variability of CVD-related inflammation, and are integrated into the following discussion (18, 19, 90). This chapter addresses key mechanisms of immune-inflammatory response to CVD from innate-adaptive immune imbalance, inflammation-thrombosis-coagulation axis, and immune-metabolic interactions, and further incorporates emerging research progress and clinical translational implications.

### 2.1 Synergistic imbalance between innate and adaptive immunity

The innate immune system acts as an initial defense against external pathogens and external damage signals. It initiates and maintains cardiovascular inflammatory responses and mediates chronic inflammation. Unlike the adaptive immune system, it responds quickly without prior sensitization, and is one of the early targets of vascular inflammation (20). A core emerging feature of innate immunity in CVD is innate immune memory, a subtype of trained immunity that is distinct from classical beta-glucan-induced trained immunity and is driven by sustained low-grade inflammatory signals (21, 22).

Monocytes are key effectors of the innate immune system. In blood, they patrol for signs of vascular damage or inflammation. When exposed to inflammatory signals (oxLDL, DAMP, or PAMP) the monocytes are recruited to the target of the damage, which is mediated by chemokine signals: monocyte chemoattractant protein-1 (MCP-1) (23). Once they enter the blood wall, monocytes reach the intima and differentiate into macrophages with local microenvironmental cues (cytokines, growth factors, metabolic intermediates etc.).CHIP, an age-related genetic alteration in blood stem cells (e.g., TET2 and DNMT3A mutations), drives the differentiation of monocytes into highly inflammatory macrophages, accelerating atherosclerosis and heart failure and contributing to individual differences in CVD progression (2, 3, 24). Macrophages have unique phenotypic plasticity to adapt to the local environment. Depending on function, macrophages can be grouped into two major groups: classically activated macrophage (M1 type) and alternatively activated (reparative) macrophage (M2 type), each of which regulates inflammation and tissue repair within the vascular wall (25). M1 macrophages are activated by pro-inflammatory signals (IFN-γ, LPS, TNF-alpha) First, they secrete large quantities of pro-inflammatory cytokines (TNF-α, IL-6, IL1β, IL12 and CXCL8) which induce recruitment and activation of other inflammatory cells (e.g. neutrophils and T lymphocytes) at the lesion site. M1 macrophages secrete matrix metalloproteinases (MMPs), which degrade the extracellular matrix, reactive oxygen species (ROS) that produce oxidative stress (endothelial dysfunction, vascular smooth muscle cell (VSMC) migration and proliferation and atherosclerotic plaques) (26). M2 macrophages are reparative, secreting anti-inflammatory cytokines IL-4, IL-13 and transforming growth factor-β (TGF-β) that suppress pro-inflammatory responses and promote tissue repair. M2 macrophages also pass through a scavenger receptor-mediated phagocytosis of oxLDL called “foam cell formation”, while M2 foam cells are less pro-inflammatory, and might promote plaque stability. M2 macrophages also promote collagen synthesis and extracellular matrix remodeling that helps improving the plaque fibrous cap (27).

In normal physiology, M1/M2 balance ensures low inflammation response to injury which is resolved by tissue repair. In CVD however, the balance is broken. It changes due to different factors (oxLDL, chronic hyperglycemia and pro-inflammatory cytokines) which shift the polarization towards M1 phenotype leading to low inflammation resolution and lower tissue repair conditions (28). This dominant M1 leads to a chronic inflammatory microenvironment on the vascular wall driving atherosclerotic plaques formation and progress. For example, in advanced atherosclerosis plaques, M1 macrophages accumulate in necrotic core, and secrete pro- inflammatory cytokines and MMPs that weaken the fibrous cap and lead to plaque rupture (29). This persistent M1 polarization is further reinforced by innate immune memory: diverse innate signals such as free cholesterol or oxLDL induce low-grade inflammatory memory in innate immune cells not via signal type, but via sustained signal dosage and duration, leading to irreversible chronic inflammation (21, 30). *In vivo* studies confirm this principle: mice with prolonged high-fat diet (HFD) feeding retain a low-grade inflammatory state even after HFD withdrawal, as innate immunity acts as a biological “timer”—sustained challenges trigger an irreversible low-grade inflammatory state that persists long-term (31, 32).

The adaptive immune system, while slower to respond, plays an important role in amplifying and accurately controlling inflammation in CVD. Antigens generated by APCs (DCs and macrophages) are prepared and present from the atherosclerotic microenvironment (oxLDL, heat-shock proteins and necrotic-cell debris) to naive T lymphocytes. This antigen presentation and co-stimulation signal activates a specific adaptive immune response (33). Several clinical and experimental studies have shown the significant enrichment of proinflammatory T cells (particularly Th1 and Th17) in atherosclerotic plaques. Th1 cells are activated by DC IL-12 and secrete IFN-γ, a pro-inflammatory cytokine that promotes vascular inflammation. IFNγ activates macrophages, which are enriched with pro-inflammatory cytokines and ROS, and inhibits VSMC collagen formation, which destroys the plaque fibrous cap. It is also beneficial to expression of adhesion molecules on endothelial cells for the recruitment of other inflammatory cells to lesion site (34). Th17 cells are triggered by TGF-β and IL-6, secrete IL-17A, IL- 17F and IL22. IL-17A is effective for CVD by promoting neutrophils to the vascular wall, stimulating endothelial cells and VSMCs, and promotes pro-inflammatory cytokines and chemokines. Th17 cell depletion or IL17 neutralization helps shrink atherosclerotic lesion size and plaque stability in experiments, and the Th17 cells is a pathogen in CVD (35, 36). Whereas pro-inflammatory T cells are involved in adaptive immune system, regulatory T cells (Treg cells) express or secrete anti-inflammatory cytokines such as IL-10 and TGF-β and directly disrupt pro-inflammatory T cells and macrophages and suppress abnormal inflammation. Treg cells also promote M2 polarization and repair of damaged tissues, which contribute to vascular homeostasis (37). A reduced Treg cell number or function is crucial for chronic vascular inflammation. Several processes contribute to Treg cells dysfunction in CVD, including oxidative stress, pro-inflammatory cytokines suppression and epigenetic changes. oxLDL may lead Treg to die and interfere with their suppressive function. IFN-γ and IL-6 can decrease Foxp3 expression and turn Tregs into pro-inflammatory effector cells (Treg cell instability). The reduced Treg cell mediated immune tolerance can also allow pro-inflammation immune responses to remain undetected, which are known as Treg cell dysfunction and atherosclerosis (38, 39).

The imbalance between innate and adaptive immunity (macrophage polarization dysregulation, pro-inflammatory T cell activation, Treg cell dysregulation, and innate immune memory/trained immunity dysregulation) leads to a self-perpetuating inflammatory process in the vascular wall. This immunological dysregulation is the source of CVD initiation and growth. It is an important area of use for therapeutics (40).

### 2.2 Formation and amplification of the inflammation-thrombosis-coagulation axis

Inflammation and thrombosis in CVD are both in a bi-directional manner, forming a dynamic and self-amplifying inflammation-thrombotic-coagulation axis which connects chronic vascular inflammation to acute cardiovascular events, playing a key role in disease progression and acute adverse outcomes (41). Inflammation promotes thrombinogenesis and thrombosis promotes inflammation, a vicious cycle that induces vascular damage and a high probability of acute events. The inflammation-thrombosis-coagulation axis is activated by multiple mechanisms in the inflammatory microenvironment of the vascular wall. Active immune cells (monocytes/macrophages/neutrophils) and endothelial cells release pro-inflammatory cytokines and pro-coagulants in a direct or indirect manner that, in combination with the extrinsic (primary pathway) and intrinsic coagulation pathways, inhibit the fibrinolytic system, leading to hypercoagulability and thrombus formation (42). Other than the coagulation cascade, pro-inflammatory cytokines block the fibrinolytic system and promote thrombus stability, downregulate the expression of tissue-type plasminogen activator (tPA) (the main activator of plasmin that degrades fibrin), and upregulate plasminogen activator inhibitor-1 (PAI-1), a strong inhibitor of tPA, which inhibits fibrinolysis upon plasmin system disruption and allows thrombi to develop (43).

Platelets, well known to be responsible for hemostasis, are hubs in the inflammation-thrombosis-coagulation axis that integrate inflammatory and thrombotic signals. Platelets express diverse surface receptors, such as adhesion molecules, cytokine receptors and pattern recognition receptors to sense and respond to inflammatory signals (44). When exposed to pro-inflammatory cytokines or damaged blood vessels, platelets are activated, undergoing shape change, degranulation and upregulation of adhesion molecules. Platelets also interact with other cells in the vascular microenvironment: activated platelets express P-selectin, which binds P-selectin glycoprotein ligand-1 (PSGL-1) on neutrophils and monocytes, forming platelet-leukocyte complexes. These complexes enhance local inflammation by promoting leukocyte activation and proinflammatory activity (45). Activated platelets release inflammatory mediators from their granules such as PAF, TXA2 and chemokines such as CXCL4 (platelet factor 4), CXCL7 (neutrophil activating peptide 2). PAF and TXA2 are pro-inflammatory and pro-thrombotic mediators that induce leukocyte recruitment, endothelial activation and platelet aggregation. CXCL4 and CXCL7 recruit monocytes and neutrophils to the lesion site, exacerbating inflammation, and can induce the release of growth factors such as platelet derived growth factor (PDGF) and vascular endothelial growth factor(VEGF) that promote VSMC proliferation and migration, leading to atherosclerotic plaque progression (46). Besides promoting inflammation, activated platelets act directly at the vascular injury site, binding to collagen fibers at the wound site via glycoprotein Ib/IX/V and glycoprotein VI receptors, forming a platelet plug. Activated platelets also express phosphatidylserine on their surface and act as a pro-coagulant phospholipid scaffold that enhances coagulation factor assembly and activation, facilitating thrombin generation and fibrin clot formation (47).

Neutrophils are the most abundant leukocytes in the circulation and are responsible for the activation of the inflammation-thrombosis-coagulation axis during acute inflammation or plaque rupture (48). Neutrophil extracellular traps (NETs) are composed of chromatin, histones, granular proteins (Myeloperoxidase, elastase, cathepsin G) when activated by pro-inflammatory cytokines or platelet-derived mediators. NETs cause thrombosis with several complementary mechanisms: (1) Chromatin backbone in NETs are a physical scaffold which traps platelets, red blood cells and coagulation factors concentrating them at their site of injury and making thrombus formation faster; (2) histones and granular proteins are pro-coagulant and active and target platelets and coagulation and inhibit fibrinolytic enzymes; histones are able to activate platelets by their production and enhance thrombin and myeloperoxidase inhibits fibrinogen, and makes it less susceptible to plasmin degradation. (3) NETs degrade vascular endothelial cells by cytotoxic histones/granular enzymes that break the endothelial barrier and promote platelet adhesion and aggregation (49, 50). Besides their pro-thrombotic effect, NETs enhance inflammation via active complement system and recruit new inflammatory cells. They also proliferate pro-inflammatory cytokines and adhesion molecules on endothelial cells, providing a feedforward loop that drives inflammation-thrombosis-coagulation (51). NET markers (such as cell free DNA-histone complexes, myeloperoxidase-DNA complexes) have been observed in ACS, deep vein thrombosis and stroke patients, indicating clinical relevance of NETs for acute cardiovascular diseases (52).

Activating chemicals of the coagulation system (thrombin, fibrin, FXa, FVIIa) also produce inflammation and complete the crosstalk between coagulating and inflammation. These chemicals bind to protease-activated receptors (PARs) on the immune cells and endothelial cells, including PAR-1 and PAR-2, and initiate intracellular signaling pathways promoting inflammation (53). Thrombin is also an important inflammatory system. A binding to PAR-1 on the endothelial cells results in expression of adhesion molecules (ICAM-1, VCAM- 1) and pro-inflammatory cytokines (IL-6, MCP-1) leading to leukocyte recruitment and activation. Thrombin activates monocytes and macrophages, in which they secrete pro-inflammatory cytokines and ROS. FXa and FVIIa bind to PARs on the immune cells and the endothelial cells to activate inflammatory signals and activate the inflammatory response (54). This coagulation-induced inflammation triggers an increasing inflammation and inflammation promotes coagulation, which further drives the inflammation-thrombosis-coagulation axis. Activation of the inflammation-thrombosis-coagulation axis plays a major role in several CVDs. For ACS plaque rupture exposes the lipid core and collagen fibers to the circulation, bringing inflammation and coagulation rapidly. Due to an activation of the axis platelet thrombi and fibrin clots form quickly and grow outside the coronary artery, causing acute myocardial infarction (55). For ischemia-reperfusion injury, tissue damage from ischemia triggers intense inflammation, activates the axis and forms micro-strombi in the microvasculature that aggravate tissue hypoxia and reperfusion injury, causing slow organ dysfunction. For SIRS and sepsis, increasing inflammatory activation significantly causes systemic coagulation and disseminated intravascular coagulating (DIC) with multiple-organ dysfunction, including severe cardiovascular disruption (56).

**Figure 2:**
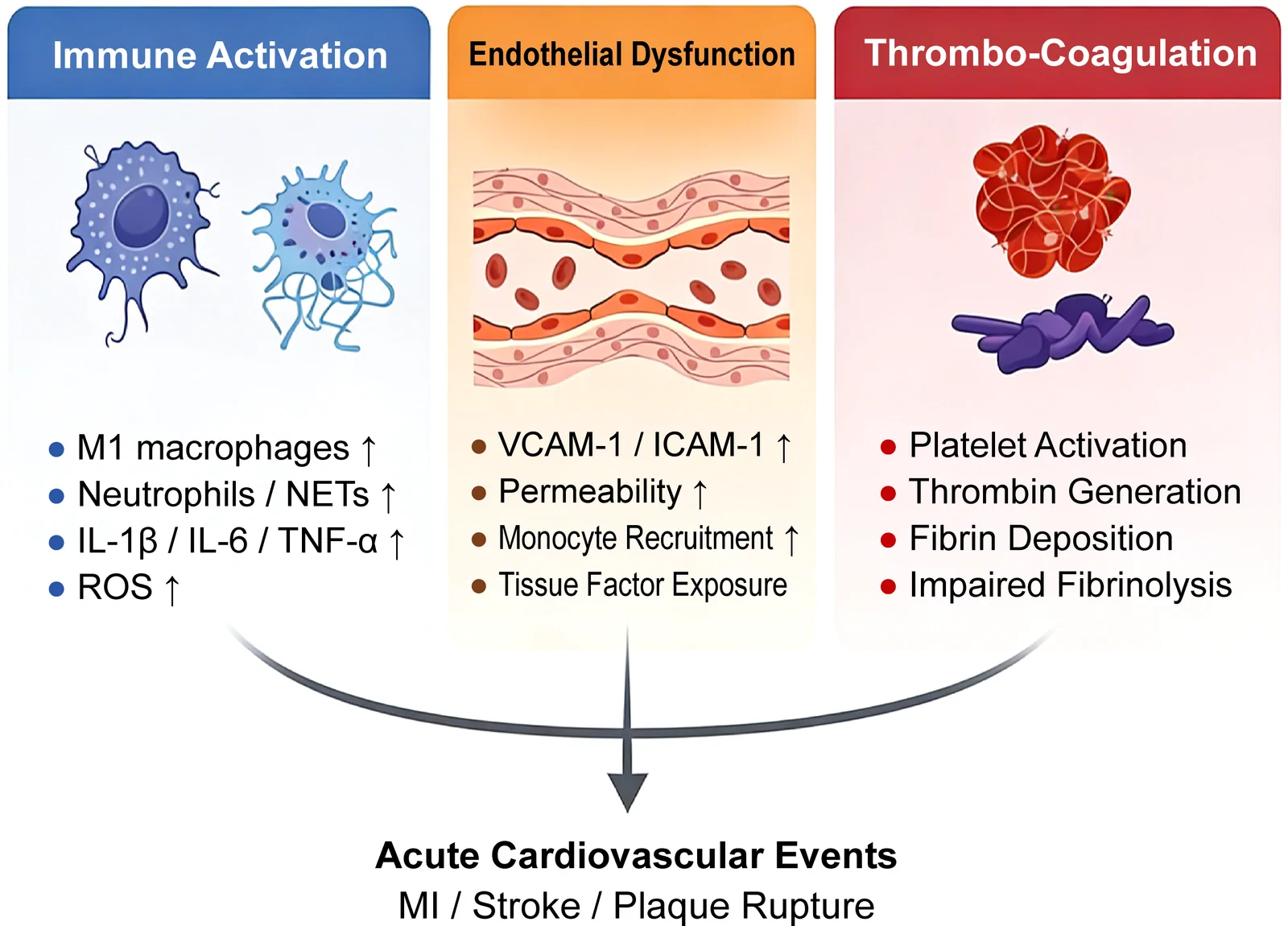
The inflammation–thrombosis–coagulation axis linking immune activation to acute cardiovascular events. Legend: Immune activation, endothelial dysfunction, and thrombo-coagulation act synergistically to precipitate acute cardiovascular events. Immune activation involves M1 macrophage polarization, neutrophil activation and neutrophil extracellular trap (NET) formation, increased pro-inflammatory cytokines, and elevated ROS. Endothelial dysfunction is characterized by upregulation of adhesion molecules (VCAM-1 and ICAM-1), increased vascular permeability, enhanced monocyte recruitment, and tissue factor exposure. Thrombo-coagulation is marked by platelet activation, thrombin generation, fibrin deposition, and impaired fibrinolysis. These interconnected processes promote plaque rupture and lead to myocardial infarction (MI) and stroke. ICAM-1, intercellular adhesion molecule 1; IL, interleukin; MI, myocardial infarction; NETs, neutrophil extracellular traps; ROS, reactive oxygen species; TNF-α, tumor necrosis factor-alpha; VCAM-1, vascular cell adhesion molecule 1.

Overall, inflammation-thrombosis-coagulation axis plays a fundamental role in the interplay of chronic vascular inflammation with acute cardiovascular events, as is shown in **Figure 2**.

### 2.3 Immune-metabolic interactions and the persistence of inflammation

The initiation, development and maintenance of immune responses depends upon cellular metabolic conditions. Active cells in activation, proliferation, differentiation and functional activity require adequate energy and biological ingredients from cellular metabolic processes. However, changes in metabolic conditions can also influence immune cell function and intensity of inflammatory response, resulting in a dual relationship between metabolism and immunity (immune-metabolic coupling) (57, 58). In CVD, immune-metabolic dysregulation has an important role in maintaining chronic inflammation, which is the basic metabolic substrate for chronic heart disease (59).

Most immune cells use oxidative phosphorylation (OXPHOS) as their main source of energy. OXPHOS is a powerful metabolic process that catalyzes the production of large amounts of ATP through glucose, fatty acids or amino acids in the mitochondria and has little production of metabolic byproducts. This allows immune cells to stay alive and to maintain a homeostatic state (60). When activated by inflammatory processes (e.g., oxLDL, DAMPs or pro-inflammatory cytokines), immune cells are effectively reprogrammed from OXPHOS to glycolysis.

Glycolysis converts glucose to pyruvate, and produces a small amount of ATP in cytoplasm without oxygen. While glycolysis is less efficient as a pathway than OXPHOS, glycolysis provides fast energy that is required in order to meet the high energy needs of immune cell activation, proliferation and effector function. Glycolysis also provides key biosynthetic precursors—including ribose-5-phosphate (i.e. nucleic acid synthesis), glycerol-3-phosphate (i.e. lipid synthesis) and pyruvate (i.e. amino acid synthesis)—which are needed for immune cell proliferation and production of inflammatory mediators (61).

Macrophages are a common type of immune cells which undergo metabolic reprogramming after activation. In activation of pro-inflammatory signals like LPS or IFN-γ, macrophages activate the expression of key glycolytic enzymes such as hexokinase 2 (HK2), phosphofructokinase 1 (PFK1) and lactate dehydrogenase A (LDHA) that lead to higher glycolytic flux. This increases pro-inflammatory cytokines (TNF-α, IL-1β, IL-6) and ROS in the case of M1 macrophage which, in turn, solidifies the pro-inflammatory phenotype of M1 macrophages (62). In contrast, M2 Macrophages activated by IL-4 or IL-13 rely predominantly on OXPHOS and fatty acid oxidation (FAO) which also support their anti-inflammatory and reparative function (63). CHIP-associated macrophages exhibit aberrant metabolic reprogramming, with enhanced glycolysis and impaired FAO, further reinforcing their pro-inflammatory phenotype (2, 64).

T cells also become reprogrammed during activation and differentiation. Naive T cells depend on OXPHOS to obtain energy. Upon activation by antigen and co-stimulatory signals, T cells switch to glycolysis to stimulate their rapid growth and differentiation into effector T cells (Th1, Th17), which sustain glycolytic metabolism for the purpose of pro-inflammatory function. Treg cells and memory T cells rely more on OXPHOS and FAO for their long-term survival and functioning (65, 66).

Note that metabolic reprogramming is not simply an adaptation to energy demand, it is also an active regulator of the cell function. Metabolic intermediates produced during glycolysis or other metabolic processes act as signaling molecules. The gene expression of inflammation-associated genes may be regulated through epigenetic changes or transcription factors (67). For example, pyruvate, which is an intermediate of glycolysis, may be transferred to the nucleus, where it regulates histone acetylation by inhibiting histone deacetylases (HDAC) and modulates pro-inflammatory gene expression. Succinate, an intermediate of the TCA cycle, is produced in the macrophages during activation, and suppresses the expression of a key pro-inflammatory factor-hypoxia-inducible factor-1α (HIF-1α). HIF-1α promotes expression of pro-inflammatory signaling molecules such as IL-1β and glycolytic enzymes, which enhances the pro-inflammatory phenotype. Itaconate is another TCA-cycle intermediate, which can be produced in macrophage upon LPS activation and inhibits the activity of NF-κB, which plays a major role in pro-inflammatory responses (68, 69). This epigenetic regulation by metabolic intermediates is a core mechanism of innate immune memory, as sustained metabolic reprogramming induces stable epigenetic changes in innate immune cells (70).

Metabolic disorders - a hallmark of most CVDs (e.g., obesity, type 2 diabetes, dyslipidemia) can disrupt immune-metabolic crosstalk, resulting in persistent immune activation and chronic inflammation (71). Lipid disorders play a key role in this process. Excessive lipids (i.e., oxLDL and saturated fatty acids) act as DAMPs, activating the innate immune cells via pattern recognition receptors (e.g., toll-like receptor 4 (TLR4) and scavenger receptors). This triggers proinflammatory signaling pathways, which induces the release of proinflammatory cytokines and M1 macrophage polarization (72). Furthermore, abnormal lipid accumulation (lipotoxicity) affects cell metabolism and function: high cholesterol accumulation in macrophages yields foam cells, a key cell in atherosclerotic plaques. Foam cells lead to reduced cholesterol efflux, increased ROS production and elevated levels of pro-inflammatory cytokines, which promotes plaque development. Energy-rich fatty acids may induce ER stress and mitochondrial dysfunction in immune cells, which can contribute to pro-inflammatory responses (73).

Mitochondrial malfunction is another link between metabolic dysfunction and immune-inflammatory response. Mitochondria are the core organelles for OXPHOS and are also the main sources of ROS production. When exposed to adverse metabolic conditions (hyperglycemia, hyperlipidemia or obesity), mitochondrial structure and function are impaired and ROS production is increased. ROS damage DNA, proteins and lipids, induce oxidative stress and activate pro-inflammatory pathways (NF-κB and MAPK). ROS also act as second messengers to regulate inflammatory transcription factors and pro-inflammatory cytokine production (74). Mitochondrial damage also causes the release of mitochondrial DNA and mitochondrial DAMPs (mito-DAMPs) into the cytoplasm, which are recognized by pattern recognition receptors (cGAS-STING) and further activate innate immunity and inflammation (75). Vascular endothelial cells, which are essential components of the vascular wall, also contribute to immune-metabolic crosstalk and inflammation amplification. Endothelial cells are metabolically active, and their energy is derived from OXPHOS and glycolysis. When exposed to hyperglycemia or hyperlipidemia, their metabolic system is dysregulated. Glycolysis is upregulated, and OXPHOS is suppressed, which causes energy imbalance and accumulation of metabolic intermediates such as lactate. This process leads to decreased endothelial barrier function, increased vascular permeability and enables circulating immune cells and inflammatory mediators to penetrate the endothelial wall (76). Metabolically impaired endothelial cells secrete more pro-inflammatory cytokines (IL-6, MCP-1) and adhesion molecules (ICAM-1, VCAM-1), which stimulate leukocyte recruitment and activation, and exacerbate local inflammation (77).

Through these multiple regulatory processes, immune-metabolism drives a physiological, transient inflammatory response into a long-lasting pathological state. This chronic inflammation leads to the development of chronic CVDs, and targeting immune-metabolic crosstalk is key for the prevention and treatment of cardiovascular diseases (78). Additionally, translational studies have identified novel therapeutic strategies for reversing innate immune memory: short chain fatty acid (SCFA) derivatives such as 4-PBA robustly rejuvenate innate monocytes and erase low-grade inflammatory memory, representing a promising translational approach for treating chronic CVD-related inflammation (79, 80).

### 2.4 Key translational and therapeutic implications

Building on the above mechanisms, the therapeutic landscape for CVD is expanded to include emerging targets based on recent research. (1) Targeting innate immune memory/trained immunity: SCFA derivatives (4-PBA) to reverse inflammatory memory in innate monocytes; epigenetic modulators to target central/peripheral trained immunity (80, 81). (2) CHIP-related interventions: Targeting TET2/DNMT3A mutations to inhibit the differentiation of pro-inflammatory macrophages, reducing individualized CVD progression risk (2, 3, 82). (3) Cardiometabolic drug repurposing: GLP-1R agonists (Semaglutide) and SGLT2 inhibitors as anti-inflammatory agents for CVD, independent of metabolic control (83, 84). (4) Cardio-oncology toxicity management: Targeting adaptive immune overactivation for ICI-related cardiovascular damage (85, 174); inhibiting innate immune cell toxicity for CAR-T-related cardiac dysfunction (86, 87). (5) Classical anti-inflammatory strategies: Continued use of IL-1β blockade, colchicine, and NET inhibitors, combined with novel innate immune memory modulators for synergistic effects (88, 89).

## 3 Trained immunity and lifestyle factors

As the development in immunological studies progressed we have gained an insight into the innate immune system. The view that innate immune cells do not remember has been challenged by the discovery of “trained immunity”, which is that innate cells responding to pathogens or other stimuli exhibit a non-specific long-term response (90). This concept has been further refined by emerging research, which reveals that innate immune memory is not primarily determined by the nature of stimulatory signals, but by their dosage and duration. Diverse innate signals (e.g., free cholesterol, oxLDL) can induce low-grade inflammatory memory when sustained in the systemic environment for an extended period—this is distinct from the original trained immunity concept, where innate immune cells mount an amplified response after initial priming by specific ligands such as beta-glucan (21, 91). In the CVD context, this “sustained signal-induced” innate immune memory is far more clinically relevant, as chronic low-level pro-inflammatory stimuli are ubiquitous in CVD pathogenesis. Additionally, trained immunity is now classified into central and peripheral subtypes based on its cellular origin: central trained immunity occurs in bone marrow stem cells, shaping the developmental trajectory of innate immune cells, while peripheral trained immunity is driven by epigenetic and metabolic reprogramming of tissue-resident macrophages (92, 93). This is a new theoretical approach to understanding how lifestyle factors can impact long-term health through immune pathways. Diet, fasting, and exercise can induce trained immunity by changing the metabolic state and epigenetic changes of immune cells to control immune inflammation and risk of cardiovascular diseases over time (94). In this chapter, we focus on three lifestyle factors (diet, fasting and physical exercise) to elaborate on their regulatory role in immune function and CVD risk and their relative importance, and highlight translational interventions that reverse inflammatory innate immune memory.

### 3.1 Diet-induced trained immunity and chronic inflammation

Dietary patterns play important exogenous roles in the body’s immune-metabolic state and inflammation level and are linked to high CVD risk. Large epidemiological and experimental evidence suggests that long-term consumption of high fat and high sugar Western diet is associated with high CV risk (95). Th e effects of this eating pattern not only have an impact on the cardiovascular system, leading to dyslipidemia, obesity and insulin resistance, but also induce sustained signal-dependent trained immunity that contributes to long-term chronic inflammation that adversely affects cardiovascular health (13, 96).

Western diet with saturated fats, trans fats, added sugars and refined carbohydrates has been found to generate a persistent pro-inflammatory phenotype of monocytes and macrophages by epigenetic remodeling and metabolic pathways, called “diet-induced trained immunity.” Even after the initial dietary stimulus is removed, the immune cells maintain an enhanced inflammatory response when exposed to other stimuli such as pathogens, DAMPs or environmental pollutants (97). *In vivo* studies have validated this memory principle: mice fed a prolonged high-fat diet retain a low-grade inflammatory state even after withdrawal of the high-fat diet, demonstrating that innate immunity acts as a biological “timer”—once pro-inflammatory challenges persist long enough, the system enters an irreversibly sustained low-grade inflammatory state (31, 32). This memory effect of the innate immune system is responsible for the persistence of chronic inflammation and long-term risk of CVD.

The diet-induced trained immunity involves multiple pathways, especially epigenetic modifications and metabolic reprogramming. Epigenetic modifications (e.g., histone acetylation, methylation and chromatin accessibility) play a role in the long term effects of diet on immune cell function. If excess nutrients (i.e., high fat and high sugar) are consumed, histone modifications in immune cells (i.e., monocytes and macrophages) are altered. For example, high glucose increases the histone methylation in pro-inflammatory genes (TNF-α, IL-6, and IL-1β), thereby increasing their transcriptional potential (98). In contrast, saturated fatty acids are able to induce histone methylation at particular sites, promoting the expression of pro-inflammatory genes. These epigenetic changes remain stable and heritable by cell division, which enables immune cells to maintain a pro-inflammatory phenotype long after the initial diet stimulus is removed (99).

Besides epigenetic modifications, the Western diet promotes metabolic remodeling of immune cells, amplifying the trained immune phenotype. High-sugar intake increases blood glucose, promotes the insulin-PI3K-AKT signaling, which regulates the expression of important glycolysis enzymes like HK2 and PFK1, increasing glycolysis of immune cells and increasing the energy supply for pro-inflammatory behaviors (100). High blood glucose causes lipid accumulation in immune cells via enhanced lipid synthesis, thereby inhibiting FAO. The lipid imbalance promotes the production of pro-inflammatory cytokines and ROS, amplifying the pro-inflammatory immune phenotype. Metabolic metabolites due to higher glycolysis and lipid synthesis (e.g., pyruvate, lactate, acetyl-CoA) act as epigenetic regulators, amplifying the pro–inflammatory gene expression program (101).

Different dietary factors contribute to trained immunity and inflammation. For example, dietary fiber is an anti-inflammatory nutrient that is fermented by gut microbiota to produce short chain fatty acids (SCFAs) such as acetate, propionate, and butyrate; SCFAs bind to GPRs, inhibit pro-inflammatory signaling and promote anti-inflammatory phenotypes (102). Notably, SCFA derivatives such as 4-PBA have emerged as promising translational interventions: recent studies show 4-PBA can robustly rejuvenate innate monocytes and erase low-grade inflammatory memory in CVD-relevant models, representing a novel strategy to reverse sustained signal-induced trained immunity (80). Butyrate has been shown to modulate histone deacetylation, inhibits pro-inflammatory genes and promotes Treg cell differentiation (103). Saturated fatty acids and added sugars are major pro-inflammatory factors and promote diet-induced trained immunity. Trans fats, found in processed foods, are linked to greater systemic inflammation and enhanced monocyte activation, which contribute to CVD risk (104).

Diet-induced trained immunity is a key mechanism underlying long-term CVD risk. Studies show that long-term Western dieters exhibit elevated pro-inflammatory markers (hs-CRP, IL-6 and TNF-α) even after short-term dietary changes. Their monocytes and macrophages display an amplified inflammatory response to external stimuli, and this persistent pro-inflammatory state may drive the development of atherosclerosis and severe cardiovascular events (105). This suggests that short-term diet changes may not reverse the trained immunity phenotype, and long-term dietary intervention can be essential for CVD prevention.

Further research on the regulation of trained immunity by distinct dietary components is warranted to develop precise dietary strategies for CVD management. For example, increasing dietary fiber, omega-3 polyunsaturated fatty acids, or polyphenols may reverse pro-inflammatory diet-induced trained immunity by regulating epigenetic alterations and immune cell metabolism, while reducing saturated fats, trans fats and added sugars may mitigate the induction of pro-inflammatory trained immunity (106). Incorporating SCFA derivatives such as 4-PBA into dietary or therapeutic strategies may also offer a direct approach to erase inflammatory innate immune memory (79, 80). By adjusting dietary recommendations based on an individual’s metabolic and immune profile, CVD can be prevented and managed more effectively.

### 3.2 Fasting, time-restricted eating, and immune-metabolic remodeling

In contrast to nutrient excess, fasting and time-constrained eating (TRE) exert profound positive effects on immune function by reprogramming the body’s metabolic landscape and improving cardiovascular health (107). Fasting includes daily/partial restriction of food intake (alternative-day fasting, 5:2 fasting, or prolonged fasting), and TRE limits food consumption to a narrow daily window (8 hours, 6 hours, or 4 hours), with no caloric intake during the remaining periods. These practices induce a range of adaptive metabolic and immune changes by modifying the body’s energy sources and metabolic rhythms, leading to enhanced metabolic homeostasis and reduced inflammation (108). Fasting redirects systemic energy metabolism toward more efficient utilization. With declining blood glucose and low insulin levels, the body shifts from anabolism to catabolism. Glycogen stores in the liver and muscles are depleted within the first 24–48 hours of fasting, and the body transitions to alternative energy sources (i.e., fatty acids and ketone bodies). Fatty acids are released from adipose tissue and transported to the liver where they undergo β-oxidation to produce energy. The liver converts fatty acids to ketone bodies (acetoacetate, β-hydroxybutyrate and acetone), which serve as an alternative energy source for the brain, heart, and other organs that cannot directly utilize fatty acids (109). This metabolic shift has critical implications for immune function and inflammation. Ketone bodies possess intrinsic anti-inflammatory properties. In particular, they inhibit the activation of pro-inflammatory signaling pathways such as NF-κB and NLRP3 inflammasome, thereby reducing the production of proinflammatory cytokines including IL-1β, IL-6 and TNF-α (110). In addition, ketone bodies can act as a metabolic substrate for immune cells, promoting a shift toward anti-inflammatory phenotypes. For example, β-hydroxybutyrate—the most abundant ketone body—promotes the polarization of macrophages toward the anti-inflammatory M2 phenotype, which is critical for inflammation resolution and tissue repair (111).

Fasting and TRE also modulate immune cell populations and their functional activity. Fasting increases the circulation of pro-inflammatory cells (neutrophils, monocytes) while expanding the number of regulatory T cells (Treg) (112). Fasting-induced autophagy (i.e., the intracellular degradation of damaged organelles and misfolded proteins) further regulates immune cell selection, survival and functional activity. During fasting, autophagy dismantles dysfunctional mitochondria and reduces ROS production in immune cells which prevents pro-inflammatory activation. In monocytes and macrophages, autophagy promotes the clearance of intracellular lipids and damaged proteins and facilitates cellular metabolism and reduces pro-inflammatory cytokine production (113).

TRE aligns food intake with the body’s circadian rhythm, which provides additional benefits for immune-metabolic health. The circadian rhythm regulates many physiological processes including metabolism, immune response and inflammation. Disruption of circadian rhythms (e.g., irregular eating, night shifts, jet lag) is associated with high risk of CVD due to metabolic dysregulation and chronic inflammation (114).

Fasting and TRE synchronize metabolic and immune rhythms by regulating the expression of clock genes (CLOCK, BMAL1, PER, CRY), which control the timing of metabolic enzyme activity, cytokine release and immune cell function. Restricting food intake to the active phase of the circadian cycle (usually daytime) improves metabolic flexibility, insulin sensitivity and mitigates circadian-mediated inflammation (115). In clinical and experimental studies, fasting and TRE are known to confer cardiovascular benefits in animal models of diabetes and heart disease. In animals, fasting and TRE reduce the size of atherosclerotic lesions and plaques and lower the risk of myocardial infarction and stroke. These benefits are mediated by reduced inflammation, improved lipid metabolism, enhanced endothelial function, and attenuated oxidative stress (116). In humans, fasting and TRE can lower body weight, improve insulin resistance, reduce blood pressure, and decrease pro-inflammatory markers such as hs-CRP and IL-6. For patients with metabolic syndrome or existing CVD, fasting and TRE may serve as an adjunct to traditional medical treatment and may reduce residual cardiovascular risk (117).

However, it is important to note that fasting and TRE are not suitable for all individuals and may exert negative effects in specific populations. For instance, some people with malnutrition, frailty or certain chronic diseases (e.g., diabetes mellitus requiring insulin therapy) may experience adverse effects from fasting (e.g., hypoglycemia, electrolyte imbalance, loss of muscle mass, etc.). Additional considerations include the long-term safety and effectiveness of extreme fasting protocols (such as fasting longer than 72 hours) and the need for further research on their long-term effects on cardiovascular health (118). Fasting or TRE as a CVD prevention or management method should take into account individual health status, metabolic characteristics, and lifestyle. Personalized approaches such as adjusting the fasting duration, TRE window and dietary composition might be implemented to maximize benefits and minimize risks. Combining fasting or TRE with a healthy diet (rich in fruits, vegetables, whole grains and lean proteins) and regular physical activity may help enhance their cardiovascular protective effects and support immune-metabolic health (119).

### 3.3 Physical exercise as an immune modulator and cardiovascular protective factor

Exercise is believed to be one of the best lifestyle therapies for cardiovascular health. Apart from being directly beneficial to cardiovascular structure and function (e.g., increased myocardial contractility, improved vascular compliance, reduced blood pressure), exercise also modulates immune function and inflammation to reduce CVD risk (120). Inhibiting the immune-inflammatory response by exercise is a complex process that involves different tissues, organs, and molecular mechanisms.

Another important way to decrease inflammation is by reducing visceral adipose tissue (VAT). VAT is not a passive storage organ but an active endocrine and immune tissue that secretes large amounts of pro-inflammatory cytokines (TNF-α, IL-6 and resistin) and chemokines into the circulation that cause systemic low-grade inflammation, loss of vascular endothelial function, and atherosclerosis (121). Regular physical exercise, particularly aerobic exercise (running, biking, swimming) reduces VAT mass by increasing energy requirements and lipolysis. This reduces the secretion of proinflammatory cytokines and chemokines, alleviating systemic inflammation and reducing cardiovascular burden (122).

Exercise also preserves vascular endothelial function, which is a prerequisite for cardiovascular health. Endothelial dysfunction (ED) is characterized by impaired NO production, excessive ROS production, and increased adhesion molecule expression. Regular exercise promotes endothelial health by increasing the production and bioavailability of NO. NO is a powerful vasodilator that suppresses platelet aggregation, inhibits leukocyte adhesion to the endothelium, and prevents VSMC proliferation—all processes that drive atherosclerosis (123). Regular exercise activates endothelial NO synthase (eNOS) (the enzyme responsible for NO synthesis), via increased shear stress on the vascular wall, AMP-activated protein kinase (AMPK) activation, and reducing oxidative stress; it also lowers the expression of endothelial adhesion molecules (ICAM-1, VCAM-1) and proinflammatory cytokines, thereby eliminating other sources of inflammation (124).

Exercise directly regulates the number and function of immune cells, promoting a non-inflammatory immune system. Regular exercise increases the number of anti-inflammatory cells, like NK cells, Treg cells and M2 macrophages, inhibiting activation of pro-inflammatory cells, such as Th1, Th17 and M1 macrophages (125). For example, increasing activity of NK cells increases the clearance of infected or abnormal cells, which reduces inflammation and tissue damage. In addition, increased number and function of Treg cells promotes enhanced secretion of anti-inflammatory cytokines like IL-10 and TGF-β, which suppress pro-inflammatory responses and promote immune tolerance (126).

Exercise-induced myokines (bioactive peptides secreted by skeletal muscle during exercise) are key mediators of the anti-inflammatory and cardiovascular benefits of exercise: irisin, myonectin, brain-derived neurotrophic factor (BDNF), and IL-6 (anti-inflammatory at physiological levels), as exercise-promoting myokines, enter the circulation and act on target cells (i.e., immune cells, endothelial cells, VSMCs) to regulate their functions and promote health-promoting phenotypes (127). For example, irisin can brown white fat tissue, increase energy consumption, and reduce adiposity-related inflammation. Myonectin can promote lipid metabolism and insulin sensitivity, which can reduce metabolic dysregulation and inflammation. IL-6 secreted by skeletal muscle during exercise stimulates the production of anti-inflammatory cytokines such as IL-10, inhibits the production of pro-inflammatory cytokines like TNF-α, which helps to reduce inflammation (128). Exercise also affects immune cell metabolism, leading to anti-inflammatory metabolic phenotypes. As mentioned above, pro-inflammatory cells (like M1 macrophages and Th17) generate their energy via glycolysis, while anti-inflammatory cells (such as M2 macrophages and Treg cells) rely more on OXPHOS and FAO. Regular exercise promotes mitochondrial function and FAO in immune cells, which leads to macrophage polarization towards the M2 phenotype and enhanced Treg cell function, reducing the production of pro-inflammatory cytokines and ROS, thereby exerting anti-inflammatory effects (129).

The effects of exercise on immune function and cardiovascular health are dose-dependent, and moderate-intensity exercise can often be beneficial. Excessive exercise, such as prolonged high-intensity training without sufficient recovery, can cause immune dysfunction and excessive inflammation due to the release of large amounts of stress hormones (such as cortisol and catecholamines) and proinflammatory cytokines that temporarily impair immune cell function and increase the risk of infection and tissue damage (130). Therefore, an exercise program that includes moderate-intensity aerobic exercise, resistance training, and adequate rest and recovery is desirable. Exercise also plays a significant role in maintaining cardiovascular-immune health in special populations and in extreme conditions. For example, in long-term spaceflight, astronauts are exposed to microgravity which alters vascular structure, decreases immune cell function, and may induce inflammation, leading to impaired cardiovascular structure and function. Exercise training, such as resistance training or aerobic exercise, can mitigate these effects, preserve muscle mass, increase vascular elasticity, help improve immune function and lower inflammation (131). Exercise may also be beneficial in patients suffering from chronic diseases such as type 2 diabetes, hypertension or obesity, maintaining immune-metabolic health, reducing inflammation, and lowering CVD risk (132).

**Figure 3:**
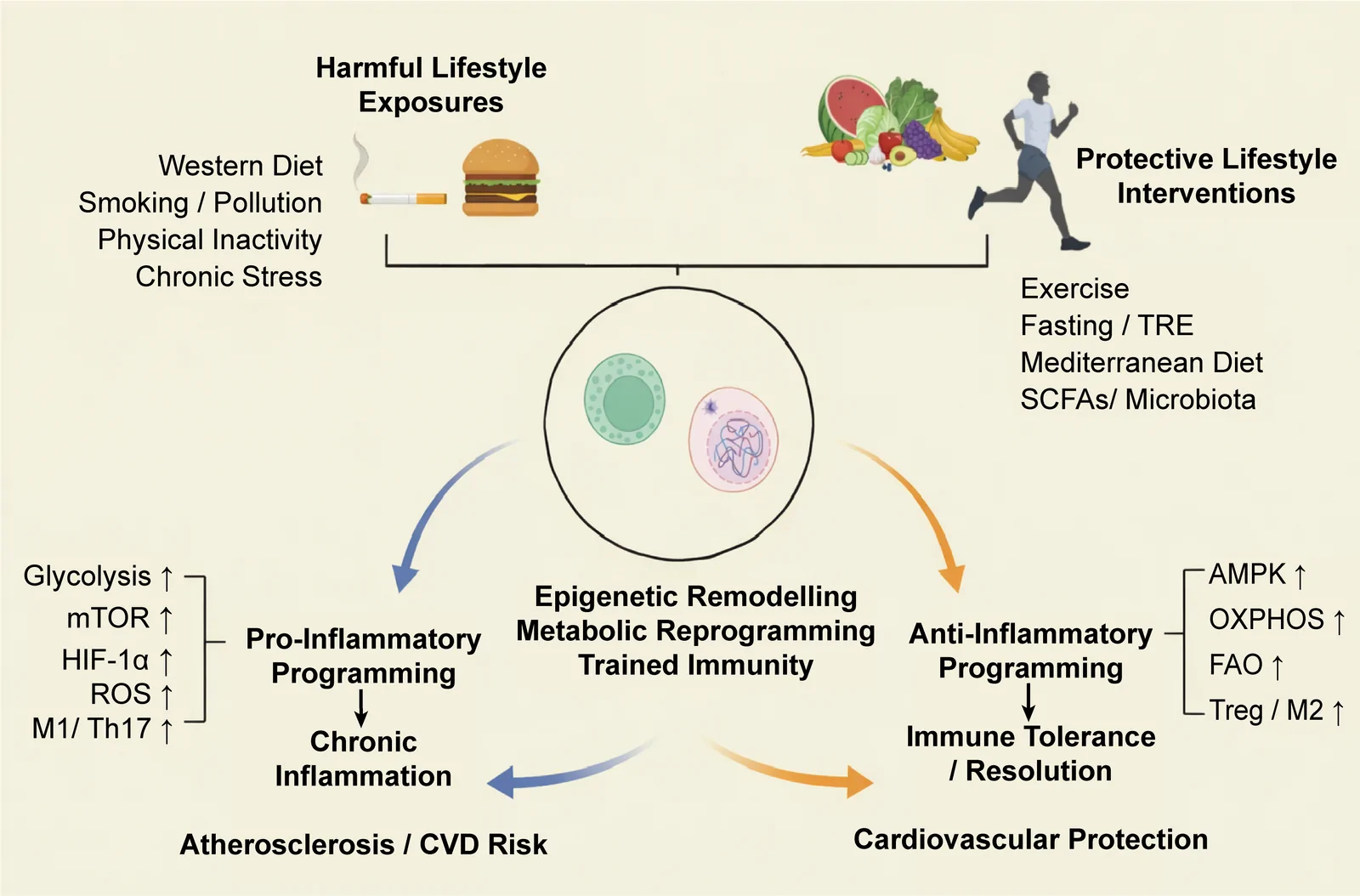
Lifestyle-induced trained immunity and immunometabolic reprogramming in cardiovascular risk modulation. Legend: Harmful lifestyle exposures, including Western diet, smoking or pollution, physical inactivity, and chronic stress, promote pro-inflammatory immunometabolic programming characterized by increased glycolysis, mTOR signaling, HIF-1α activation, and reactive oxygen species (ROS) production, driving M1 macrophage and Th17 cell polarization and contributing to chronic inflammation and atherosclerotic cardiovascular disease (CVD). In contrast, protective lifestyle interventions, such as exercise, fasting or time-restricted eating (TRE), Mediterranean diet, and microbiota-derived metabolites, induce anti-inflammatory programming via activation of AMP-activated protein kinase (AMPK), enhanced oxidative phosphorylation (OXPHOS), and fatty acid oxidation (FAO), promoting regulatory T cell (Treg) and M2 macrophage responses. Epigenetic remodeling, metabolic reprogramming, and trained immunity mediate the long-term effects of lifestyle factors on immune function and cardiovascular risk. AMPK, AMP-activated protein kinase; CVD, cardiovascular disease; FAO, fatty acid oxidation; HIF-1α, hypoxia-inducible factor 1-alpha; mTOR, mechanistic target of rapamycin; OXPHOS, oxidative phosphorylation; ROS, reactive oxygen species; SCFAs, short-chain fatty acids; Th17, T helper 17; Treg, regulatory T cells; TRE, time-restricted eating.

In conclusion, physical exercise exerts effective anti-inflammatory and immunomodulatory effects through multiple pathways, including reducing visceral fat, improving endothelial function, regulating immune cell populations and metabolism, and stimulating myokine production, all of which protect against atherosclerosis and acute cardiovascular events. Regular physical exercise, alongside a healthy diet and other lifestyle modifications, is important for the prevention and management of CVD, as illustrated in **Figure 3**.

## 4 Psychological stress, allostatic load, and inflammatory responses

Other than metabolic and lifestyle, psychosocial stress (a very important and well-understood risk factor) is increasingly recognized in academia and clinical circles as a key factor in CVD development (4, 5, 133). Psychological stress is a personal emotion and a psychophysiological response, that involves measurable biological processes through nervous, endocrine and immune systems (the neuro-endocrine-immune axis), influencing the structure and function of the cardiovascular system and leading to CVD initiation and emergence (134, 135). This chapter presents, more generally, the essential relationship between psychological stress and cardiovascular immune-inflammatory processes, stress biological processes, the allostatic load, and the role of inflammation in stress management, as well as the clinical and preventive significance of stress management.

Psychological stress rapidly activates the body’s stress response (HPA and SNS), and causes neuroendocrine changes. When dealing with a stressful (e.g., an important life event, accident or public speaking) or chronic (e.g., work stress, financial stress, caregiving needs, etc.), the hypothalamus secrete corticotropin-releasing hormone (CRH) to stimulate the pituitary gland to release adrenocorticotropic hormone (ACTH), and it invades the adrenal cortex to produce glucocorticoid (GC)(e. g., cortisol). It also stimulates SNS to release catecholamines (e–g., adrenaline and noradrenaline) from the adrenals medulla and sympathetic nerve areas (136). In short-term, this stress reaction helps the body pump up energy, alertness, and cope with stress. Chronic or repeated stress disrupts the balance of HPA axis and SNS, which causes long-term neuroendocrine activation, persistent immune system stimulation, and chronic inflammatory responses to the heart (137). One key consequence of chronic stress is glucocorticoid resistance (GCR). GCs, especially cortisol, normally produce strong anti-inflammatory effects by inhibiting pro-inflammatory signaling such as NF-κB and AP-1, reducing the production of pro-iodytic cytokines (TNF-α, IL-6, IL1β) and increasing the production (IL-10) (138). Under chronic stress, immune cells are less sensitive to GCs and thus this anti-iotic feedback mechanism is suppressed. The mechanisms of GCR are downregulation of GR expression, impaired GR nuclear translocation, and high expression of GR antagonists. Pro-iocytic cytokine levels remain high, thus in chronic low-grade inflammation damaging vascular endothelium, atherosclerosis and CVD (139). Chronic SNS can also contribute to the inflammation and damage of a heart in a number of ways. Catecholamines bind to adrenergic receptors (α,β) on the immune cells, which regulate their growth, differentiation and activation. For example, there is strong activation of β2-adrenergic receptors on monocytes and macrophages that produce pro-inflammatory cytokines and ROS, whereas activation of α-adenergic receptors promotes the recruitment of inflammatory cells to the vascular wall (140). In response to the blood vessel, long term SNS activation stimulates bone marrow hematopoiesis and induces the production and release of inflammatory (neutrophils, monocytes) cells within the blood. These cells attach to the endothelium, interact with adhesion molecules, penetrate the vascular barrier, and contribute to vascular inflammation and atherosclerotic plaque formation (141). Besides direct effect for immune cells, chronic stress also regulates immune-inflammatory response by changing gut microbiota composition. The gut microbiota – the largest living organism – is closely connected with the immune system through the “gut-immune axis.” Chronic stress disrupts gut microbiota production, which produces extra pro-inflammatory bacteria and fewer beneficial bacteria (e.g., Bifidobacterium and Lactobacillus) (142). This gut dysbiosis disrupts intestinal barrier function and allows food antigens, bacteria, or toxins to enter the blood (the “leaky gut”). The foreign drugs cause systemic inflammation, which increases vascular inflammation and increases CVD risk (143).

Allostatic load was introduced by McEwen & Stellar (1993) as a measure of the cumulative cost that the body must pay for maintaining homeostasis during long-term adaptation to stress (psychological, environmental, and metabolic) (144). The allostatic burden is a modification of the previously described single risk factors (hypertension, hyperglycemia or dyslipidemia). Allostatic load is a reflection of the inherent dysregulation of several physiological systems, such as neuroendocrine, metabolic, immune and cardiovascular systems, and it aggregates information of such systems (blood pressure, heart rate, cortisol, blood glucose, blood lipids, and inflammatory cytokines) to measure the overall damage of chronic stress (145).

Allostatic load is calculated by measuring the degree of deviation from normal ranges for each indicator, and summing up such deviations. Higher allostatic score values indicate higher overall physiological damage and higher risk of adverse health effects, including CVD (146). Epidemiological studies have consistently shown that allostatic score has a strong positive relation with CVD risk. For example, a long-term follow-up study of middle aged adults found that older adults with high allostatic score have a higher probability of developing myocardial infarction, stroke and heart failure as a function of CVD factors (147). Another study observed that allostatic load increases CVD mortality in older adults after adjustment for age, gender and other confounding factors (148).

From an immunological point of view, allostatic load is associated with chronic low grade inflammation, which is a biological correlate of allostatic burden with cardiovascular health. Chronic stress leads the neuro-endocrine-immune system to activate immune cells, produce more pro-inflammatory cytokines, and develop chronic inflammation, thus damaging the cardiovascular system by the following mechanisms (e.g., endothelial dysfunction, VSMC proliferation, platelet activation, and thrombus formation) (149).

High allostatic load results in a higher number and activation of neutrophils, proinflammatory cytokine production, and poor anti-inflammatory responses. Neutrophils are among the most abundant leukocytes, playing an important role in inflammation and thrombosis. High allostatic load increases neutrophil production in bone marrow and mobilization to the periphery. Activated neutrophils release pro-inflammatory cytokines, ROS, and proteases, which damage vascular endothelial cells, promote platelet adhesion and aggregation, and cause atherosclerosis; additionally, neutrophils form NETs, which promote thrombus formation and vascular inflammation, leading to acute cardiovascular damage (150).

High allostatic load may also lead to higher expression of pro-inflammatory and lower expression of anti-inflammatory genes. Chronic stress-induced epigenetic changes (e.g., histone acetylation and methylation) may lead to higher transcriptional activity of proinflammatory genes (TNF-α, IL-6, IL-1β) and decreased expression of anti-inflammatory genes (IL-10, TGF-β), which are stable and heritable, and this is the cause of chronic inflammation and a high risk of CVD (151).

The concept of allostatic load provides a model to understand the impact of multiple stresses on cardiovascular health. Using indicators from multiple physiological systems, it captures the interplay between psychological stress, metabolic dysfunction and immune-inflammatory responses, and provides a deeper view of CVD risk than single risk factors. Allostatic load can be used for cardiovascular risk stratification and early-warning for patients with multiple risk factors or chronic stress (152).

From a clinical point of view, psychological stress is an important risk factor for CVD. Stress management and treatment can reduce inflammation, improve cardiovascular health, and prevent CVD in a systematic manner (153). Stress management has become a part of comprehensive CVD prevention and treatment and has become increasingly important both from a clinical and public health perspective. Many studies show that stress management tools including cognitive-behavioral therapy (CBT), meditation, relaxation, exercise, and social support can reduce inflammation and improve CVD outcomes (154).

CBT helps people recognize irrational thoughts and behaviors associated with stress and relieve psychological stress and anxiety. CBT decreases IL-6, TNF-α, stress hormones (cortisol), and improves endothelial function and blood pressure in people with CVD or at high risk of developing CVD (155).

Mindfulness Meditation and Relaxation (deep breathing, progressive relaxation and yoga) improve the autonomic nervous system function by decreasing the activity of the SNS and increasing the parasympathetic activity, decreasing the levels of stress hormone production, reducing oxidative stress and improving immune function. A meta-analysis of randomized controlled trials revealed that mindfulness-based treatments reduce the levels of hs-CRP and other pro-inflammatory markers and improve cardiovascular risk factors (156).

Exercise therapy exerts positive effects on immune-inflammatory function, improves emotional health and reduces stress. Regular exercise stimulates the release of endorphins (natural mood-enhancing chemicals) to reduce anxiety and depression, decreasing psychological burden. Exercise also provides a relief from stress and breaks the cycle of overeating or smoking (157).

Social support mitigates the psychological impact of stress: high social support facilitates emotional support, practical support and a sense of belonging. Individuals with sufficient social support have lower levels of pro-inflammatory cytokines and stress hormones and a reduced CVD risk. Social support interventions, such as support groups or peer counselling, can help people cope with stress and improve cardiovascular health (158).

Psychosocial assessment could be used to help identify high-risk people and provide targeted interventions. Current CVD risk assessment tools rely only on physiological cues (blood pressure, blood glucose, lipids) and may miss psychosocial signals. Psychological stress assessment scales such as the Perceived Stress Scale (PSS), the Beck Depression Inventory (BDI) or the State-Trait Anxiety Inventory (STAI) and markers of allostatic load (inflammatory cytokines, cortisol levels) can help clinicians to assess CVD risk in high-risk individuals (159). For high-stress and high allostatic load individuals, timely stress management interventions (psychological counseling, CBT or relaxation training) can be carried out alongside risk factor control to improve CVD outcomes and prognosis (160). Stress management also has important public health implications. As our lives become more demanding and stressful, stress-related CVD is becoming more common. Public health activities such as mental health education, workplace stress management and community-based support can contribute to reducing the population-level burden of stress-related chronic disease, raise public awareness of the link between stress and cardiovascular health, promote stress management skills and foster healthy coping strategies (161).

Health care providers have to integrate mental health into cardiovascular care to optimize patient management. For example, cardiovascular clinicians may receive training in basic psychological assessment and intervention skills, or mental health providers (psychiatrists, psychologists, counselors) may offer integrated care. By addressing both the clinical/psychological aspects of CVD, health care providers can improve treatment efficacy and reduce medical costs (162).

In conclusion, psychological stress affects cardiovascular inflammatory responses via the neuro-endocrine-immune network and this pathological process is reflected in allostatic load. Elucidating the role of psychological stress in immune-inflammatory CVD and implementing stress management for prevention and treatment will help build a comprehensive and effective CVD management model to reduce CVD incidence and mortality.

## 5 Special exposures and drug-related cardiovascular-immune injury

Besides lifestyle and psychosocial factors discussed earlier, other external events, such as drugs, environmental pollutants, and some medications, can directly or indirectly injure the cardiovascular system through immune and inflammatory responses, causing serious or chronic cardiovascular events (163, 164). The mechanisms that drive these injuries include immune cell activation, improved inflammatory responses and endothelial injury, and metabolic dysregulation. These are major, non-trivial factors contributing to CVD which need to be more investigated by clinicians and researchers.

### 5.1 Environmental triggers of cardiovascular-immune damage

Environment (Air pollution, heavy metals, persistent organic pollutants) are a major exogenous cause of cardiovascular-immune damage. In recent years, industrialization and urbanization are increasingly taking place with high levels of exposure which is a public health challenge (165). PM2.5 (fine particles of less than 2.5 m diameter) may contribute to higher CVD risks. It may penetrate into the respiratory tract, enter the blood, and enter different organs, including heart and blood vessels (163). PM2. 5 particles bind harmful chemicals like heavy metals, PAHs or endotoxins, act as PAMPs or DAMPs, which activate the immune system. PM 2. 5 activates macrophages and neutrophils, releasing pro-inflammatory cytokines (TNF-alpha, IL-6, IL + 1β), ROS and chemokines that result in vascular inflammation, endothelial dysfunction and platelet aggregation and are at risk for atherosclerosis, myocardial infarction and stroke (166). PM2.5 also leads to NET formation causing thrombosis and vascular damage. PM2.5 further disrupts gut barrier and causes systemic inflammation and cardiovascular injuries (167).

High-level metals such as lead, mercury and cadmium harm the heart and immune system. Though lead levels are low, it still increases the CVD risk. Lead is accumulated over time in vascular endothelial cells, promotes oxidative stress and inflammation. It is anoxic to immune cells. It may interfere with the number and activity of T cells, B cells and NK cells and lead to pro-inflammatory cytokines. Lead increases blood pressure by reducing the level of endothelial NO production and promoting vascular smooth muscle contraction (168). Mercury is a source of oxidative stress or inflammation in cardiomyocytes and vascular endothel cells, causing myocardial bleeding, arrhythmias and atherosclerosis. Cadmium impairs lipid metabolism, endothelial dysfunction and platelet aggregation, which increases risk of hypertension and coronary heart disease (169).

Persistent organic pollutants (POPs)—prophilic PBs, DDT, and dioxins—are lipophilic and persistent compounds that bioaccumulate in food. POPs bind to the aryl hydrocarbon receptor (AhR) of immune cells and endothelial cells and act as pro-inflammatory signaling mechanisms and express pro-inositive cytokines and ROS. They disrupt immune cells, suppress anti-inflammatory reactions, and promote pro-infertile reactions (170). The POPs also impair endothelial function, promote VSMC proliferation and increase platelet aggregation, which cause atherosclerosis and CVD (171).

### 5.2 Drug-induced cardiovascular-immune injury: cardio-oncology focus

In addition to endogenous immunoinflammatory mechanisms, cardiovascular injury may also arise secondary to pharmacological or environmental exposures. Increasing recognition of therapy-induced cardiotoxicity—particularly in oncology—has led to the development of cardio-oncology, a multidisciplinary field focused on the prevention and management of cardiovascular complications associated with cancer treatment (172). Many anticancer agents, immunomodulators, and certain anti-inflammatory drugs can disrupt immune–vascular homeostasis and trigger inflammatory or thrombotic cardiovascular events.

Cancer immunotherapies, especially immune checkpoint inhibitors (ICIs) and chimeric antigen receptor T-cell (CAR-T) therapy, enhance antitumor immunity by stimulating T-cell activation (173). Notably, ICIs and CAR-T therapy exhibit distinct mechanistic pathways of cardiovascular toxicity, a critical distinction for clinical management: ICIs targeting PD-1, PD-L1, or CTLA-4 cause cardiovascular toxicity via adaptive immune overactivation: they remove inhibitory signals that normally restrain T-cell responses, and this immune disinhibition can promote myocarditis, pericarditis, arrhythmias, and heart failure (174). ICI-associated myocarditis, though relatively uncommon, carries high mortality and is characterized by T-cell infiltration of the myocardium, elevated cardiac biomarkers (e.g., troponin), and increased inflammatory cytokines. Early recognition and prompt immunosuppressive therapy, typically with corticosteroids, are critical for management (85).

CAR-T therapy induces cardiovascular toxicity primarily through innate immune cell dysregulation, driving systemic inflammatory complications (85). Cytokine release syndrome (CRS), driven by massive cytokine production, can cause hypotension, endothelial dysfunction, capillary leakage, and thromboinflammation, ultimately leading to cardiac dysfunction or shock (85). Anti-cytokine therapies, such as IL-6 receptor blockade with tocilizumab, are commonly used to mitigate these effects (175).

Certain non-oncologic drugs may similarly influence cardiovascular risk through inflammatory or thrombotic mechanisms. For example, long-term or high-dose nonsteroidal anti-inflammatory drugs (NSAIDs) inhibit cyclooxygenase enzymes, disrupt prostaglandin balance, and may increase blood pressure, promote fluid retention, and elevate the risk of myocardial infarction or stroke, particularly in susceptible individuals (176).

### 5.3 Immunometabolic effects of modern cardiometabolic drugs

Modern cardiometabolic drugs represent an important class of therapeutics that directly modulate immune-metabolic interactions in CVD, extending beyond their traditional metabolic effects (1):. GLP-1 Receptor Agonists (e.g., Semaglutide): Recent clinical evidence confirms that semaglutide reduces major adverse cardiovascular events, and its cardioprotective effects are primarily mediated by reduced systemic inflammation rather than just weight loss (83, 177). GLP-1 agonists inhibit pro-inflammatory macrophage polarization, reduce cytokine production (IL-6, TNF-α), and improve endothelial function by suppressing immune-metabolic dysregulation in the vascular microenvironment (178) (2). SGLT2 Inhibitors: These drugs directly target immune-metabolic crosstalk in CVD: they reduce glycolytic reprogramming in pro-inflammatory immune cells, mitigate mitochondrial ROS production and lipotoxicity in vascular and immune cells, and suppress thrombo-inflammatory pathways by inhibiting platelet activation and NET formation (84, 179). SGLT2 inhibitors also improve metabolic homeostasis in adipose tissue, reducing the secretion of pro-inflammatory adipokines and alleviating systemic meta-inflammation (180).

Recognition of therapy-related immunoinflammatory cardiotoxicity has important clinical implications. Patients receiving potentially cardiotoxic treatments should undergo baseline cardiovascular risk assessment and longitudinal monitoring of cardiac function and inflammatory biomarkers. Early detection, dose adjustment, and timely intervention—including immunosuppressive or cardioprotective strategies—may reduce morbidity and improve outcomes (181).

Overall, understanding drug-induced immune–vascular interactions not only improves safety management but also provides additional insight into how dysregulated immunity contributes to cardiovascular pathology.

## 6 Immune-related biomarkers and clinic translation

Immune-related biomarkers, such as composite indexes from immune cell counts and single inflammatory cytokines, are useful to predict CVD risk, disease severity, and treatment decisions (182). They reflect the immune-inflammatory states of the body and reveal the pathological processes involved in CVD. In this chapter, we discuss the role of immune-related markers in predicting CVD Risk, as well as their limitations and opportunities.

Infrastructural indices based on immune cell values (for example, SIRI, NLR and PLR) can predict adverse cardiovascular events (183). The immune cell-to-infrastructures incorporate proportional relationships between different immune cells to give a more complete picture of a body’s immune-inflammatory status than single-cell counts. SIRI is typically defined as (Neutrophil count + Monocyte count + Lymphocyte count) which indicates the balance between pro-inflammatory cells (neutrophils, monocytes) and anti-inflammatory, (lymphocytes). A higher SIRI indicates greater pro- inflammatory cell and lower anti- inflammatory cells suggesting a chronic inflammation (184). There have been several clinical studies that showed predictive value of SIRIs in CVD. In ACS patients, more than half of deaths were in-hospital, followed by recurrent myocardial infarction and heart failure. A meta-analysis on observational studies suggested SIREs may be a key factor for adverse cardiovascular events in comparison to traditional risk factors such as age, sex and lipid level (185). SIRIS also predicts long-term heart failure and hypertension in patients with chronic heart failure as well as hypertension. STRIs are used in different CVD subtypes. NLR, the number of neutrophils/lymphocytes is a simpler index that shows neutrophil (pro-inflammatory) and lymphocyte (anti-inflammatory) populations (186). Higher NLR leads to increased systemic inflammation, endothelial dysfunction and atherosclerosis. It has been shown that NLR can lead to adverse outcomes for ACS patients, stable coronary artery disease, and stroke. For example, in PCI patients, higher NLR lead to increased risk of stent thrombosis, repeated ischemia and mortality. NLR also predicts carotid artery stenosis and increased risk for cardiovascular events in aging individuals (187). It can be used to estimate risk in general.

Multi-omics technologies have revolutionized our understanding of the complex molecular networks underlying cardiovascular diseases (CVDs). By integrating genomics, transcriptomics, proteomics, metabolomics, epigenomics, and immunomics, it is now possible to comprehensively map the pathways that drive immune dysregulation, chronic inflammation, and metabolic remodeling (188). This systems-level perspective not only deepens mechanistic insight but also facilitates the identification of novel biomarkers, therapeutic targets, and precision intervention strategies.

Genomic analyses provide important insights into genetic susceptibility to immunoinflammatory CVD. Genome-wide association studies (GWAS) have identified numerous loci linked to inflammatory signaling pathways and cardiovascular risk. Variants in genes encoding Toll-like receptors (TLRs), interleukins, cytokine receptors, and innate immune regulators have been associated with atherosclerosis, myocardial infarction, and stroke (189). These polymorphisms may influence immune cell activation, cytokine production, and individual responses to environmental exposures such as diet, stress, and pollution. Mendelian randomization studies further enable causal inference and target prioritization. For example, loss-of-function variants in the IL-6 receptor gene are associated with reduced cardiovascular risk, supporting IL-6 pathway inhibition as a potential therapeutic strategy (190).

Transcriptomics characterizes global gene expression changes and reveals dynamic inflammatory signatures in vascular tissues and immune cells. Bulk RNA sequencing of atherosclerotic plaques has identified transcriptional programs associated with plaque instability, macrophage activation, and endothelial dysfunction, including upregulation of pro-inflammatory mediators such as TNF-α, IL-1β, and CXCL8, alongside reduced expression of anti-inflammatory genes such as IL-10 and TGF-β (191).

Single-cell RNA sequencing (scRNA-seq) has further refined this understanding by resolving cell-type–specific heterogeneity within plaques. These studies have uncovered distinct macrophage subsets, exhausted T cells, and other rare populations that critically influence lesion progression and thrombosis (192). Integrative transcriptomic analyses also enable identification of co-expression networks that link immune activation with metabolic reprogramming.

Because proteins are the direct effectors of biological function, proteomics complements transcriptomics by providing functional readouts of disease activity. Proteomic profiling of plasma, serum, and vascular tissues has identified numerous inflammatory and thrombotic mediators associated with adverse outcomes, including C-reactive protein (CRP), myeloperoxidase (MPO), and platelet-derived growth factor (PDGF) (193). Proteomics additionally enables detection of post-translational modifications such as phosphorylation, acetylation, and glycosylation, which critically regulate protein activity. For instance, enhanced phosphorylation of NF-κB promotes transcription of pro-inflammatory genes within atherosclerotic lesions. Integration of proteomic and transcriptomic data often reveals discordance between mRNA and protein levels, highlighting the importance of post-transcriptional regulation (194).

Metabolomics captures small-molecule metabolites and provides a direct snapshot of cellular metabolic states. Immunometabolic reprogramming is increasingly recognized as a central driver of cardiovascular inflammation. Patients with CVD frequently exhibit dysregulated lipid metabolism, enhanced glycolysis, and altered amino acid profiles, reflecting metabolic stress within immune cells (195). Importantly, metabolites also function as signaling molecules. Succinate accumulation in activated macrophages stabilizes hypoxia-inducible factor-1α (HIF-1α) and promotes IL-1β production, whereas itaconate exerts anti-inflammatory effects by suppressing NF-κB signaling (69). Integrating metabolomic data with transcriptomic and proteomic information helps elucidate how metabolic pathways shape immune cell behavior and inflammatory responses.

Epigenomic regulation—including DNA methylation, histone modifications, and non-coding RNAs—plays a critical role in long-term immune memory and trained immunity. These reversible modifications mediate the sustained effects of lifestyle and environmental exposures on cardiovascular risk (196). Chronic metabolic stress or unhealthy diets can induce epigenetic remodeling of pro-inflammatory genes, enhancing their transcriptional accessibility. Histone acetylation and methylation patterns modulate inflammatory gene expression within vascular tissues. Non-coding RNAs, such as miRNAs and long non-coding RNAs (e.g., miR-155, miR-21, ANRIL), further regulate immune-metabolic signaling networks (197). Integrating epigenomic data with other omics layers may reveal modifiable regulatory mechanisms and potential targets for epigenetic therapy.

Immunomics focuses on high-dimensional characterization of immune cell populations and functions. Technologies such as flow cytometry, mass cytometry (CyTOF), and single-cell sequencing enable detailed profiling of circulating and tissue-resident immune cells in CVD (198).

These approaches have revealed diverse macrophage subsets, activated neutrophils, dysfunctional regulatory T cells, and pro-inflammatory monocyte populations that collectively drive vascular inflammation. Functional assessments—including cytokine secretion, phagocytosis, and cytotoxicity—provide additional insight into immune competence. Integrating immunomic data with other molecular layers helps connect cellular phenotypes to underlying regulatory pathways (199).

The true power of multi-omics lies in integrative analysis. Computational tools—including machine learning, network biology, and pathway modeling—enable identification of key regulatory hubs and disease-driving pathways. For example, integrated analyses frequently highlight NF-κB signaling, NLRP3 inflammasome activation, and metabolic–inflammatory circuits as central nodes in CVD pathogenesis (200). Multi-omics–based predictive models that combine genetic variants, transcriptomic signatures, proteomic markers, and metabolic profiles have demonstrated improved risk stratification compared with traditional clinical factors alone. Moreover, such approaches may guide personalized therapy, allowing patients with dominant inflammatory phenotypes to receive immunomodulatory treatments, while those with metabolic dysfunction may benefit more from metabolic-targeted interventions (201). Overall, multi-omics strategies provide a systems-level framework that bridges molecular mechanisms with clinical translation and represent a cornerstone of future precision cardiovascular medicine.

Liquid biopsy, defined as the non-invasive analysis of circulating components in biological fluids such as blood, urine, or saliva, has recently emerged as a promising tool for cardiovascular risk prediction and dynamic disease monitoring (202). Unlike traditional biomarkers that provide static snapshots of pathology, liquid biopsy enables real-time assessment of vascular injury, immune activation, and systemic inflammation. Circulating biomarkers—including extracellular vesicles, immune cell phenotypes, and cell-free nucleic acids—offer valuable insights into ongoing immunoinflammatory processes and may facilitate earlier detection and more precise management of cardiovascular diseases (CVDs) (203).

Extracellular vesicles (EVs), particularly exosomes (30–150 nm), are membrane-bound vesicles secreted by immune cells, endothelial cells, platelets, and cardiomyocytes. These vesicles carry bioactive cargo, including proteins, lipids, microRNAs (miRNAs), and messenger RNAs, which mediate intercellular communication and modulate inflammatory and metabolic signaling pathways (204). In patients with CVD, exosomes derived from immune or vascular cells often contain pro-inflammatory mediators such as TNF-α, IL-6, and miR-155, which promote endothelial dysfunction, plaque progression, and thromboinflammation. Conversely, exosomes from healthy individuals may carry anti-inflammatory molecules, including IL-10 and miR-146a, that help maintain vascular homeostasis (205). Profiling exosomal cargo has therefore emerged as a potential strategy for disease stratification. For example, elevated circulating miR-155 levels have been associated with atherosclerotic burden and plaque instability, suggesting its utility as a risk biomarker (206).

Beyond diagnostics, exosomes are also being investigated as therapeutic delivery vehicles due to their intrinsic biocompatibility and targeting capacity. Engineered vesicles carrying anti-inflammatory agents or regulatory RNAs may enable targeted modulation of vascular inflammation.

Circulating immune cells play central roles in cardiovascular inflammation and represent another important component of liquid biopsy. Quantitative and functional characterization of immune cell subsets using flow cytometry or mass cytometry (CyTOF) provides direct information about systemic immune status (207).

Elevated proportions of pro-inflammatory monocytes (CD14++CD16+), neutrophil activation, increased neutrophil extracellular trap (NET) formation, and enhanced reactive oxygen species production have all been associated with plaque instability, thrombosis, and acute coronary syndromes. Composite indices integrating immune cell counts with conventional markers—such as the neutrophil-to-lymphocyte ratio (NLR) and high-sensitivity C-reactive protein (hs-CRP)—may improve cardiovascular risk stratification and prognostic accuracy (208).

Cell-free DNA (cfDNA) and cell-free RNA (cfRNA), released from injured or dying endothelial cells, immune cells, and cardiomyocytes, provide additional non-invasive indicators of tissue damage and inflammation (209). Tissue-specific methylation signatures in cfDNA can help identify the cellular origin of injury, while cfRNA transcripts and circulating miRNAs reflect active inflammatory and metabolic processes. Elevated levels of pro-inflammatory transcripts and cardiac-specific nucleic acids have been reported in acute coronary syndromes and heart failure, correlating with disease severity and outcomes. These circulating nucleic acids therefore offer a dynamic method for monitoring vascular injury and therapeutic responses (210).

A major advantage of liquid biopsy is the potential for longitudinal monitoring. Serial assessment of exosomal miRNAs, immune phenotypes, or circulating nucleic acids may allow clinicians to track treatment response, evaluate anti-inflammatory or lipid-lowering therapy efficacy, and detect early signs of disease progression. Such dynamic monitoring could enable timely therapeutic adjustments and personalized management (211). Despite its promise, several challenges must be addressed before widespread clinical implementation. Standardized protocols for sample collection, processing, and analysis are lacking, and many candidate biomarkers require validation in large, multi-center cohorts. In addition, technical complexity and cost currently limit routine clinical use. Continued technological advances and methodological harmonization will be essential to translate liquid biopsy into practical cardiovascular care (212). Overall, liquid biopsy–based diagnostics represent a rapidly evolving frontier that may complement traditional risk factors and enhance precision cardiovascular medicine.

Future cardiovascular prevention strategies should shift from single risk-factor control toward a systems-based model integrating immune, metabolic, and lifestyle profiling. Personalized lifestyle prescriptions, digital monitoring, and artificial intelligence-assisted prediction models may facilitate early identification of high-risk individuals and optimize individualized interventions (213). Ultimately, incorporation of immunometabolic principles into routine cardiovascular care may reduce residual risk and improve long-term outcomes.

## 7 Future directions

While trained immunity has become a convincing concept to link environmental stimulus to chronic inflammation, several issues remain. Most results have been provided by *in vitro* or animal models, and the durability of trained immunity re-training in humans is not clear (7). Emerging research has refined the trained immunity concept to emphasize dosage/duration-dependent innate immune memory and central/peripheral subtypes, but the clinical relevance of these subtypes in human CVD, as well as the reversibility of sustained signal-induced inflammatory memory, remains to be elucidated (92). And it remains unclear if trained immunity uniformly contributes to cardiovascular diseases or might exert context-dependent protective effects. The discovery of trained immunity has opened new avenues for investigating its causal role in human diseases, highlighting the need for further research beyond associative findings (90). Additionally, the translational potential of SCFA derivatives (e.g., 4-PBA) in erasing inflammatory innate immune memory requires further validation in human clinical trials (80).

Although NET formation has been found to have a negative influence on plaque and thrombosis, it is not certain whether NETs are causes or a primary factor in inflammation. Furthermore, if NET formation is inhibited pharmacologically, it could potentially impede host defense against infection and safety issues (51).

Allostatic load provides a framework for understanding the cumulative biological impact of chronic stress across multiple physiological systems, with ongoing research needed to establish standardized measurement approaches and validate its clinical utility in cardiovascular populations (152).

Multi-omics methods provide unprecedented information about immunometabolic networks, but whether the high dimensional data provide additional information than the traditional cardiovascular risk score is not clear. Cost and interpretability are also important constraints for clinical implementation (201). Future research should also integrate the study of CHIP and the immunometabolic mechanisms of cardiometabolic drugs (GLP-1 agonists, SGLT2 inhibitors) into multi-omics models to improve risk stratification precision (214, 215).

## 8 Conclusions

Cardiovascular disease is increasingly treated not only as a chronic disease of lipid accumulation or the blood vessel failure, but also a chronic immunometabolic disease driven by persistent low-grade inflammation and maladaptive responses (9). Age-related genetic immune changes (e.g., CHIP), innate immune memory shaped by the dosage and duration of pro-inflammatory signals, and central/peripheral trained immunity subtypes further expand our understanding of the heterogeneous pathogenesis of CVD (3, 21, 92). Notably, SCFA derivatives such as 4-PBA show promise in rejuvenating innate monocytes and erasing inflammatory memory, representing a novel translational direction for reversing sustained signal-induced trained immunity (80). The concept of allostatic load, first introduced by McEwen and elaborated by Juster et al., provides a framework for understanding how chronic stress becomes biologically embedded through the cumulative dysregulation of multiple physiological systems (152). The recent discovery of distinct cardiovascular toxic mechanisms of ICIs (adaptive immune overactivation) and CAR-T therapy (innate immune dysregulation) refines cardio-oncology management (85). The integration of CHIP screening and trained immunity characterization into precision immunocardiology frameworks is expected to shape cardiovascular prevention and therapy in the coming decade (2, 92).

In conclusion, cardiovascular disease should be treated as a dynamic immunometabolic network disorder characterized by chronic inflammation, metabolic adaptation, environmental conditions and psychosocial stress. This can facilitate risk prediction and offer novel and individualized treatments.

## Glossary

4-PBA: 4-Phenylbutyric Acid
ACS: Acute Coronary Syndromes
ACTH: Adrenocorticotropic hormone
AMPK: AMP-activated protein kinase
AP-1: Activator protein 1
CAR-T: Chimeric Antigen Receptor T-cell
CHIP: Clonal Hematopoiesis of Indeterminate Potential
CBT: Cognitive-behavioral therapy
CRP: C-reactive protein
CVD: Cardiovascular Disease
cGAS-STING: Cyclic GMP-AMP synthase-Stimulator of Interferon Genes
CRS: Cytokine release syndrome
CyTOF: Mass cytometry
DAMPs: Damage-Associated Molecular Patterns
DCs: Dendritic cells
DIC: Disseminated intravascular coagulation
ED: Endothelial dysfunction
EVs: Extracellular vesicles
FAO: Fatty acid oxidation
FXa: Factor Xa
FVIIa: Factor VIIa
GC: Glucocorticoid
GCR: Glucocorticoid Resistance
GLP-1: Glucagon-Like Peptide-1
GWAS: Genome-wide association studies
HIF-1α: Hypoxia-Inducible Factor-1α
HDAC: Histone deacetylases
HK2: Hexokinase 2
HPA: Hypothalamic-Pituitary-Adrenal
hs-CRP: High-sensitivity C-reactive protein
ICAM-1: Intercellular adhesion molecule 1
ICIs: Immune Checkpoint Inhibitors
IL: Interleukin
LPS: Lipopolysaccharide
MAPK: Mitogen-activated protein kinase
MCP-1: Monocyte chemoattractant protein-1
MI: Myocardial infarction
MMPs: Matrix metalloproteinases
mito-DAMPs: Mitochondrial Damage-Associated Molecular Patterns
mTOR: Mammalian Target of Rapamycin
MPO: Myeloperoxidase
NETs: Neutrophil Extracellular Traps
NF-κB: Nuclear Factor-Kappa B
NLR: Neutrophil-to-lymphocyte ratio
NLRP3: NOD-like receptor pyrin domain-containing 3
NO: Nitric oxide
eNOS: Endothelial NO synthase
NSAIDs: Nonsteroidal anti-inflammatory drugs
OXPHOS: Oxidative Phosphorylation
PAF: Platelet-activating factor
PAI-1: Plasminogen activator inhibitor-1
PARs: Protease-activated receptors
PAMPs: Pathogen-Associated Molecular Patterns
PDGF: Platelet derived growth factor
PFK1: Phosphofructokinase 1
PI3K-AKT: Phosphatidylinositol 3-kinase-protein kinase B
PM2.5: Fine particulate matter (≤2.5 μm)
PLR: Platelet-to-lymphocyte ratio
POPs: Persistent organic pollutants
PSGL-1: P-selectin glycoprotein ligand-1
ROS: Reactive Oxygen Species
SCFAs: Short Chain Fatty Acids
SGLT2: Sodium-Glucose Cotransporter 2
SIRI: Systemic Immune-Inflammation Index
SNS: Sympathetic Nervous System
tPA: Tissue-type plasminogen activator
TGF-β: Transforming growth factor-β
TLR4: Toll-like receptor 4
TNF-α: Tumor Necrosis Factor-α
TRE: Time-Restricted Eating
TXA2: Thromboxane A2
Treg: Regulatory T cells
VAT: Visceral adipose tissue
VCAM-1: Vascular cell adhesion molecule 1
VEGF: Vascular endothelial growth factor
VSMC: Vascular Smooth Muscle Cell
oxLDL: Oxidized low-density lipoprotein
cfDNA: Cell-free DNA
cfRNA: Cell-free RNA
LDHA: Lactate dehydrogenase A
AhR: Aryl hydrocarbon receptor
BDNF: Brain-derived neurotrophic factor
CRH: Corticotropin-releasing hormone
PER: Period
CRY: Cryptochrome
CLOCK: Circadian locomotor output cycles kaput
BMAL1: Brain and muscle ARNT-like 1
DNase: Deoxyribonuclease
PAD4: Peptidylarginine deiminase 4
GR: Glucocorticoid receptor
IFN-γ: Interferon-γ
Th1: T helper 1
Th17: T helper 17
NK cells: Natural killer cells
irisin: A myokine
myonectin: A myokine
PSS: Perceived Stress Scale
BDI: Beck Depression Inventory
STAI: State-Trait Anxiety Inventory
CAD: Coronary artery disease
PCI: Percutaneous coronary intervention
scRNA-seq: Single-cell RNA sequencing
PAR-1: Protease-activated receptor 1
PAR-2: Protease-activated receptor 2
SIRS: Systemic inflammatory response syndrome
TCA cycle: Tricarboxylic acid cycle
ER stress: Endoplasmic reticulum stress
tocilizumab: IL-6 receptor antagonist
GPRs: G-protein coupled receptors
CHARLS: China Health and Retirement Longitudinal Study
BioHEART-CT: A clinical study cohort
JAK2V617F: A JAK2 gene mutation
SOCS2: Suppressor of cytokine signaling 2
Nrf-2/HO-1: Nuclear factor erythroid 2-related factor 2/Heme oxygenase-1
PCSK9: Proprotein convertase subtilisin/kexin type 9
ACE2: Angiotensin-converting enzyme 2
mtDNA: Mitochondrial DNA
LncRNA GAS5: Long non-coding RNA growth arrest-specific 5
miR: MicroRNA
ANRIL: Antisense non-coding RNA in the INK4 locus
galectin-1/3: Galectin family proteins
CTRP3: C1q/tumor necrosis factor-related protein 3
OPG: Osteoprotegerin
p130Cas/BCAR1: p130 Crk-associated substrate/Breast cancer anti-estrogen resistance 1
TIPE2: Tumor necrosis factor alpha-induced protein 8-like 2
Pyroptosis: A form of programmed cell death
HDL: High-density lipoprotein
LDL: Low-density lipoprotein
BMI: Body mass index
NYHA: New York Heart Association.
Immunecomponent: Key mediators/cellsMonocytes/Macrophages (M1), IL-1β, IL-6, TNF-α, ROS
Macrophages (M2): IL-10, TGF-β
Neutrophils: NETs, MPO, elastase
T helper cells(Th1/Th17): IFN-γ, IL-17
RegulatoryT cells: IL-10, TGF-β
Platelets: P-selectin, TXA2, CD40L
Innate immunecells(with memory): oxLDL, free cholesterol
CHIP-associatedstem cells: TET2/DNMT3A mutations.
Factor: Immune-metabolic effectWestern diet, Trained pro-inflammatory immunity
High sugar intake: Insulin resistance
Fasting/time-restricted eating: Metabolic switching
Dietary fiber/SCFAs: Anti-inflammatory signaling
Exercise: Improved mitochondrial function
Psychological stress: Neuroendocrine activation
SCFA derivatives(4-PBA): Innate monocyte rejuvenation, erasure of inflammatory memory.
Exposure: Immune mechanismPM2.5 air pollution, Oxidative stress, macrophage activation
Heavy metals (Pb: Cd, Hg), ROS generation
Smoking/nicotine: M1 polarization, platelet activation
Alcohol (chronic): Immune dysregulation
Persistent organic pollutants: AhR activation
Cocaine: Platelet hyperactivation
Immune checkpoint inhibitors(ICIs): T-cell overactivation(T-cell infiltration)
CAR-T therapy: Innate immune cell dysregulation, massive cytokine release(CRS).
Biomarker: DefinitionNLR, Neutrophil/Lymphocyte ratio
SIRI: Neutrophil×Monocyte/Lymphocyte
hs-CRP: C-reactive protein
IL-6: Cytokine level
NET markers (cfDNA: MPO-DNA), NETosis
Multi-omics panels: Genomic/proteomic/metabolomic
