# Correction: Immuno-inflammatory-metabolic interactions in cardiovascular diseases: a review from basic mechanisms to clinical translation

**DOI:** 10.3389/fimmu.2026.1924972

**Published:** 2026-09-16

**Authors:** Xingshun Zhu, Fengmei Zhang, Yuxin Wei, Yan Zhao, Jiawei Guo

**Affiliations:** 1 Department of Vascular and Endovascular Surgery, The First Affiliated Hospital of Yangtze University, Jingzhou, China; 2 Department of Pharmacology, School of Medicine, Yangtze University, Jingzhou, China

**Keywords:** Allostatic load, cardiovascular disease, immune inflammation, lifestyle intervention, metabolic reprogramming, thrombo-inflammation, trained immunity

## Missing citation

The references for [1-2,10,12,15,17-20,22-71,73-82,84-85,87-130,132-215] were erroneously written as [1. Wang Q, Sui YX, Zhang LT, Meng N, Chen HY. Systemic review of inflammatory pathways and immune modulation in atherosclerotic cardiovascular disease. Am J Cardiol. (2026) 258:134–142. doi:10.1016/j.amjcard.2025.08.013

2. Misaka T, Kimishima Y, Yokokawa T, Ikeda K, Takeishi Y. Clonal hematopoiesis and cardiovascular diseases: role of JAK2V617F. J Cardiol. (2023) 81:3–9. doi:10.1016/j.jjcc.2022.02.001

10. Ziegler KA, Engelhardt S, Carnevale D, McAlpine CS, Guzik TJ, Dimmeler S, et al. Neural mechanisms in cardiovascular health and disease. Circ Res. (2025) 136:1233–1261. doi:10.1161/CIRCRESAHA.125.325580

12. Yvan-Charvet L, Barouillet T, Borowczyk C. Haematometabolism rewiring in atherosclerotic cardiovascular disease. Nat Rev Cardiol. (2025) 22:414–430. doi:10.1038/s41569-024-01108-9

15: Miller GE, Passarelli V, Chen E, Kloog I, Wright RJ, Amini H. Ambient PM2.5 and specific sources increase inflammatory cytokine responses to stimulators and reduce sensitivity to inhibitors. *Environ Res*. (2024) 252(Pt 2):118964. doi: 10.1016/j.envres.2024.118964

17. Nakayama Y, Fujiu K. Innate immune memory in macrophage differentiation and cardiovascular diseases. Inflamm Regen. (2025) 45:17. doi:10.1186/s41232-025-00382-5

18: Zheng J, Yi Y, Tian T, Luo S, Liang X, Bai Y. ICI-induced cardiovascular toxicity: mechanisms and immune reprogramming therapeutic strategies. Front Immunol. (2025) 16:1550400. doi:10.3389/fimmu.2025.1550400

19: Geng S, Chen K, Yuan R, Peng L, Maitra U, Diao N, et al. The persistence of low-grade inflammatory monocytes contributes to aggravated atherosclerosis. Nat Commun. (2016) 7:13436. doi:10.1038/ncomms13436

20: Hata M, Andriessen EMMA, Diaz-Marin R, Fournier F, Crespo-Garcia S, Blot G, et al. Past history of obesity triggers persistent epigenetic changes in innate immunity and exacerbates neuroinflammation. Science. (2023) 379(6627):45–62. doi:10.1126/science.abj8894

22. Geng S, Lu R, Zhang Y, Wu Y, Xie L, Caldwell BA, et al. Monocytes reprogrammed by 4- PBA potently contribute to the resolution of inflammation and atherosclerosis. Circ Res.(2024) 135(8):856–872.doi:10.1161/CIRCRESAHA.124.325023

23: Llena-Meler M, Canfran-Duque A, Madrigal-Matute J, Rotllan N. Lipid metabolic alterations in cancer: common pathophysiology with cardiovascular disease. Semin Cancer Biol. (2026) 119:83–105. doi:10.1016/j.semcancer.2026.01.003

24: Lee WE, Henry A, Spenceley ER, Sagi-Zsigmond E, Bowen B, Nguyen TV, et al. Increased mROS generation associates with cardiovascular risk in BioHEART-CT PBMCs. Clin Transl Sci. (2026) 19:e70469. doi:10.1111/cts.70469

25: Xu S, Zhang G, Xu Y, Yu C, Wu R, Li Z, et al. Allostatic load–cardiovascular disease associations and the mediating effect of inflammatory factors: a prospective cohort study. Front Cardiovasc Med. (2026) 12:1724572. doi:10.3389/fcvm.2025.1724572

26: Alhawamdeh N, Bani Hani DA, Alshraideh JA, Yousef AS, Saleh A. Novel inflammatory markers predict cardiovascular events after percutaneous coronary intervention in patients with acute coronary syndrome. Heart Lung. (2026) 75:111–116. doi:10.1016/j.hrtlng.2025.09.014

27: Andersson SHA, Chalou A, Mulholland M, Katra P, Ljungcrantz I, Andersson L, et al. Impaired SARS-CoV-2 vaccine responsiveness is not associated with subclinical atherosclerosis or cardiovascular disease. Eur Heart J Open. (2025) 6:oeaf167. doi:10.1093/ehjopen/oeaf167

28: Countouris ME, Bello NA. Advances in our understanding of cardiovascular diseases after preeclampsia. Circ Res. (2025) 136:583–593. doi:10.1161/CIRCRESAHA.124.325581

29: Kondapalli N, Katari V, Dalal KK, Paruchuri S, Thodeti CK. Microbiota in gut-heart axis: metabolites and mechanisms in cardiovascular disease. Compr Physiol. (2025) 15:e70024. doi:10.1002/cph4.70024

30: Liao Y, Niu L, Ling J, Cui Y, Huang Z, Xu J, et al. Turning sour into sweet: lactylation modification as a promising target in cardiovascular health. Metabolism. (2025) 168:156234. doi:10.1016/j.metabol.2025.156234

31: Itzhaki Ben Zadok O, O’Hare MJ, Nohria A. Immune checkpoint inhibitor-related myocarditis with or without concomitant myopathy: clinical findings and cardiovascular outcomes. JACC CardioOncol. (2025) 7:252–264. doi:10.1016/j.jaccao.2025.02.005

32: Amin A, Saadatakhtar M, Mohajerian A, Marashi SM, Zamanifard S, Keshavarzian A, et al. Mercury-mediated cardiovascular toxicity: mechanisms and remedies. Cardiovasc Toxicol. (2025) 25:507–522. doi:10.1007/s12012-025-09966-6

33: Atencio JK, Reed EL, Wiedenfeld Needham K, Lucernoni KM, Comrada LN, Halliwill JR, et al. Comparison of thermoregulatory, cardiovascular, and immune responses to different passive heat therapy modalities. Am J Physiol Regul Integr Comp Physiol. (2025) 329:R20–R35. doi:10.1152/ajpregu.00012.2025

34: Cappucci IP, Tremoli E, Zavan B, Ferroni L. Circulating extracellular vesicles in cardiovascular disease. Int J Mol Sci. (2025) 26:6817. doi:10.3390/ijms26146817

35: McClements L, Kautzky-Willer A, Kararigas G, Ahmed SB, Stallone JN. The role of sex differences in cardiovascular, metabolic, and immune functions in health and disease: a review for “Sex Differences in Health Awareness Day”. Biol Sex Differ. (2025) 16:33. doi:10.1186/s13293-025-00714-7

36: Wang Z, Dang Y, Li Y, Zhang Y, Zhou S, Zhang Z, et al. Recent advances in the interaction of ferroptosis and immune-mediated inflammation in cardiovascular disease: mechanisms and therapeutic potential. Front Immunol. (2025) 16:1691705. doi:10.3389/fimmu.2025.1691705

37: Song J, Chi KY, Jeon H, Chang YC, Xanthavanij N, Tang Z, et al. Statin and immune-related cardiovascular events in lung cancer patients receiving immune checkpoint inhibitors. Oncology. (2025). doi:10.1159/000546204

38: Cao Q, Liu J, Lu D, Song J, Yu Q, Wen J, et al. Dissecting the connection between volatile organic compounds and cardiovascular disease: immune cell-mediated mechanism. Ecotoxicol Environ Saf. (2025) 298:118297. doi:10.1016/j.ecoenv.2025.118297

39: Ahmad SMS, Ahmadi FAM, Giordo R, Casu G, Nasrallaha GK, Zayed H, et al. Molecular insights and emerging therapeutic perspectives of the lncRNA GAS5/miR-21 axis in cancer, fibrosis, cardiovascular, and immune disorders. Mutat Res Rev Mutat Res. (2025) 796:108551. doi:10.1016/j.mrrev.2025.108551

40: Grieco T, Paolino G, Moliterni E, Chello C, Sernicola A, Brandi ML, et al. Non-skeletal roles of vitamin D in skin, gut, and cardiovascular disease: focus on epithelial barrier function and immune regulation in chronic disease. Int J Mol Sci. (2025) 26:8520. doi:10.3390/ijms26178520

41: Song M, Liu B, Wang H, Sun W. Lactate metabolism and lactylation modification: new opportunities and challenges in cardiovascular disease. MedComm (2020). (2025) 6:e70269. doi:10.1002/mco2.70269

42: Hang L, Zhang Y, Zhang Z, Jiang H, Xia L. Metabolism serves as a bridge between cardiomyocytes and immune cells in cardiovascular diseases. Cardiovasc Drugs Ther. (2025) 39:661–676. doi:10.1007/s10557-024-07545-5

43: Lee H, Hong T, Park J, Kweon J, Kim S, Song G, et al. Methomyl-induced developmental and cardiovascular toxicity in zebrafish via immune response, oxidative stress, and apoptosis. Sci Total Environ. (2025) 997:180215. doi:10.1016/j.scitotenv.2025.180215

44: Tardo DT, Cortes-Canteli M, Fuster V, Sachdev PS, Kovacic JC. The heart-brain-metabolism axis in cardiovascular and neurologic disease. J Am Coll Cardiol. (2025) 86:2663–2686. doi:10.1016/j.jacc.2025.09.1602

45: Jing D, Xiong L, Zhang R, Fang H, Chen L. Esmolol improves sepsis outcomes through cardiovascular and immune modulation. Front Pharmacol. (2025) 16:1498227. doi:10.3389/fphar.2025.1498227

46. Karakasis P, Theofilis P, Vlachakis PK, Grigoriou K, Patoulias D, Antoniadis AP, et al. Reprogramming atherosclerosis: precision drug delivery, nanomedicine, and immune-targeted therapies for cardiovascular risk reduction. Pharmaceutics. (2025) 17:1028. doi:10.3390/pharmaceutics17081028

47. Han J, Bloxham CJ, Kirmes K, Viggiani G, Unkelbach LP, von Scheidt M, et al. Platelet- leukocyte aggregates in cardiovascular disease: prognostic significance and therapeuticpotential. Cardiovasc Res. (2025) 121:1679–1696.doi:10.1093/cvr/cvaf105

48: Ma L, Yang W, Gao W, Liu X, Dong M, An G, et al. IL-17 as a therapeutic target in cardiovascular diseases: mechanistic insights and translational opportunities. Pharmacol Res. (2025) 219:107879. doi:10.1016/j.phrs.2025.107879

49: Qian X, Sun J, Li F, Xu L, Hu X, Dong N, et al. Inflammasomes as the molecular hub of cardiovascular-metabolic-immune comorbidity networks. Front Immunol. (2025) 16:1690443. doi:10.3389/fimmu.2025.1690443

50: Atlante S, Gottardi Zamperla M, Cis L, Farsetti A, Gaetano C. Senolytic therapies for cardiovascular aging: tackling fibrosis and metabolic dysfunction. Eur J Intern Med. (2025) 140:106413. doi:10.1016/j.ejim.2025.07.009

51: Du H, Wang J, Wang Z. Cardiovascular adverse effects of immunotherapy in cancer: insights and implications. Front Oncol. (2025) 15:1601808. doi:10.3389/fonc.2025.1601808

52: Aljabali AAA, Obeid MA, Gammoh O, Alshaer W, Qnais E, Alqudah A, et al. Cathepsin C: a critical mediator between immune response and cardiovascular disease—therapeutic implications of enzyme inhibition. Drug Dev Ind Pharm. (2025). doi:10.1080/03639045.2025.2571722

53: Kwak H, Lee E, Karki R. DNA sensors in metabolic and cardiovascular diseases: molecular mechanisms and therapeutic prospects. Immunol Rev. (2025) 329:e13382. doi:10.1111/imr.13382

54: Kulkarni H, Gaikwad AB. The mitochondria-gut microbiota crosstalk—a novel frontier in cardiovascular diseases. Eur J Pharmacol. (2025) 998:177562. doi:10.1016/j.ejphar.2025.177562

55: Alsiraj Y, Huang H, Shoemaker R, Schanbacher B, Murphy M, Giannone P, Bauer JA. Maternal nutritional programming: sex-specific cardiovascular and immune outcomes following perinatal high-fat diet exposure. Nutrients. (2025) 17:1464. doi:10.3390/nu17091464

56: Edpuganti S, Subhash S, Subrahmaniyan SL, Latheef S, Albarari SS. Gut microbiome and cardiovascular health: mechanisms, therapeutic potential and future directions. Heart Int. (2025) 19:12–20. doi:10.17925/HI.2025.19.2.3

57: Li R, Guo Z. TIPE2: a novel regulatory factor for cardiovascular-related diseases. J Mol Med (Berl). (2025) 103:913–921. doi:10.1007/s00109-025-02564-7

58: Vasquez F, Tiscornia C, Lorca-Ponce E, Aicardi V, Vasquez S. Cardiorenal syndrome: molecular pathways linking cardiovascular dysfunction and chronic kidney disease progression. Int J Mol Sci. (2025) 26:7440. doi:10.3390/ijms26157440

59: Wei X, Zhang X, Cui M, Huang L, Gao D, Hua S. An exploration of the relationship between gut virome and cardiovascular disease: a comprehensive review. Rev Cardiovasc Med. (2025) 26:36386. doi:10.31083/RCM36386

60: Mohsenzadeh A, Pourasgar S, Mohammadi A, Nazari M, Nematollahi S, Karimi Y, et al. The gut microbiota and cardiovascular disease: exploring the role of microbial dysbiosis and metabolites in pathogenesis and therapeutics. Life Sci. (2025) 381:123981. doi:10.1016/j.lfs.2025.123981

61: Chen Z, Zhang Y, Qian C, Xia L. The role of the pentose phosphate pathway in cardiovascular diseases. Cardiovasc Drugs Ther. (2025). doi:10.1007/s10557-025-07788-w

62: Biondi-Zoccai G, Weber BN, Abbate A, Booz GW. Cardiac immunotherapy, immuno-cardiology, and the future of cardiovascular pharmacology. J Cardiovasc Pharmacol. (2025) 85:308–311. doi:10.1097/FJC.0000000000001687

63: Linsley VG, Bishop NC, Roberts MJ, Hamrouni M, Demashkieh M, Paine NJ. Inflammatory and cardiovascular responses to active and passive acute psychological stress. Biopsychosoc Sci Med. (2025) 87:107–117. doi:10.1097/PSY.0000000000001367

64: Sathik N, Safadi R, Saini I, Ahuja A, Hu J, Raina R. Cardiovascular assessments in children and adolescents with hypertension. Rev Cardiovasc Med. (2025) 26:39498. doi:10.31083/RCM39498

65: Xu Q, Liu X, Wang Z, Li X, Jiang Q, Xu M. Recent advancements and comprehensive analyses of butyric acid in cardiovascular diseases. Front Cardiovasc Med. (2025) 12:1608658. doi:10.3389/fcvm.2025.1608658

66: Tabatabaei FS, Shafeghat M, Azimi A, Akrami A, Rezaei N. Endosomal toll-like receptors intermediate negative impacts of viral diseases, autoimmune diseases, and inflammatory immune responses on the cardiovascular system. Expert Rev Clin Immunol. (2025) 21:195–207. doi:10.1080/1744666X.2024.2392815

67: Abarca YA, Chadalavada B, Ceron JR, Sai BA, Bhatia A, Espinoza I, et al. A comprehensive review of the manifestation of cardiovascular diseases in HIV patients. Cureus. (2025) 17:e77509. doi:10.7759/cureus.77509

68: Lu Y, Wei M, Zhang K, Zhang P, Liu D, Jiang Z, et al. Systemic immune-inflammation index as a predictor of statin residual cardiovascular risk compared with C-reactive protein. Int J Cardiol. (2025) 438:133529. doi:10.1016/j.ijcard.2025.133529

69: Lu QB, Zhu XX, Chen G, Su JB, Zhao CY, Xu AJ, et al. Role of hydrogen sulfide regulation of programmed cell death: implications for cardiovascular diseases. Antioxid Redox Signal. (2025) 43:637–690. doi:10.1177/15230864251386449

70: Zhang H, Huang W, Bao S. Dichotomous roles of IL-36 and IL-38 in cardiovascular disease. Front Immunol. (2025) 16:1642116. doi:10.3389/fimmu.2025.1642116

71: Izquierdo-Condoy JS, Arias-Intriago M, Becerra Cardona DA, García-Cañarte S, Vinueza-Moreano P. Anticancer chemotherapy-induced atherosclerotic cardiovascular disease: a comprehensive review. Life (Basel). (2025) 15:245. doi:10.3390/life15020245

73: Bueno G, Bot I, Kanhai AA, Stilhano R, Libby P, De Meyer GRY, Martin L. Mast cells in cardiovascular disease: fibrosis, angiogenesis and atherogenesis. Prog Cardiovasc Dis. (2025). doi:10.1016/j.pcad.2025.11.007

74: Li X, Yan Z, Lan H, Wu Y, Chen S, Qiu G, et al. Genetic comorbidity of psoriasis and four cardiovascular diseases: uncovering shared mechanisms and potential therapeutic targets. Exp Dermatol. (2025) 34:e70158. doi:10.1111/exd.70158

75: He JM, Yang Y. Association between neutrophil-lymphocyte ratio and all-cause and cardiovascular mortality in patients with diabetes or prediabetes with comorbid obstructive sleep apnea symptoms: evidence from NHANES 2005–2008 and 2015–2018. Front Endocrinol (Lausanne). (2025) 16:1512621. doi:10.3389/fendo.2025.1512621

76: Liu Y, Zhang F, Liu R, Li J, Chen C, Huang X, et al. Prognostic nutritional index predicts staging and mortality in cardiovascular-kidney-metabolic syndrome. PLoS One. (2025) 20:e0338084. doi:10.1371/journal.pone.0338084

77: Nidadavolu LS, Sosnowski DW, Sivakumar N, Merino Gomez A, Wu Y, Laskow T, et al. Cardiovascular-derived circulating cell-free DNA fragments are associated with frailty and increased cardiovascular events in older adults. J Gerontol A Biol Sci Med Sci. (2025) 80:glaf081. doi:10.1093/gerona/glaf081

78: Lu H, Jiang Y, Luo R, Zhou D, Zheng F, Shi L, et al. Engineered hybrid cell membrane nanosystems for treating cardiovascular diseases. Mater Today Bio. (2025) 33:101992. doi:10.1016/j.mtbio.2025.101992

79: Al Noman A, Flora SA, Datta M, Afrose F, Binte Hasan N, Akhter T, et al. Exploring the involvement of new members of the interleukin family in cardiovascular disease. Curr Cardiol Rev. (2025) 21:e1573403X330079. doi:10.2174/011573403X330079241213071055

80: Pervin M, de Haan JB. Dysregulated redox signaling and its impact on inflammatory pathways, mitochondrial dysfunction, autophagy and cardiovascular diseases. Antioxidants (Basel). (2025) 14:1278. doi:10.3390/antiox14111278

81: Khafaji ATA, Barakat AM, Zaidy AJA, Taan AA, Al-Aouadi RFA. Exosomes in cardiovascular disease: emerging roles, therapeutic potential and translational challenges. Basic Clin Pharmacol Toxicol. (2025) 137:e70151. doi:10.1111/bcpt.70151

82: Abi Karam K, Abohashem S, Lau HC, Civieri G, Aldosoky W, Khalil M, et al. Stress-related genetic factors modify the effect of socioeconomic status on cardiovascular risk.

Neuropsychopharmacology. (2025) 50:1327–1335. doi:10.1038/s41386-025-02130-2

84: Bittner N, Korf HW, Moebus S, Schmidt B, Caspers S. Association between night shift work and markers of metabolism, cardiovascular and immune system in a population-based German cohort. Geroscience. (2025) 47:5141–5155. doi:10.1007/s11357-025-01596-8

85: Wu F, Yang J, Liu Y, Zhang Y. Extremely high systemic immune inflammation levels increase the risk of all-cause and cardiovascular mortality in postmenopausal women. Int J Womens Health. (2025) 17:1457–1468. doi:10.2147/IJWH.S504664

87: Tian S, Li Z, Bi Y, Che X, Feng A, Wang Y. Identifying pathways to cardiovascular mortality by causal graphical models and mediation analysis among hypertensive patients: insights from a prospective study. J Transl Med. (2025) 23:690. doi:10.1186/s12967-025-06755-1

88: Chiang CH, Song J, Chi KY, Chang YC, Xanthavanij N, Chang Y, et al. Glucagon-like peptide-1 agonists reduce cardiovascular events in cancer patients on immune checkpoint inhibitors. Eur J Cancer. (2025) 216:115170. doi:10.1016/j.ejca.2024.115170

89: Theobald D, de Castro Braz LE, Akula SM, Eells JB, Sriramula S. Inhibition of kinin B1 receptor alleviates SARS-CoV-2-induced long-lasting cardiovascular complications. Am J Physiol Heart Circ Physiol. (2025) 328:H711–H718. doi:10.1152/ajpheart.00861.2024

90: K A, Choraria G, Basha S, Mahato KK. Emerging insights of fibrinogen glycation in cardiovascular disorders: a review. Int J Biol Macromol. (2025) 331:148461. doi:10.1016/j.ijbiomac.2025.148461

91: Kilkenny K, Frishman W. Preeclampsia’s cardiovascular aftermath: a comprehensive review of consequences for mother and offspring. Cardiol Rev. (2025) 33:448–454. doi:10.1097/CRD.0000000000000639

92: Awais M, Baroudi M, Mankani MH, Ali A, Khokhar ME, Shaukat N, et al. Therapeutic immunomodulation in cardiovascular disease: anti-inflammatory strategies and landmark trials. Cureus. (2025) 17:e99835. doi:10.7759/cureus.99835

93: Mohammadian K, Asayesh M, Fakhar F, Keramat S, Stanek A. The interplay between autoimmune disorders affecting the coagulation and platelet systems and their implications for cardiovascular diseases: a systematic review. Cells. (2025) 14:1023. doi:10.3390/cells14131023

94: Lakhanpal S, Aggarwal K, Kaur H, Kanwar K, Gupta V, Bhavsar J, Jain R. Cardiovascular disease: extraintestinal manifestation of inflammatory bowel disease. Intest Res. (2025) 23:23–

36. doi:10.5217/ir.2023.00104

95: Zhou P, Jiang X, Wang W, Wang D. Dissection of shared genetic architecture and biological association between psoriasis and cardiovascular disease based on genome-wide association studies. Hum Immunol. (2025) 86:111565. doi:10.1016/j.humimm.2025.111565

96: U W, Hong L, Ramteke HD, Yang J, He Z. Systematic review and meta-analysis of cardiovascular associated biomarkers in adults with asymptomatic autoimmune diseases. Front Cardiovasc Med. (2025) 12:1598590. doi:10.3389/fcvm.2025.1598590

97: Feng M, Meng F, Jia Y, Wang Y, Ji G, Gao C, Luo J. Exploration of risk factors for cardiovascular disease in patients with rheumatoid arthritis: a retrospective study. Inflammation. (2025) 48:1811–1827. doi:10.1007/s10753-024-02157-5

98: Chen PY, Cheng CY, Chen CC, Chen HY, Liu JC, Hao WR, et al. Galectin-1 in cardiovascular pathogenesis: unraveling dual roles and mechanistic insights in emerging research. Medicina (Kaunas). (2025) 61:1020. doi:10.3390/medicina61061020

99: Zhao X, Chen W, Wu J, Shen Y, Xu B, Chen Z, Sun Y. Application of biomimetic cell membrane-coated nanocarriers in cardiovascular diseases. Int J Nanomedicine. (2025) 20:8249–8289. doi:10.2147/IJN.S531558

100: Tian Y, Wang P, Dong Z. Heart-guarding or heart-harming? The dual role of epicardial adipose tissue in cardiovascular health and disease. Int J Obes (Lond). (2025) 49:2156–2167. doi:10.1038/s41366-025-01852-z

101: Dui W, Xiaobin Z, Haifeng Z, Lijuan D, Wenhui H, Zhengfeng Z, Jinling S. Harnessing RNA therapeutics: novel approaches and emerging strategies for cardiovascular disease management. Front Cardiovasc Med. (2025) 12:1546515. doi:10.3389/fcvm.2025.1546515

102: Liu XS, Li ZY, Wang ST, Qiao Q, Ye XR, Cheng L, et al. Pyroptosis in cardiovascular diseases: molecular mechanisms, pathological roles, and therapeutic implications. Am J Cardiovasc Dis. (2025) 15:477–508. doi:10.62347/MTTP3167

103: Zhang W, Jiang G, Liu Z, Duan L, Liang J, Wang Z, et al. Cobalt mediates stage-specific toxicity of metal mixtures in cardiovascular-kidney-metabolic syndrome. Toxicol Sci. (2025). doi:10.1093/toxsci/kfaf172

104: Ratra S, Mukherjee S, Ray S, Yu A, Rengan AK. Inventive HDL mimicking nanoparticles: a promising frontier in cardiovascular disease management. Cardiovasc Toxicol. (2025) 26:2. doi:10.1007/s12012-025-10074-8

105: Zhang S, Han M, Li S, Wang Y, Wu L, Dong L, Liang P. Platelet-derived biomimetic nanoplatforms: a promising drug delivery strategy for cardiovascular disease. J Nanobiotechnology. (2025) 23:687. doi:10.1186/s12951-025-03700-8

106. Jimenez-Gallegos D, Sigcho F, Vicencio E, Arias-Carrasco R, Lavandero S, Parra V, et al. The multifaceted roles of non-coding RNAs in cell-cell communication in cardiovascular health and disease. Cell Biosci. (2025) 15:154. doi:10.1186/s13578-025-01484-w

107: Tumenbayar BI, Pham K, Biber JC, Drewes R, Bae Y. Transcriptomic and multi-scale network analyses reveal key drivers of cardiovascular disease. IEEE Trans Mol Biol Multiscale Commun. (2025) 11:78–90. doi:10.1109/tmbmc.2024.3501576

108: Yuan HH, Yin H, Marincas M, Xie LL, Bu LL, Guo MH, Zheng XL. From DNA repair to redox signaling: the multifaceted role of APEX1 in cardiovascular health and disease. Int J Mol Sci. (2025) 26:3034. doi:10.3390/ijms26073034

109: Martuszewski A, Paluszkiewicz P, Poręba R, Gać P. Galectin-3 in cardiovascular health: a narrative review based on Life’s Essential 8 and Life’s Simple 7 frameworks. Curr Issues Mol Biol. (2025) 47:332. doi:10.3390/cimb47050332

110: Bai Y, Bai Y, Wu Z, Chen Q, Jiang N. Selection of common genes associated with rheumatoid arthritis and cardiovascular disease via a network- and pathway-based approach. Anatol J Cardiol. (2025) 29:701–714. doi:10.14744/AnatolJCardiol.2025.5375

111: Fiste O, Samiotaki M, Manios E, Trika C, Liacos CI, Dimitrakakis C, et al. Serum proteomics of ribociclib-mediated cardiovascular toxicity: an exploratory case-control study. Proteomics Clin Appl. (2025) 19:e70021. doi:10.1002/prca.70021

112. Baek JH, Grewal SS, Karam KA, Hanlon ER, Abohashem S, Seligowski AV, et al. Neurobiological mechanisms link bipolar disorder to cardiovascular disease: aretrospective biobank study of adverse event risk and contributory mechanisms. Bipolar Disord. (2025) 27:57–66.doi:10.1111/bdi.13516

113.Suleimenova M, Abzaliyev K, Mansurova M, Abzaliyeva S, Kurmanova A,Tokhtakulinova G, et al. A predictive model of cardiovascular aging by clinical and immunological markers using machine learning. Diagnostics (Basel). (2025) 15:850.doi:10.3390/diagnostics15070850

114: Aburel OM, Brăescu L, Buriman DG, Merce AP, Bînă AM, Borza C, et al. Methylene blue reduces monoamine oxidase expression and oxidative stress in human cardiovascular adipose tissue. Mol Cell Biochem. (2025) 480:2413–2421. doi:10.1007/s11010-024-05092-z

115: Prattichizzo F, Ceriello A, Pellegrini V, La Grotta R, Graciotti L, Olivieri F, et al. Micro-nanoplastics and cardiovascular diseases: evidence and perspectives. Eur Heart J. (2024) 45:4099–4110. doi:10.1093/eurheartj/ehae552

116: Hmiel L, Zhang S, Obare LM, Santana MAO, Wanjalla CN, Titanji BK, et al. Inflammatory and immune mechanisms for atherosclerotic cardiovascular disease in HIV. Int J Mol Sci. (2024) 25:7266. doi:10.3390/ijms25137266

117: Keith RJ, Hart JL, Bhatnagar A. Greenspaces and cardiovascular health. Circ Res. (2024) 134:1179–1196. doi:10.1161/CIRCRESAHA.124.323583

118: Torimoto K, Elliott K, Nakayama Y, Yanagisawa H, Eguchi S. Cardiac and perivascular myofibroblasts, matrifibrocytes, and immune fibrocytes in hypertension; commonalities and differences with other cardiovascular diseases. Cardiovasc Res. (2024) 120:567–580. doi:10.1093/cvr/cvae044

119: Zarate SM, Kirabo A, Hinton AO Jr, Santisteban MM. Neuroimmunology of cardiovascular disease. Curr Hypertens Rep. (2024) 26:339–347. doi:10.1007/s11906-024-01301-8

120: Abulizi A, Yan G, Xu Q, Muhetaer R, Wu S, Abudukelimu K, et al. Cardiovascular adverse events and immune-related adverse events associated with PD-1/PD-L1 inhibitors for head and neck squamous cell carcinoma. Sci Rep. (2024) 14:25919. doi:10.1038/s41598-024-75099-5

121: Xia Y, Gao D, Wang X, Liu B, Shan X, Sun Y, Ma D. Role of Treg cell subsets in cardiovascular disease pathogenesis and potential therapeutic targets. Front Immunol. (2024) 15:1331609. doi:10.3389/fimmu.2024.1331609

122: Kreidieh F, McQuade J. Novel insights into cardiovascular toxicity of cancer targeted and immune therapies: beyond ischemia with non-obstructive coronary arteries (INOCA). Am Heart J Plus. (2024) 40:100374. doi:10.1016/j.ahjo.2024.100374

123: Yin X, Zhang Y, Zou J, Yang J. Association of the systemic immune-inflammation index with all-cause and cardiovascular mortality in individuals with rheumatoid arthritis. Sci Rep. (2024) 14:15129. doi:10.1038/s41598-024-66152-4

124: Jiang R, Lou L, Shi W, Chen Y, Fu Z, Liu S, et al. Statins in mitigating anticancer treatment-related cardiovascular disease. Int J Mol Sci. (2024) 25:10177. doi:10.3390/ijms251810177

125: Dare A, Chen SY. Adipsin in the pathogenesis of cardiovascular diseases. Vascul Pharmacol. (2024) 154:107270. doi:10.1016/j.vph.2023.107270

126: Alradwan I, Al Fayez N, Alomary MN, Alshehri AA, Aodah AH, Almughem FA, et al. Emerging trends and innovations in the treatment and diagnosis of atherosclerosis and cardiovascular disease: a comprehensive review towards healthier aging. Pharmaceutics. (2024) 16:1037. doi:10.3390/pharmaceutics16081037

127: Li Q, Zhou Z, Xu T, Gao X, Lou Y, Chen Z, et al. Relationship between cathepsins and cardiovascular diseases: a Mendelian randomized study. Front Pharmacol. (2024) 15:1370350. doi:10.3389/fphar.2024.1370350

128: Chen C, Zhong W, Zheng H, Dai G, Zhao W, Wang Y, et al. The role of uromodulin in cardiovascular disease: a review. Front Cardiovasc Med. (2024) 11:1417593. doi:10.3389/fcvm.2024.1417593

129: Fujiu K. Brain-heart dialogue – decoding its role in homeostasis and cardiovascular disease. Circ J. (2024) 88:1354–1359. doi:10.1253/circj.CJ-23-0579

130. Hage Z, Madeira MM, Koliatsis D, Tsirka SE. Convergence of endothelial dysfunction, inflammation and glucocorticoid resistance in depression-related cardiovascular diseases. BMC Immunol. (2024) 25:61. doi:10.1186/s12865-024-00653-9

132: Shrivastav A, Swetanshu, Singh P. The impact of environmental toxins on cardiovascular diseases. Curr Probl Cardiol. (2024) 49:102120. doi:10.1016/j.cpcardiol.2023.102120

133: Yao J, Chen Y, Huang Y, Sun X, Shi X. The role of cardiac microenvironment in cardiovascular diseases: implications for therapy. Hum Cell. (2024) 37:607–624. doi:10.1007/s13577-024-01052-3

134: Zhu G, Cao L, Wu J, Xu M, Zhang Y, Wu M, Li J. Co-morbid intersections of cancer and cardiovascular disease and targets for natural drug action: reprogramming of lipid metabolism. Biomed Pharmacother. (2024) 176:116875. doi:10.1016/j.biopha.2024.116875

135: Wang K, Liu CY, Fang B, Li B, Li YH, Xia QQ, et al. The function and therapeutic potential of transfer RNA-derived small RNAs in cardiovascular diseases: a review. Pharmacol Res. (2024) 206:107279. doi:10.1016/j.phrs.2024.107279

136. Tan H, Zhou H, Chen J, Ren H, Guo Y, Jiang X. Association of early life adversity with cardiovascular disease and its potential mechanisms: a narrative review. Front Public Health. (2024) 12:1341266. doi:10.3389/fpubh.2024.1341266

137: Ysphaneendramallimoggala, Biswas M, Anburaj SE, Iqbal F, A S, Suryakanth VB, Lewis LES. Thiamine: an indispensable regulator of paediatric neuro-cardiovascular health and diseases. Eur J Pediatr. (2024) 183:4597–4610. doi:10.1007/s00431-024-05756-4

138: Chen Z, Li W, Tang Y, Zhou P, He Q, Deng Z. The neutrophil-lymphocyte ratio predicts all-cause and cardiovascular mortality among United States adults with COPD: results from NHANES 1999–2018. Front Med (Lausanne). (2024) 11:1443749. doi:10.3389/fmed.2024.1443749

139: Wang X, Sun H, Yu H, Du B, Fan Q, Jia B, Zhang Z. Bone morphogenetic protein 10, a rising star in the field of diabetes and cardiovascular disease. J Cell Mol Med. (2024) 28:e18324. doi:10.1111/jcmm.18324

140: Haines DD, Cowan FM, Tosaki A. Evolving strategies for use of phytochemicals in prevention and long-term management of cardiovascular diseases. Int J Mol Sci. (2024) 25:6176. doi:10.3390/ijms25116176

141: Lemini C, Silveyra P, Segovia-Mendoza M. Cardiovascular disrupting effects of bisphenols, phthalates, and parabens related to endothelial dysfunction: review of toxicological and pharmacological mechanisms. Environ Toxicol Pharmacol. (2024) 107:104407. doi:10.1016/j.etap.2024.104407

142: Ali AE, Becker RC. The foundation for investigating factor XI as a target for inhibition in human cardiovascular disease. J Thromb Thrombolysis. (2024) 57:1283–1296. doi:10.1007/s11239-024-02985-0

143: van der Valk ES, Mohseni M, Iyer AM, van den Hurk MJB, Lengton R, Kuckuck S, et al. Long-term glucocorticoid exposure and incident cardiovascular diseases – the Lifelines cohort. J Clin Endocrinol Metab. (2024) 109:2520–2529. doi:10.1210/clinem/dgae081

144: Schmid A, Pankuweit S, Vlacil AK, Koch S, Berge B, Gajawada P, et al. Decreased circulating CTRP3 levels in acute and chronic cardiovascular patients. J Mol Med (Berl). (2024) 102:667–677. doi:10.1007/s00109-024-02426-8

145: Fox CB, Butler K, Flynn D. Primary prevention of cardiovascular disease for people living with human immunodeficiency virus. Nurs Clin North Am. (2024) 59:219–233. doi:10.1016/j.cnur.2023.12.001

146: Nandakumar M, Sathyapalan T, Atkin SL, Butler AE. Effect of hypoglycemia and rebound hyperglycemia on proteomic cardiovascular risk biomarkers. Biomedicines. (2024) 12:1137. doi:10.3390/biomedicines12061137

147: Attachaipanich T, Chattipakorn SC, Chattipakorn N. Current evidence regarding the cellular mechanisms associated with cancer progression due to cardiovascular diseases. J Transl Med. (2024) 22:105. doi:10.1186/s12967-023-04803-2

148: Fowler JWM, Song L, Tam K, Roth Flach RJ. Targeting lymphatic function in cardiovascular-kidney-metabolic syndrome: preclinical methods to analyze lymphatic function and therapeutic opportunities. Front Cardiovasc Med. (2024) 11:1412857. doi:10.3389/fcvm.2024.1412857

149: Luo D, Bai M, Zhang W, Wang J. The possible mechanism and research progress of ACE2 involved in cardiovascular injury caused by COVID-19: a review. Front Cardiovasc Med. (2024) 11:1409723. doi:10.3389/fcvm.2024.1409723

150: Vargas-Antillón AB, Porchas-Quijada M, Zepeda-Carrillo EA, Torres-Valadez R, Muñoz-Valle JF, Vázquez-Solórzano R, et al. Antibodies reactive to leptin in adults with type 2 diabetes and its relationship with clinical, metabolic and cardiovascular risk parameters. Endocr Res. (2024) 49:12–21. doi:10.1080/07435800.2023.2270763

151: Ghasempour Dabaghi G, Rabiee Rad M, Amani-Beni R, Darouei B. The role of p130Cas/BCAR1 adaptor protein in the pathogenesis of cardiovascular diseases: a literature review. Am Heart J Plus. (2024) 44:100416. doi:10.1016/j.ahjo.2024.100416

152: Zarenezhad E, Hadi AT, Nournia E, Rostamnia S, Ghasemian A. A comprehensive review on potential in silico screened herbal bioactive compounds and host targets in the cardiovascular disease therapy. Biomed Res Int. (2024) 2024:2023620. doi:10.1155/2024/2023620

153: Zhou J, Wei C, Li G, He W, Song M, Liu X, et al. The involvement of circulating miR-146a and miR-27a in patients with atherosclerotic cardiovascular disease after SARS-CoV-2 infection. Clin Cardiol. (2024) 47:e24274. doi:10.1002/clc.24274

154: Akhtar SMM, Ali A, Fareed A, Hasan J. Osteoprotegerin (OPG): a potential biomarker for adverse cardiovascular events in stable coronary artery disease. Health Sci Rep. (2024) 7:e2253. doi:10.1002/hsr2.2253

155: Stabellini N, Nettles D, Nain P, Moore JX, Weintraub NL, Patel SA, et al. The mediation role of allostatic load/chronic stress on the relationship between cancer survivorship and cardiovascular disease mortality. Am Heart J Plus. (2024) 45:100429. doi:10.1016/j.ahjo.2024.100429

156: Kobat H, Elkonaissi I, Foreman E, Davidson M, Idaikkadar P, O’Brien M, et al. Smoking, diabetes mellitus, and previous cardiovascular disease as predictors of anticancer treatment-induced cardiotoxicity in non-small-cell lung cancer: a real-world study. Clin Lung Cancer. (2024) 25:e35–e42. doi:10.1016/j.cllc.2023.09.007

157: Hasan ML, Lee JR, Rahaman KA, Yang DH, Joung YK. Versatile effects of galectin-1 protein-containing lipid bilayer coating for cardiovascular applications. Bioact Mater. (2024) 42:207–225. doi:10.1016/j.bioactmat.2024.08.026

158: Cui P, Li D, Shi L, Yan H, Li T, Liu C, et al. Cardiovascular comorbidities among patients with psoriasis: a national register-based study in China. Sci Rep. (2024) 14:19683. doi:10.1038/s41598-024-70707-w

159: Alotaiq N, Dermawan D, Elwali NE. Leveraging therapeutic proteins and peptides from Lumbricus earthworms: targeting SOCS2 E3 ligase for cardiovascular therapy through molecular dynamics simulations. Int J Mol Sci. (2024) 25:10818. doi:10.3390/ijms251910818

160: Lateef SS, Ward GA, Li H, Pantoja C, Florida E, Hong CG, et al. Circulating oxidized mtDNA is associated broadly with cardiovascular disease in a longitudinal cohort study of psoriasis. JID Innov. (2023) 4:100243. doi:10.1016/j.xjidi.2023.100243

161: Green CE, Chacon J, Godinich BM, Hock R, Kiesewetter M, Raynor M, et al. The heart of the matter: immune checkpoint inhibitors and immune-related adverse events on the cardiovascular system. Cancers (Basel). (2023) 15:5707. doi:10.3390/cancers15245707

162: Acharya S, Liao S, Jung WJ, Kang YS, Moghaddam VA, Feitosa MF, et al. A methodology for gene level omics-WAS integration identifies genes influencing traits associated with cardiovascular risks: the Long Life Family Study. Hum Genet. (2024) 143:1241–1252. doi:10.1007/s00439-024-02701-1

163. Singh V, Kaur R, Kumari P, Pasricha C, Singh R. ICAM-1 and VCAM-1: gatekeepers in various inflammatory and cardiovascular disorders. Clin Chim Acta. (2023) 548:117487. doi:10.1016/j.cca.2023.117487

164: Argano C, Mirarchi L, Amodeo S, Orlando V, Torres A, Corrao S. The role of vitamin D and its molecular bases in insulin resistance, diabetes, metabolic syndrome, and cardiovascular disease: state of the art. Int J Mol Sci. (2023) 24:15485. doi:10.3390/ijms242015485

165: Zhou E, Wu J, Zhou X, Yin Y. The neutrophil-lymphocyte ratio predicts all-cause and cardiovascular mortality among U.S. adults with rheumatoid arthritis: results from NHANES 1999–2020. Front Immunol. (2023) 14:1309835. doi:10.3389/fimmu.2023.1309835

166: Liu H, Nguyen HH, Hwang SY, Lee SS. Oxidative mechanisms and cardiovascular abnormalities of cirrhosis and portal hypertension. Int J Mol Sci. (2023) 24:16805. doi:10.3390/ijms242316805

167: Yegya-Raman N, Berlin E, Feigenberg SJ, Ky B, Sun L. Cardiovascular toxicity and risk mitigation with lung cancer treatment. Curr Oncol Rep. (2023) 25:433–444. doi:10.1007/s11912-023-01387-4

168: Hambly R, Kearney N, Hughes R, Fletcher JM, Kirby B. Metformin treatment of hidradenitis suppurativa: effect on metabolic parameters, inflammation, cardiovascular risk biomarkers, and immune mediators. Int J Mol Sci. (2023) 24:6969. doi:10.3390/ijms24086969

169: Koutentakis M, Surma S, Rogula S, Filipiak KJ, Gąsecka A. The effect of a vegan diet on

the cardiovascular system. J Cardiovasc Dev Dis. (2023) 10:94. doi:10.3390/jcdd10030094

170: Crocetto F, Ferro M, Buonerba C, Bardi L, Dolce P, Scafuri L, et al. Comparing cardiovascular adverse events in cancer patients: a meta-analysis of combination therapy with angiogenesis inhibitors and immune checkpoint inhibitors versus angiogenesis inhibitors alone. Crit Rev Oncol Hematol. (2023) 188:104059. doi:10.1016/j.critrevonc.2023.104059

171: Darouiche K, Devin S, Valdebenito S, Ajasin D, Eugenin E. Cocaine-induced cardiovascular and immune dysfunction: emerging mechanisms. Physiology (Bethesda). (2026) 41:0. doi:10.1152/physiol.00004.2025

172: Zanetti HR, Soares VL, Soares WF, Lourenço CL, Gonçalves A, Lopes LT, et al. Physical capacity, body composition and immune-inflammatory markers are associated with risk of cardiovascular disease in sedentary people living with HIV. Minerva Cardiol Angiol. (2023) 71:5–11. doi:10.23736/S2724-5683.21.05578-X

173. Oikawa M, Ishida T, Takeishi Y. Cancer therapeutics-related cardiovascular dysfunction: basic mechanisms and clinical manifestation. J Cardiol. (2023) 81:253–259. doi:10.1016/j.jjcc.2022.04.006

174: Zhang Y, Fu H, Kang X, Qiu Z, Yan H, He Q, et al. Advancements in research on the cardiovascular toxicity caused by TEC family kinases inhibitors. Front Pharmacol. (2026) 16:1726458. doi:10.3389/fphar.2025.1726458

175. Sharma R, Kashyap J, Olanrewaju OA, et al. Cardio-oncology: managing cardiovascular complications of cancer therapies. Cureus. (2023) 15:e51038. doi:10.7759/cureus.51038

176: Sukumaran L, Kunisaki KM, Bakewell N, et al. Association between inflammatory biomarker profiles and cardiovascular risk in individuals with and without HIV. AIDS. (2023) 37:595–603. doi:10.1097/QAD.0000000000003462

177: Rødevand L, Rahman Z, Hindley GFL, et al. Characterizing the shared genetic underpinnings of schizophrenia and cardiovascular disease risk factors. Am J Psychiatry. (2023) 180:815–826. doi:10.1176/appi.ajp.20220660

178: Sciuto M, Catanzaro R. Composition of gut microbiota and its correlations with neurological, intestinal, cardiovascular and metabolic diseases. Acta Microbiol Immunol Hung. (2023) 70:259–271. doi:10.1556/030.2023.02134

179: Cao Q, Du X, Jiang XY, et al. Phenome-wide association study and precision medicine of cardiovascular diseases in the post-COVID-19 era. Acta Pharmacol Sin. (2023) 44:2347–2357. doi:10.1038/s41401-023-01119-1

180: Sree Kumar H, Wisner AS, Refsnider JM, Martyniuk CJ, Zubcevic J. Small fish, big discoveries: zebrafish shed light on microbial biomarkers for neuro-immune-cardiovascular health. Front Physiol. (2023) 14:1186645. doi:10.3389/fphys.2023.1186645

181: Feng Z, Hu Y, Yu S, et al. Exercise in cold: friend rather than foe to cardiovascular health. Life Sci. (2023) 328:121923. doi:10.1016/j.lfs.2023.121923

182: Carullo N, Fabiano G, D’Agostino M, et al. New insights on the role of marinobufagenin from bench to bedside in cardiovascular and kidney diseases. Int J Mol Sci. (2023) 24:11186. doi:10.3390/ijms241311186

183: Liu C, Xu X, He X, et al. Activation of the Nrf-2/HO-1 signalling axis can alleviate metabolic syndrome in cardiovascular disease. Ann Med. (2023) 55:2284890. doi:10.1080/07853890.2023.2284890

184: Bele A, Wagh V, Munjewar PK. A comprehensive review on cardiovascular complications of COVID-19: unraveling the link to bacterial endocarditis. Cureus. (2023) 15:e44019. doi:10.7759/cureus.44019

185: Peluzzo AM, Bkhache M, Do LNH, Autieri MV, Liu X. Differential regulation of lymphatic junctional morphology and the potential effects on cardiovascular diseases. Front Physiol. (2023) 14:1198052. doi:10.3389/fphys.2023.1198052

186: Moulana M. Androgen-induced cardiovascular risk in polycystic ovary syndrome: the role of T lymphocytes. Life (Basel). (2023) 13:1010. doi:10.3390/life13041010

187: Atzeni F, Maiani S, Corda M, Rodríguez-Carrio J. Diagnosis and management of cardiovascular risk in rheumatoid arthritis: main challenges and research agenda. Expert Rev Clin Immunol. (2023) 19:279–292. doi:10.1080/1744666X.2023.2170351

188: Slusher AL, Acevedo EO. Stress-induced proinflammatory adaptations: plausible mechanisms for the link between stress and cardiovascular disease. Front Physiol. (2023) 14:1124121. doi:10.3389/fphys.2023.1124121

189: Quagliariello V, Bisceglia I, Berretta M, et al. PCSK9 inhibitors in cancer patients treated with immune-checkpoint inhibitors to reduce cardiovascular events: new frontiers in cardioncology. Cancers (Basel). (2023) 15:1397. doi:10.3390/cancers15051397

190: Mewborn EK, Wright DB, Wicks MN. Clinical utility of inflammatory and genetic biomarkers for cardiovascular disease prevention, categorization, and treatment. J Am Assoc Nurse Pract. (2023) 35:404–412. doi:10.1097/JXX.0000000000000901

191: Matsumori A. Nuclear factor-κB is a prime candidate for the diagnosis and control of

inflammatory cardiovascular disease. Eur Cardiol. (2023) 18:e40. doi:10.15420/ecr.2023.10

192: Ortiz-Whittingham LR, Baumer Y, Pang APS, et al. Associations between neighborhood socioeconomic deprivation, IFNγ, and HDL particle size. Psychoneuroendocrinology. (2023) 157:106346. doi:10.1016/j.psyneuen.2023.106346

193: Lugonja SI, Pantic IL, Milovanovic TM, et al. Atherosclerotic cardiovascular disease in inflammatory bowel disease: the role of chronic inflammation and platelet aggregation. Medicina (Kaunas). (2023) 59:554. doi:10.3390/medicina59030554

194: Davis KM, Knauft K, Lewis L, et al. The heart of Detroit study: psychosocial determinants of cardiovascular disease risk. BMC Psychiatry. (2023) 23:766. doi:10.1186/s12888-023-05148-2

195: Zheng H, Cao P, Su Z, Xia L. Insights into IL-10-producing regulatory B cells in cardiovascular disorders. J Leukoc Biol. (2023) 114:315–324. doi:10.1093/jleuko/qiad066

196: Felix AS, Nolan TS, Glover LM, et al. The modifying role of resilience on allostatic load and cardiovascular disease risk. J Racial Ethn Health Disparities. (2023) 10:2124–2135. doi:10.1007/s40615-022-01392-6

197: Lee K, Lindenfeld L, Echevarria M, et al. Cardiovascular profile of contemporary treatments of renal cell carcinoma. Int J Cardiol. (2023) 380:40–46. doi:10.1016/j.ijcard.2023.03.040

198: Zapater A, Gracia-Lavedan E, Torres G, et al. Proteomic profiling for prediction of recurrent cardiovascular events in acute coronary syndrome with obstructive sleep apnea. Biomed Pharmacother. (2023) 158:114125. doi:10.1016/j.biopha.2022.114125

199: Vancheri F, Longo G, Vancheri E, Henein MY. Mental stress and cardiovascular health –

Part I. J Clin Med. (2022) 11:3353. doi:10.3390/jcm11123353

200.Bhagat A, Shrestha P, Kleinerman ES. The innate immune system in cardiovascular diseases and its role in doxorubicin-induced cardiotoxicity. Int J Mol Sci. (2022) 23:14649. doi:10.3390/ijms232314649

201: Girard D, Vandiedonck C. How dysregulation of the immune system promotes diabetes mellitus and cardiovascular complications. Front Cardiovasc Med. (2022) 9:991716. doi:10.3389/fcvm.2022.991716

202: Murphy MSQ, Benton SJ, Cox B, et al. Maternal immune cell and cytokine profiles to predict cardiovascular risk after preeclampsia. J Clin Med. (2022) 11:4185. doi:10.3390/jcm11144185

203: Ji RC. The role of lymphangiogenesis in cardiovascular diseases and heart transplantation. Heart Fail Rev. (2022) 27:1837–1856. doi:10.1007/s10741-021-10188-5

204: Wilcox NS, Rotz SJ, Mullen M, et al. Sex-specific cardiovascular risks of cancer and its therapies. Circ Res. (2022) 130:632–651. doi:10.1161/CIRCRESAHA.121.319901

205: Perkins MV, Joseph SB, Dittmer DP, Mackman N. Cardiovascular disease and thrombosis in HIV infection. Arterioscler Thromb Vasc Biol. (2023) 43:175–191. doi:10.1161/ATVBAHA.122.318232

206: Longpré-Poirier C, Dougoud J, Jacmin-Park S, et al. Sex and gender and allostatic mechanisms of cardiovascular risk and disease. Can J Cardiol. (2022) 38:1812–1827. doi:10.1016/j.cjca.2022.09.011

207: Fu Y, Zhang J, Xu L, et al. Developing an immune-related seven-gene signature in COVID-19 and cardiovascular disease. J Cardiovasc Dev Dis. (2022) 9:450. doi:10.3390/jcdd9120450

208: Carnevale D. Neuroimmune axis of cardiovascular control: mechanisms and therapeutic implications. Nat Rev Cardiol. (2022) 19:379–394. doi:10.1038/s41569-022-00678-w

209: van der Heijden CDCC, Bode M, Riksen NP, Wenzel UO. The role of the mineralocorticoid receptor in immune cells in cardiovascular disease. Br J Pharmacol. (2022) 179:3135–3151. doi:10.1111/bph.15782

210: Liu Y, Yu X, Zhang W, et al. Mechanistic insight into premature atherosclerosis in systemic lupus erythematosus. J Autoimmun. (2022) 132:102863. doi:10.1016/j.jaut.2022.102863

211: Henrion D. Modulating the immune response to reduce hypertension-associated cardiovascular damage. J Clin Invest. (2022) 132:e158280. doi:10.1172/JCI158280

212: Turner ME, Bartoli-Leonard F, Aikawa E. Innate immunity in extracellular vesicle-mediated cardiovascular calcification. Immunol Rev. (2022) 312:20–37. doi:10.1111/imr.13134

213: Wang F, Wu X. Cardiovascular toxicities associated with immune checkpoint inhibitors: an updated analysis. J Clin Pharm Ther. (2022) 47:1576–1584. doi:10.1111/jcpt.13707

214: Tumenbayar BI, Pham K, Biber JC, Drewes R, Bae Y. Transcriptomic and multi-scale network analyses reveal key drivers of cardiovascular disease. IEEE Trans Mol Biol Multiscale Commun. (2025) 11:78–90. doi:10.1109/tmbmc.2024.3501576

215: Biondi-Zoccai G, Weber BN, Abbate A, Booz GW. Cardiac immunotherapy, immuno-cardiology, and the future of cardiovascular pharmacology. J Cardiovasc Pharmacol. (2025) 85:308–311. doi:10.1097/FJC.0000000000001687].

It should be [1.Libby P. The changing landscape of atherosclerosis. Nature (2021) 592:524--533. doi: 10.1038/s41586-021-03392-8

2. Jaiswal S, Natarajan P, Silver AJ, Gibson CJ, Bick AG, Shvartz E, et al. Clonal hematopoiesis and risk of atherosclerotic cardiovascular disease. N Engl J Med (2017) 377:111--121. doi: 10.1056/NEJMoa1701719

10. Libby P, Lichtman AH, Hansson GK. Immune effector mechanisms implicated in atherosclerosis: from mice to humans. Immunity (2013) 38:1092--104. doi: 10.1016/j.immuni.2013.06.009

12. Furman D, Campisi J, Verdin E, Carrera-Bastos P, Targ S, Franceschi C, et al. Chronic inflammation in the etiology of disease across the life span. Nat Med (2019) 25:1822--1832. doi: 10.1038/s41591-019-0675-0

15. Miller GE, Passarelli V, Chen E, Kloog I, Wright RJ, Amini H. Ambient PM2.5 and specific sources increase inflammatory cytokine responses to stimulators and reduce sensitivity to inhibitors. Environ Res (2024) 252(Pt 2):118964. doi: 10.1016/j.envres.2024.118964

17. Wolf D, Ley K. Immunity and inflammation in atherosclerosis. Circ Res (2019) 124:315--327. doi: 10.1161/CIRCRESAHA.118.313

18. Oren O, Small AM, Lin AE, Libby P. Managing cardiovascular risk in clonal hematopoiesis of indeterminate potential: JACC: CardioOncology short-form primer. JACC CardioOncol (2025) 7:496--500. doi: 10.1016/j.jaccao.2025.05.013

19. Jaiswal S, Ebert BL. Clonal hematopoiesis in human aging and disease. Science (2019) 366:eaan4673. doi: 10.1126/science.aan4673

20. Swirski FK, Nahrendorf M. Leukocyte behavior in atherosclerosis, myocardial infarction, and heart failure. Science (2013) 339:161--6. doi: 10.1126/science.123071922.

22. Netea MG, Domínguez-Andrés J, Barreiro LB, Chavakis T, Divangahi M, Fuchs E, et al. Defining trained immunity and its role in health and disease. Nat Rev Immunol (2020) 20:375--388. doi: 10.1038/s41577-020-0285-6

23. França CN, Izar MCO, Hortêncio MNS, do Amaral JB, Ferreira CES, Tuleta ID, et al. Monocyte subtypes and the CCR2 chemokine receptor in cardiovascular disease. Clin Sci (Lond) (2017) 131(12):1215-1224. doi: 10.1042/CS20170009

24. Schuermans A, Honigberg MC. Clonal haematopoiesis in cardiovascular disease: prognostic role and novel therapeutic target. Nat Rev Cardiol (2025) 22:845--856. doi: 10.1038/s41569-025-01148-9

25. Colin S, Chinetti-Gbaguidi G, Staels B. Macrophage phenotypes in atherosclerosis. Immunol Rev (2014) 262:153--66. doi: 10.1111/imr.12218

26. Murray PJ, Wynn TA. Protective and pathogenic functions of macrophage subsets. Nat Rev Immunol (2011) 11:723--37. doi: 10.1038/nri3073

27. Tabas I, Bornfeldt KE. Macrophage phenotype and function in different stages of atherosclerosis. Circ Res (2016) 118:653--67. doi: 10.1161/CIRCRESAHA.115.306256

28. Murray PJ. Macrophage polarization. Annu Rev Physiol (2017) 79:541--66. doi: 10.1146/annurev-physiol-022516-034339

29. Stöger JL, Gijbels MJ, van der Velden S, Manca M, van der Loos CM, Biessen EA, et al. Distribution of macrophage polarization markers in human atherosclerosis. Atherosclerosis (2012) 225(2):461-8. doi: 10.1016/j.atherosclerosis.2012.09.013

30. Findeisen HM, Voges VC, Braun LC, Sonnenberg J, Schwarz D, Körner H, et al. LXRα regulates oxLDL-induced trained immunity in macrophages. Int J Mol Sci (2022) 23:6166. doi: 10.3390/ijms23116166

31. Navarro MDC, Hinchado MD, Bote E, Gálvez I, Otero E, Palomino-Segura M, et al. Obesogenic inflammatory memory: a new concept related to the dangerous effects of weight cycling. Biomolecules (2026) 16(2):193. doi: 10.3390/biom16020193

32. Hata M, Andriessen EMMA, Hata M, Diaz-Marin R, Fournier F, Crespo-Garcia S, et al. Past history of obesity triggers persistent epigenetic changes in innate immunity and exacerbates neuroinflammation. Science (2023) 379:45--62. doi: 10.1126/science.abj8894

33. Zhang W, Cai Z, Ma D, Liu M, Wang J, Sun L, et al. Local adaptive immunity in atherosclerosis with T cell activation by aortic dendritic cells accelerates pathogenesis. iScience (2024) 27(11):111144. doi: 10.1016/j.isci.2024.111144

34. Saigusa R, Winkels H, Ley K. T cell subsets and functions in atherosclerosis. Nat Rev Cardiol (2020) 17:387--401. doi: 10.1038/s41569-020-0352-5

35. Ma L, Yang W, Gao W, Liu X, Dong M, An G, et al. IL-17 as a therapeutic target in cardiovascular diseases: mechanistic insights and translational opportunities. Pharmacol Res (2025) 219:107879. doi: 10.1016/j.phrs.2025.107879

36. Taleb S, Tedgui A, Mallat Z. IL-17 and Th17 cells in atherosclerosis: subtle and contextual roles. Arterioscler Thromb Vasc Biol (2015) 35:258--64. doi: 10.1161/ATVBAHA.114.303567

37. Yu J, Xu W, Dong Q, Ji Q, Cheng M, Hu D, et al. Latency-associated peptide (LAP)+CD4+ regulatory T cells prevent atherosclerosis by modulating macrophage polarization. Clin Immunol (2023) 255:109767. doi: 10.1016/j.clim.2023.109767

38. Zhang YS, Chen YQ. Dysfunctional regulatory T cell: may be an obstacle to immunotherapy in cardiovascular diseases. Biomed Pharmacother (2024) 173:116359. doi: 10.1016/j.biopha.2024.116359

39. Mor A, Planer D, Luboshits G, Afek A, Metzger S, Chajek-Shaul T, et al. Role of naturally occurring CD4+ CD25+ regulatory T cells in experimental atherosclerosis. Arterioscler Thromb Vasc Biol (2007) 27:893--900. doi: 10.1161/01.ATV.0000259365.31469.89

40. Bäck M, Yurdagul A Jr, Tabas I, Öörni K, Kovanen PT. Inflammation and its resolution in atherosclerosis: mediators and therapeutic opportunities. Nat Rev Cardiol (2019) 16:389--406. doi: 10.1038/s41569-019-0169-2

41. Levi M, van der Poll T, Büller HR. Bidirectional relation between inflammation and coagulation. Circulation (2004) 109:2698--704. doi: 10.1161/01.CIR.0000131660.51520.9A

42. Jackson SP, Darbousset R, Schoenwaelder SM. Thromboinflammation: challenges of therapeutically targeting coagulation and other host defense mechanisms. Blood (2019) 133:906--918. doi: 10.1182/blood-2018-11-882993

43. Bevilacqua MP, Schleef RR, Gimbrone MA Jr, Loskutoff DJ. Regulation of the fibrinolytic system of cultured human vascular endothelium by interleukin 1. J Clin Invest (1986) 78:587--91. doi: 10.1172/JCI112613

44. Martinez Bravo G, Annarapu G, Carmona E, Nawarskas J, Clark R, Novelli E, et al. Platelets in Thrombosis and Atherosclerosis: A Double-Edged Sword. Am J Pathol (2024) 194(9):1608-1621. doi: 10.1016/j.ajpath.2024.05.010

45. Cerletti C, Tamburrelli C, Izzi B, Gianfagna F, de Gaetano G. Platelet-leukocyte interactions in thrombosis. Thromb Res (2012) 129:263--6. doi: 10.1016/j.thromres.2011.10.010

46. Weber C. Platelets and chemokines in atherosclerosis: partners in crime. Circ Res (2005) 96:612--6. doi: 10.1161/01.RES.0000160077.

47. Heemskerk JW, Bevers EM, Lindhout T. Platelet activation and blood coagulation. Thromb Haemost (2002) 88:186--93

48. Li H, Shan W, Zhao X, Sun W. Neutrophils: linking inflammation to thrombosis and unlocking new treatment horizons. Int J Mol Sci (2025) 26:1965. doi: 10.3390/ijms26051965

49. Castanheira FVS, Kubes P. Neutrophils and NETs in modulating acute and chronic inflammation. Blood (2019) 133(20):2178-2185. doi: 10.1182/blood-2018-11-844530

50. Martinod K, Wagner DD. Thrombosis: tangled up in NETs. Blood (2014) 123:2768--76. doi: 10.1182/blood-2013-10-463646

51. Papayannopoulos V. Neutrophil extracellular traps in immunity and disease. Nat Rev Immunol (2018) 18(2):134-147. doi: 10.1038/nri.2017.105

52. Xu X, Wu Y, Xu S, Yin Y, Ageno W, De Stefano V, et al. Clinical significance of neutrophil extracellular traps biomarkers in thrombosis. Thromb J (2022) 20(1):63. doi: 10.1186/s12959-022-00421-y

53. Steinhoff M, Buddenkotte J, Shpacovitch V, Rattenholl A, Moormann C, Vergnolle N, et al. Proteinase-activated receptors: transducers of proteinase-mediated signaling in inflammation and immune response. Endocr Rev (2005) 26(1):1-43. doi: 10.1210/er.2003-0025

54. Coughlin SR. Thrombin signalling and protease-activated receptors. Nature (2000) 407(6801):258-64. doi: 10.1038/35025229

55. Cimmino G, Di Serafino L, Cirillo P. Pathophysiology and mechanisms of acute coronary syndromes: atherothrombosis, immune-inflammation, and beyond. Expert Rev Cardiovasc Ther (2022) 20:351--62. doi: 10.1080/14779072.2022.2074836

56. Iba T, Levy JH. Inflammation and thrombosis: roles of neutrophils, platelets and endothelial cells and their interactions in thrombus formation during sepsis. J Thromb Haemost (2018) 16:231--241. doi: 10.1111/jth.13911

57. O’Neill LA, Kishton RJ, Rathmell J. A guide to immunometabolism for immunologists. Nat Rev Immunol (2016) 16:553--65. doi: 10.1038/nri.2016.70

58. Buck MD, Sowell RT, Kaech SM, Pearce EL. Metabolic instruction of immunity. Cell (2017) 169(4):570-586. doi: 10.1016/j.cell.2017.04.004

59. Fragasso G, Stolfo D, Anker MS, Bayes-Genis A, Chioncel O, Heymans S, et al. The crosstalk between immune activation and metabolism in heart failure. A scientific statement of the Heart Failure Association of the ESC. Eur J Heart Fail (2025) 27:1700--1719. doi: 10.1002/ejhf.3703

60. Pearce EL, Pearce EJ. Metabolic pathways in immune cell activation and quiescence. Immunity (2013) 38(4):633-43. doi: 10.1016/j.immuni.2013.04.005

61. O’Neill LA, Pearce EJ. Immunometabolism governs dendritic cell and macrophage function. J Exp Med (2016) 213:15--23. doi: 10.1084/jem.20151570

62. Freemerman AJ, Johnson AR, Sacks GN, Milner JJ, Kirk EL, Troester MA, et al. Metabolic reprogramming of macrophages: glucose transporter 1 (GLUT1)-mediated glucose metabolism drives a proinflammatory phenotype. J Biol Chem (2014) 289:7884--96. doi: 10.1074/jbc.M113.522037

63. Huang SC, Everts B, Ivanova Y, O’Sullivan D, Nascimento M, Smith AM, et al. Cell-intrinsic lysosomal lipolysis is essential for alternative activation of macrophages. Nat Immunol (2014) 15(9):846-55. doi: 10.1038/ni.2956

64. Heimlich JB, Bhat P, Parker AC, Jenkins MT, Vlasschaert C, Ulloa J, et al. Multiomic profiling of human clonal hematopoiesis reveals genotype and cell-specific inflammatory pathway activation. Blood Adv (2024) 8(14):3665-3678. doi: 10.1182/bloodadvances.2023011445

65. Getachew F, Hu J, Benamar M. Metabolic regulation of regulatory T cells: mechanisms, heterogeneity, and implications in disease. Front Immunol (2025) 16:1729690. doi: 10.3389/fimmu.2025.1729690

66. Pearce EL, Poffenberger MC, Chang CH, Jones RG. Fueling Immunity: Insights into Metabolism and Lymphocyte Function. Science (2013) 342(6155):1242454. doi: 10.1126/science.1242454

67. Pålsson-McDermott EM, O’Neill LAJ. Gang of 3: how the Krebs cycle-linked metabolites itaconate, succinate, and fumarate regulate macrophages and inflammation. Cell Metab (2025) 37:1049--59. doi: 10.1016/j.cmet.2025.03.004

68. Tannahill GM, Curtis AM, Adamik J, Palsson-McDermott EM, McGettrick AF, Goel G, et al. Succinate is an inflammatory signal that induces IL-1β through HIF-1α. Nature (2013) 496:238--42. doi: 10.1038/nature11986

69. Bourner LA, Chung LA, Long H, McGettrick AF, Xiao J, Roth K, et al. Endogenously produced itaconate negatively regulates innate-driven cytokine production and drives global ubiquitination in human macrophages. Cell Rep (2024) 43(8):114570. doi: 10.1016/j.celrep.2024.114570

70. Ziogas A, Novakovic B, Ventriglia L, Galang N, Tran KA, Li W, et al. Long-term histone lactylation connects metabolic and epigenetic rewiring in innate immune memory. Cell (2025) 188(11):2992-3012.e16. doi: 10.1016/j.cell.2025.03.048

71. Saitoh S, Van Wijk K, Nakajima O. Crosstalk between metabolic disorders and immune cells. Int J Mol Sci (2021) 22:10017. doi: 10.3390/ijms22181001773: Maguire EM, Pearce SWA, Xiao Q. Foam cell formation: a new target for fighting atherosclerosis and cardiovascular disease. Vascul Pharmacol (2019) 112:54--71. doi: 10.1016/j.vph.2018.08.002

73. Korbecki J, Bajdak-Rusinek K. The effect of palmitic acid on inflammatory response in macrophages: an overview of molecular mechanisms. Inflamm Res (2019) 68:915--932. doi: 10.1007/s00011-019-01273-5

74. Weinberg SE, Chandel NS. Mitochondria reactive oxygen species signaling-dependent immune responses in macrophages and T cells. Immunity (2025) 58:1904--21. doi: 10.1016/j.immuni.2025.07.012

75. Zhang R, Jiang Y, Zhang G, Zeng W, Suo Y, Zhang F, et al. Mitochondrial DNA in atherosclerosis: Mechanisms, biomarker potential, and therapeutic perspectives. Int Immunopharmacol (2025) 152:114449. doi: 10.1016/j.intimp.2025.114449

76. Eelen G, de Zeeuw P, Treps L, Harjes U, Wong BW, Carmeliet P. Endothelial cell metabolism. Physiol Rev (2018) 98:3--58. doi: 10.1152/physrev.00001.2017

77. Chee YJ, Dalan R, Cheung C. The interplay between immunity, inflammation and endothelial dysfunction. Int J Mol Sci (2025) 26:1708. doi: 10.3390/ijms26041708

78. Certo M, Rahimzadeh M, Mauro C. Immunometabolism in atherosclerosis: a new understanding of an old disease. Trends Biochem Sci (2024) 49:791--803. doi: 10.1016/j.tibs.2024.06.003

79. Lin R, Yi Z, Wang J, Geng S, Li L. Generation of resolving memory neutrophils through pharmacological training with 4-PBA or genetic deletion of TRAM. Cell Death Dis (2022) 13(4):345. doi: 10.1038/s41419-022-04809-6

80. Geng S, Lu R, Zhang Y, Wu Y, Xie L, Caldwell BA, et al. Monocytes reprogrammed by 4-PBA potently contribute to the resolution of inflammation and atherosclerosis. Circ Res. (2024) 135(8):856--872. doi:10.1161/CIRCRESAHA.124.325023

81. Dai J, Zhou X, Yuan K, Huang K, Zhang Y. Trained immunity-like memory in vascular structural cells: metabolic-epigenetic reprogramming as a driving mechanism of atherosclerosis and residual cardiovascular risk. J Inflamm Res (2026) 19:599156. doi: 10.2147/JIR.S599156

82. Liang S, Tao W, Qu D, Ji P, Hao Z, Wei M, et al. Treatment of clonal hematopoiesis associated atherosclerosis via vaccine-based clearance of the mutant cells. J Adv Res (2026) S2090-1232(26)00065-2. doi: 10.1016/j.jare.2026.01.040

84. Comşa AD, Comşa H, Cismaru G, Roşu R, Pop D. Anti-inflammatory Pathways of Novel Anti-diabetic Therapies. A Literature Review. *In Vivo* (2026) 40(1):600-627. doi: 10.21873/invivo.14224

85. Miteva K, Anker MS, Fechner H, Lehmann L, Van Linthout S. Cancer-Related Immune Therapies: Bidirectional Implications From Cardiotoxicity to Emerging Cardiovascular Therapeutics: JACC CardioOncology State-of-the-Art Review. JACC CardioOncol (2025) 7(5):523-537. doi: 10.1016/j.jaccao.2025.06.008

87. Du H, Wang J, Wang Z. Cardiovascular adverse effects of immunotherapy in cancer: insights and implications. Front Oncol (2025) 15:1601808. doi: 10.3389/fonc.2025.1601808

88. Ridker PM, Everett BM, Thuren T, MacFadyen JG, Chang WH, Ballantyne C, et al. Antiinflammatory therapy with canakinumab for atherosclerotic disease. N Engl J Med (2017) 377:1119--1131. doi: 10.1056/NEJMoa1707914

89. Tardif JC, Kouz S, Waters DD, Bertrand OF, Diaz R, Maggioni AP, et al. Efficacy and safety of low-dose colchicine after myocardial infarction. N Engl J Med (2019) 381:2497--2505. doi: 10.1056/NEJMoa1912388

90. Netea MG, Joosten LA, Latz E, Mills KH, Natoli G, Stunnenberg HG, et al. Trained immunity: A program of innate immune memory in health and disease. Science (2016) 352(6284):aaf1098. doi: 10.1126/science.aaf1098

91. De Marco Castro E, Calder PC, Roche HM. β-1,3/1,6-glucans and immunity: state of the art and future directions. Mol Nutr Food Res (2021) 65:e1901071. doi: 10.1002/mnfr.201901071

92. Schlüter T, van Elsas Y, Priem B, Ziogas A, Netea MG. Trained immunity: induction of an inflammatory memory in disease. Cell Res (2025) 35(11):792-802. doi: 10.1038/s41422-025-01171-y

93. Ochando J, Mulder WJM, Madsen JC, Netea MG, Duivenvoorden R. Trained immunity - basic concepts and contributions to immunopathology. Nat Rev Nephrol (2023) 19:23--37. doi: 10.1038/s41581-022-00633-5

94. Chen M, Kang X, Zhang Y, Liu Y. Trained immunity: a link between risk factors and cardiovascular disease. Br J Pharmacol (2025) 182:4782--4800. doi: 10.1111/bph.16472

95. Li J, Lee DH, Hu J, Tabung FK, Li Y, Bhupathiraju SN, et al. Dietary Inflammatory Potential and Risk of Cardiovascular Disease Among Men and Women in the U.S. J Am Coll Cardiol (2020) 76(19):2181-2193. doi: 10.1016/j.jacc.2020.09.535

96. Zhao B, Wan J, Zhou H, Yang J, Wan H. Trained immunity in atherosclerosis: plasticity, metabolic-vascular axis, and AI-driven precision remodeling. Front Immunol (2025) 16:1669796. doi: 10.3389/fimmu.2025.1669796

97. Christ A, Günther P, Lauterbach MAR, Duewell P, Biswas D, Pelka K, et al. Western Diet Triggers NLRP3-Dependent Innate Immune Reprogramming. Cell (2018) 172(1-2):162-175.e14. doi: 10.1016/j.cell.2017.12.013

98. Miao F, Chen Z, Zhang L, Wang J, Gao H, Wu X, et al. RNA-sequencing analysis of high glucose-treated monocytes reveals novel transcriptome signatures and associated epigenetic profiles. Physiol Genomics (2013) 45:287--99. doi: 10.1152/physiolgenomics.00001.2013

99. Ramos-Lopez O, Milagro FI, Riezu-Boj JI, Martinez JA. Epigenetic signatures underlying inflammation: an interplay of nutrition, physical activity, metabolic diseases, and environmental factors for personalized nutrition. Inflamm Res (2021) 70(1):29-49. doi: 10.1007/s00011-020-01425-y

100. Merbecks AJ, Mennicken C, de Graaf DM, Shkarina K, Wagner T, Latz E. Western lifestyle linked to maladaptive trained immunity. Elife (2026) 15:e105835. doi: 10.7554/eLife.105835

101. Cai H, Chen X, Liu Y, Chen Y, Zhong G, Chen X, et al. Lactate activates trained immunity by fueling the tricarboxylic acid cycle and regulating histone lactylation. Nat Commun (2025) 16(1):3230. doi: 10.1038/s41467-025-58563-2

102. Yukino-Iwashita M, Nagatomo Y, Kawai A, Taruoka A, Yumita Y, Kagami K, et al. Short-Chain Fatty Acids in Gut-Heart Axis: Their Role in the Pathology of Heart Failure. J Pers Med (2022) 12(11):1805. doi: 10.3390/jpm12111805

103. Ma H, Yang L, Liu Y, Yan R, Wang R, Zhang P, et al. Butyrate suppresses atherosclerotic inflammation by regulating macrophages and polarization via GPR43/HDAC-miRNAs axis in ApoE-/- mice. PLoS One (2023) 18(3):e0282685. doi: 10.1371/journal.pone.0282685

104. Lopez-Garcia E, Schulze MB, Meigs JB, Manson JE, Rifai N, Stampfer MJ, et al. Consumption of trans fatty acids is related to plasma biomarkers of inflammation and endothelial dysfunction. J Nutr (2005) 135:562--6. doi: 10.1093/jn/135.3.562

105. Johnson CSC, Shively CA, Michalson KT, Lea AJ, DeBo RJ, Howard TD, et al. Contrasting effects of Western vs Mediterranean diets on monocyte inflammatory gene expression and social behavior in a primate model. eLife (2021) 10:e68293. doi: 10.7554/eLife.68293

106. Grigorescu A, Riza AL, Streata I, Netea MG. Metabolic dysregulation of trained immunity in immune aging and the impact of dietary patterns. Am J Physiol Cell Physiol (2025) 329:C456--C470. doi: 10.1152/ajpcell.00153.2025

107. Moro T, Tinsley G, Pacelli FQ, Marcolin G, Bianco A, Paoli A. Twelve Months of Time-restricted Eating and Resistance Training Improves Inflammatory Markers and Cardiometabolic Risk Factors. Med Sci Sports Exerc (2021) 53(12):2577-2585. doi: 10.1249/MSS.0000000000002738

108. Chaix A, Manoogian ENC, Melkani GC, Panda S. Time-restricted eating to prevent and manage chronic metabolic diseases. Annu Rev Nutr (2019) 39:291--315. doi: 10.1146/annurev-nutr-082018-124320

109. Rahimi N, Gupta S. Biochemistry, ketogenesis. In: StatPearls. Treasure Island (FL): StatPearls Publishing; 2025.

110. Youm YH, Nguyen KY, Grant RW, Goldberg EL, Bodogai M, Kim D, et al. The ketone metabolite β-hydroxybutyrate blocks NLRP3 inflammasome-mediated inflammatory disease. Nat Med (2015) 21:263--9. doi: 10.1038/nm.3804

111. Huang C, Wang J, Liu H, Huang R, Yan X, Song M, et al. Ketone body β-hydroxybutyrate ameliorates colitis by promoting M2 macrophage polarization through the STAT6-dependent signaling pathway. BMC Med (2022) 20(1):148. doi: 10.1186/s12916-022-02352-x

112. Qian J, Fang Y, Yuan N, Gao X, Lv Y, Zhao C, et al. Innate immune remodeling by short-term intensive fasting. Aging Cell (2021) 20:e13507. doi: 10.1111/acel.

113. Marko DM, Conn MO, Schertzer JD. Intermittent fasting influences immunity and metabolism. Trends Endocrinol Metab (2024) 35:821--833. doi: 10.1016/j.tem.2024.04.014

114. Man K, Loudon A, Chawla A. Immunity around the clock. Science (2016) 354(6315):999-1003. doi: 10.1126/science.aah4966

115. Scheiermann C, Kunisaki Y, Frenette PS. Circadian control of the immune system. Nat Rev Immunol (2013) 13(3):190-8. doi: 10.1038/nri3386

116. Chaix A, Lin T, Ramms B, Cutler RG, Le T, Lopez C, et al. Time-restricted feeding reduces atherosclerosis in LDLR KO mice but not in ApoE knockout mice. Arterioscler Thromb Vasc Biol (2024) 44:2069--2087. doi: 10.1161/ATVBAHA.124.320998

117. Wilkinson MJ, Manoogian ENC, Zadourian A, Lo H, Fakhouri S, Shoghi A, et al. Ten-Hour Time-Restricted Eating Reduces Weight, Blood Pressure, and Atherogenic Lipids in Patients with Metabolic Syndrome. Cell Metab (2020) 31(1):92-104.e5. doi: 10.1016/j.cmet.2019.11.004

118. Horne BD, Muhlestein JB, Anderson JL. Health effects of intermittent fasting: hormesis or harm? A systematic review. Am J Clin Nutr (2015) 102(2):464-70. doi: 10.3945/ajcn.115.109553

119. Kibret KT, Peeters A, Tegegne TK, Mesfin YM, Nichols M. Intermittent fasting for the prevention of cardiovascular disease risks: systematic review and network meta-analysis. Curr Nutr Rep (2025) 14:93. doi: 10.1007/s13668-025-00684-7

120. Zaid NSN, Muhamad AS, Jawis MN, Ooi FK, Mohamed M, Mohamud R, et al. The Effect of Exercise on Immune Response in Population with Increased Risk Factors for Cardiovascular Disease: A Systematic Review. Malays J Med Sci (2024) 31(5):83-108. doi: 10.21315/mjms2024.31.5.6

121. Gustafson B, Hammarstedt A, Andersson CX, Smith U. Inflamed adipose tissue: a culprit underlying the metabolic syndrome and atherosclerosis. Arterioscler Thromb Vasc Biol. 2007 Nov;27(11):2276-83. doi: 10.1161/ATVBAHA.107.147835. Epub 2007 Sep 6. PMID: 17823366.

122. Ross R, Dagnone D, Jones PJ, Smith H, Paddags A, Hudson R, et al. Reduction in obesity and related comorbid conditions after diet-induced weight loss or exercise-induced weight loss in men. A randomized, controlled trial. Ann Intern Med (2000) 133(2):92-103. doi: 10.7326/0003-4819-133-2-200007180-00008

123. Green DJ, Maiorana A, O’Driscoll G, Taylor R. Effect of exercise training on endothelium-derived nitric oxide function in humans. J Physiol (2004) 561:1--25. doi: 10.1113/jphysiol.2004.068197

124. Robinson AT, Banks NF, Jenkins NDM. Exercise as vascular medicine: can exercise combat negative health behaviors from causing endothelial dysfunction? Exerc Sport Mov (2025) 3:e00054. doi: 10.1249/esm.0000000000000054

125. Gleeson M, Bishop NC, Stensel DJ, Lindley MR, Mastana SS, Nimmo MA. The anti-inflammatory effects of exercise: mechanisms and implications for the prevention and treatment of disease. Nat Rev Immunol (2011) 11:607--15. doi: 10.1038/nri3041

126. Dorneles GP, Dos Passos AAZ, Romão PRT, Peres A. New insights about regulatory T cells distribution and function with exercise: the role of immunometabolism. Curr Pharm Des (2020) 26:979--990. doi: 10.2174/1381612826666200305125210

127. Aslam MA, Lee J, Scott Bowen T, Huh JY. Exercise-induced myokines in metabolic regulation: mechanisms, mimetics, and translational potential. Arch Pharm Res (2026) 49:781--819. doi: 10.1007/s12272-026-01624-x

128. Severinsen MCK, Pedersen BK. Muscle-organ crosstalk: the emerging roles of myokines. Endocr Rev (2020) 41:594--609. doi: 10.1210/endrev/bnaa016

129. Chow LS, Gerszten RE, Taylor JM, Pedersen BK, van Praag H, Trappe S, et al. Exerkines in health, resilience and disease. Nat Rev Endocrinol (2022) 18(5):273-289. doi: 10.1038/s41574-022-00641-2

130. Nieman DC, Wentz LM. The compelling link between physical activity and the body’s defense system. J Sport Health Sci (2019) 8:201--217. doi: 10.1016/j.jshs.2018.09.009132: Pedersen BK, Saltin B. Exercise as medicine - evidence for prescribing exercise as therapy in 26 different chronic diseases. Scand J Med Sci Sports (2015) 25(Suppl 3):1-72. doi: 10.1111/sms.12581

132. Pedersen BK, Saltin B. Exercise as medicine - evidence for prescribing exercise as therapy in 26 different chronic diseases. Scand J Med Sci Sports (2015) 25(Suppl 3):1-72. doi: 10.1111/sms.12581

133. Steptoe A, Kivimäki M. Stress and cardiovascular disease: an update on current knowledge. Annu Rev Public Health (2013) 34:337--54. doi: 10.1146/annurev-publhealth-031912-114452

134. Eroume A Egom E. Mind-heart links in ASCVD: evidence for chronic risk, acute triggers, and clinical prevention. Trends Cardiovasc Med (2026) 36:169--77. doi: 10.1016/j.tcm.2025.10.004

135. Feng LS, Wang YM, Liu H, Ning B, Yu HB, Li SL, et al. Hyperactivity in the Hypothalamic-Pituitary-Adrenal Axis: An Invisible Killer for Anxiety and/or Depression in Coronary Artherosclerotic Heart Disease. J Integr Neurosci (2024) 23(12):222. doi: 10.31083/j.jin2312222

136. Chrousos GP. Stress and disorders of the stress system. Nat Rev Endocrinol (2009) 5:374--81. doi: 10.1038/nrendo.2009.106

137. Fioranelli M, Bottaccioli AG, Bottaccioli F, Bianchi M, Rovesti M, Roccia MG. Stress and Inflammation in Coronary Artery Disease: A Review Psychoneuroendocrineimmunology-Based. Front Immunol (2018) 9:2031. doi: 10.3389/fimmu.2018.02031

138. Silverman MN, Sternberg EM. Glucocorticoid regulation of inflammation and its functional correlates: from HPA axis to glucocorticoid receptor dysfunction. Ann N Y Acad Sci (2012) 1261:55--63. doi: 10.1111/j.1749-6632.2012.06633.x

139. Cohen S, Janicki-Deverts D, Doyle WJ, Miller GE, Frank E, Rabin BS, et al. Chronic stress, glucocorticoid receptor resistance, inflammation, and disease risk. Proc Natl Acad Sci U S A (2012) 109:5995--9. doi: 10.1073/pnas.1118355109

140. Nance DM, Sanders VM. Autonomic innervation and regulation of the immune system (1987-2007). Brain Behav Immun (2007) 21(6):736-45. doi: 10.1016/j.bbi.2007.03.008

141. Heidt T, Sager HB, Courties G, Dutta P, Iwamoto Y, Zaltsman A, et al. Chronic variable stress activates hematopoietic stem cells. Nat Med (2014) 20(7):754-758. doi: 10.1038/nm.3589

142. Marwaha K, Cain R, Asmis K, Czaplinski K, Holland N, Mayer DCG, et al. Exploring the complex relationship between psychosocial stress and the gut microbiome: implications for inflammation and immune modulation. J Appl Physiol (1985) (2025) 138(2):518-535. doi: 10.1152/japplphysiol.00652.2024

143. Bischoff SC, Barbara G, Buurman W, Ockhuizen T, Schulzke JD, Serino M, et al. Intestinal permeability--a new target for disease prevention and therapy. BMC Gastroenterol (2014) 14:189. doi: 10.1186/s12876-014-0189-7

144. McEwen BS, Stellar E. Stress and the individual: mechanisms leading to disease. Arch Intern Med (1993) 153:2093--101. doi: 10.1001/archinte.153.18.2093

145. McCrory C, McLoughlin S, Layte R, NiCheallaigh C, O’Halloran AM, Barros H, et al. Towards a consensus definition of allostatic load: a multi-cohort, multi-system, multi-biomarker individual participant data (IPD) meta-analysis. Psychoneuroendocrinology (2023) 153:106117. doi: 10.1016/j.psyneuen.2023.106117

146. McEwen BS. Stress, adaptation, and disease. Allostasis and allostatic load. Ann N Y Acad Sci (1998) 840:33-44. doi: 10.1111/j.1749-6632.1998.tb09546.x

147. Guidi J, Lucente M, Sonino N, Fava GA. Allostatic Load and Its Impact on Health: A Systematic Review. Psychother Psychosom (2021) 90(1):11-27. doi: 10.1159/000510696

148. Polick CS, Harris-Gersten ML, Dennis PA, Noonan D, Hastings SN, Calhoun PS, et al. Allostatic Load, Morbidity, and Mortality Among Older Adults: A Multi-Wave Analysis From the National Health and Aging Trends Study. J Appl Gerontol (2024) 43(8):1052-1059. doi: 10.1177/07334648241230010

149. Slusher AL, Acevedo EO. Stress-induced proinflammatory adaptations: plausible mechanisms for the link between stress and cardiovascular disease. Front Physiol (2023) 14:1124121. doi: 10.3389/fphys.2023.1124121

150. Xu S, Zhang G, Xu Y, Yu C, Wu R, Li Z, et al. Allostatic load-cardiovascular disease associations and the mediating effect of inflammatory factors: a prospective cohort study. Front Cardiovasc Med (2026) 12:1724572. doi: 10.3389/fcvm.2025.1724572

151. Emery O, Carmeli C, Gonseth-Nusslé S, Juster R, Kinnaer C, Nanchen D, et al. Integrative DNA methylation and transcriptome analysis reveal cell-type specific patterns in response to elevated allostatic load. Epigenetics (2025) 20(1):2578552. doi: 10.1080/15592294.2025.2578552

152. Juster RP, McEwen BS, Lupien SJ. Allostatic load biomarkers of chronic stress and impact on health and cognition. Neurosci Biobehav Rev (2010) 35:2--16. doi: 10.1016/j.neubiorev.2009.10.002

153. Lee SC, Tsai PH, Yu KH, Chan TM. Effects of Mind-Body Interventions on Immune and Neuroendocrine Functions: A Systematic Review and Meta-Analysis of Randomized Controlled Trials. Healthcare (Basel) (2025) 13(8):952. doi: 10.3390/healthcare13080952

154. Levine GN, Cohen BE, Commodore-Mensah Y, Fleury J, Huffman JC, Khalid U, et al. Psychological Health, Well-Being, and the Mind-Heart-Body Connection: A Scientific Statement From the American Heart Association. Circulation (2021) 143(10):e763-e783. doi: 10.1161/CIR.0000000000000947

155. Richards SH, Anderson L, Jenkinson CE, Whalley B, Rees K, Davies P, et al. Psychological interventions for coronary heart disease. Cochrane Database Syst Rev (2017) 4:CD002902. doi: 10.1002/14651858.CD002902.pub4

156. Pascoe MC, Thompson DR, Jenkins ZM, Ski CF. Mindfulness mediates the physiological markers of stress: Systematic review and meta-analysis. J Psychiatr Res (2017) 95:156-178. doi: 10.1016/j.jpsychires.2017.08.004

157. Anderson E, Shivakumar G. Effects of exercise and physical activity on anxiety. Front Psychiatry (2013) 4:27. doi: 10.3389/fpsyt.2013.00027

158. Liu Z, Li Y, Mei Z, Li J, Yan X, Cao C. Social relationships and risk of cardio-cerebrovascular diseases: a meta-analysis of longitudinal cohort studies. Soc Psychiatry Psychiatr Epidemiol (2026) 61(5):867-883. doi: 10.1007/s00127-025-03001-3

159. Bueno H, Deaton C, Farrero M, Forsyth F, Braunschweig F, Buccheri S, et al., 2025 ESC Clinical Consensus Statement on mental health and cardiovascular disease: developed under the auspices of the ESC Clinical Practice Guidelines Committee. Eur Heart J (2025) 46(41):4156-4225. doi: 10.1093/eurheartj/ehaf191

160. Su JJ, Lin R, Batalik L, Wong AKC, Grace SL. Psychological eHealth interventions for patients with cardiovascular diseases: systematic review and meta-analysis. J Med Internet Res (2025) 27:e57368. doi: 10.2196

161. Ndejjo R, Hassen HY, Wanyenze RK, Musoke D, Nuwaha F, Abrams S, et al. Community-based interventions for cardiovascular disease prevention in low-and middle-income countries: a systematic review. Public Health Rev (2021) 42:1604018. doi: 10.3389/phrs.2021.1604018

162. Katon WJ, Lin EH, Von Korff M, Ciechanowski P, Ludman EJ, Young B, et al. Collaborative care for patients with depression and chronic illnesses. N Engl J Med (2010) 363:2611--20. doi: 10.1056/NEJMoa1003955

163. Al-Kindi SG, Brook RD, Biswal S, Rajagopalan S. Environmental determinants of cardiovascular disease: lessons learned from air pollution. Nat Rev Cardiol (2020) 17:656--672. doi: 10.1038/s41569-020-0371-2

164. Kounis NG, Hung MY, de Gregorio C, Mplani V, Gogos C, Assimakopoulos SF, et al. Cardio-oncoimmunology: cardiac toxicity, cardiovascular hypersensitivity, and Kounis syndrome. Life (Basel) (2024) 14:400. doi: 10.3390/life14030400

165. Marchini T, Zirlik A, Wolf D. Pathogenic role of air pollution particulate matter in cardiometabolic disease: evidence from mice and humans. Antioxid Redox Signal (2020) 33:263--79. doi: 10.1089/ars.2020.8096

166. Chaulin AM, Sergeev AK. Modern concepts of the role of fine particles (PM2.5) in the genesis of atherosclerosis and myocardial damage: clinical and epidemiological data, the main pathophysiological mechanisms. Curr Cardiol Rev (2023) 19:e170822207573. doi: 10.2174/1573403X18666220817103105

167. Miller MR, Newby DE. Air pollution and cardiovascular disease: car sick. Cardiovasc Res (2020) 116:279--294. doi: 10.1093/cvr/cvz228

168. Pan Z, Gong T, Liang P. Heavy metal exposure and cardiovascular disease. Circ Res (2024) 134:1160--1178. doi: 10.1161/CIRCRESAHA.123.323617

169. Houston MC. The role of mercury and cadmium heavy metals in vascular disease, hypertension, coronary heart disease, and myocardial infarction. Altern Ther Health Med (2007) 13:S128--33. doi: 10.1016/j.atm.2007.03.002

170. Vogel CFA, Van Winkle LS, Esser C, Haarmann-Stemmann T. The aryl hydrocarbon receptor as a target of environmental stressors - Implications for pollution mediated stress and inflammatory responses. Redox Biol (2020) 34:101530. doi: 10.1016/j.redox.2020.101530

171. Wang Z, Zhou Y, Xiao X, Liu A, Wang S, Preston RJS, et al. Inflammation and cardiometabolic diseases induced by persistent organic pollutants and nutritional interventions: Effects of multi-organ interactions. Environ Pollut (2023) 339:122756. doi: 10.1016/j.envpol.2023.122756

172. Shil S, Kumar P, Mumbrekar KD. Cancer therapy-induced cardiotoxicity: mechanisms and mitigations. Heart Fail Rev (2025) 30:1075--92. doi: 10.1007/s10741-025-10531-0

173. Ribas A, Wolchok JD. Cancer immunotherapy using checkpoint blockade. Science (2018) 359:1350--1355. doi: 10.1126/science.aar4060

174. Malode A, Makwana B, Patel V, Khadke S, Parikh A, Bagga A, et al. Cardiotoxicity and peri-operative considerations in immune checkpoint inhibitor and chimeric antigen receptor T-cell therapy: a narrative review. Anaesthesia (2025) 80:25--37. doi: 10.1111/anae.16493

175. Xu L, Wu H, Wu C, Xu Y. Chimeric antigen receptor-T cell therapy-induced cardiotoxicity: pathophysiological mechanisms and pharmacological intervention strategies. Pharmacol Res (2026) 230:108316. doi: 10.1016/j.phrs.2026.108316

176. Liang S, Wang X, Zhu X. Insights from pharmacovigilance and pharmacodynamics on cardiovascular safety signals of NSAIDs. Front Pharmacol (2024) 15:1455212. doi: 10.3389/fphar.2024.1455212

177. Odigwe C, Mulyala R, Malik H, Ruiz B, Riad M, Sayiadeh MA, et al. Emerging role of GLP-1 agonists in cardio-metabolic therapy - Focus on Semaglutide. Am Heart J Plus (2025) 52:100518. doi: 10.1016/j.ahjo.2025.100518

178. Yang HM. GLP-1 agonists in cardiovascular diseases: mechanisms, clinical evidence, and emerging therapies. J Clin Med (2025) 14:6758. doi: 10.3390/jcm14196758

179. Shu B, Chen X, Liu Z, Tang H, Yang B, Fu C. Pleiotropic effects of SGLT2 inhibitors: A focus on macrophage-mediated action. Pharmacol Res (2025) 222:108046. doi: 10.1016/j.phrs.2025.108046

180. Buttice L, Ghani M, Suthakar J, Gnanalingham S, Carande E, Kennedy BWC, et al. The effect of sodium-glucose cotransporter-2 inhibitors on inflammatory biomarkers: A meta-analysis of randomized controlled trials. Diabetes Obes Metab (2024) 26(7):2706-2721. doi: 10.1111/dom.15586

181. Barac A, Murtagh G, Carver JR, Chen MH, Freeman AM, Herrmann J, et al. Cardiovascular health of patients with cancer and cancer survivors: a roadmap to the next level. J Am Coll Cardiol (2015) 65:2739--46. doi: 10.1016/j.jacc.2015.04.059

182. Giannakopoulou SP, Chrysohoou C, Antonopoulou S, Barkas F, Vlachogiannis N, Kravvariti E, et al. Immune-related biochemical markers and 20-year incidence of atherosclerotic cardiovascular disease: the ATTICA study (2002-2022). Atherosclerosis (2025) 405:119212. doi: 10.1016/j.atherosclerosis.2025.119212

183. Liu H, Wang P, Wu LH, Wu F, Zhou X, Li Y, et al. Evaluating immune-inflammatory indices for risk stratification in cardiovascular disease: an umbrella review of systematic reviews and meta-analyses. Diagnostics (Basel) (2025) 15:2862. doi: 10.3390/diagnostics15222862

184. Jiang P, Chen J, Li J. Association of the systemic immune-inflammatory index and systemic inflammatory response index with all-cause and cardiovascular mortality in individuals with metabolic inflammatory syndrome. Eur J Med Res (2025) 30:444. doi: 10.1186/s40001-025-02611-6

185. Lei Y, Chen Y, Hua C, Wang W. Prognostic value of systemic inflammation response index in patients with myocardial infarction: a systematic review and meta-analysis. BMC Cardiovasc Disord (2026) 26(1):280. doi: 10.1186/s12872-026-05633-y

186. Bhat T, Teli S, Rijal J, Bhat H, Raza M, Khoueiry G, et al. Neutrophil to lymphocyte ratio and cardiovascular diseases: a review. Expert Rev Cardiovasc Ther (2013) 11(1):55-9. doi: 10.1586/erc.12.159

187. Oliva A, Vogel B, Sartori S, Cao D, Smith KF, Bay B, et al. Association of neutrophil-to-lymphocyte ratio with clinical outcomes after percutaneous coronary intervention. Eur J Prev Cardiol (2026) 33(5):669-678. doi: 10.1093/eurjpc/zwaf597

188. Lin M, Guo J, Gu Z, Tang W, Tao H, You S, et al. Machine learning and multi-omics integration: advancing cardiovascular translational research and clinical practice. J Transl Med (2025) 23:388. doi: 10.1186/s12967-025-06425-2

189. Khera AV, Kathiresan S. Genetics of coronary artery disease: discovery, biology and clinical translation. Nat Rev Genet (2017) 18:331--344. doi: 10.1038/nrg.2016.160

190. Sarwar N, Butterworth AS, Freitag DF, Gregson J, Willeit P, Gorman DN, et al. Interleukin-6 receptor pathways in coronary heart disease: a collaborative meta-analysis of 82 studies. Lancet (2012) 379(9822):1205-13. doi: 10.1016/S0140-6736(11)61931-4

191. Mokry M, Boltjes A, Slenders L, Bel-Bordes G, Cui K, Brouwer E, et al. Transcriptomic-based clustering of human atherosclerotic plaques identifies subgroups with different underlying biology and clinical presentation. Nat Cardiovasc Res (2022) 1(12):1140-1155. doi: 10.1038/s44161-022-00171-0

192. Fernandez DM, Rahman AH, Fernandez NF, Chudnovskiy A, Amir ED, Amadori L, et al. Single-cell immune landscape of human atherosclerotic plaques. Nat Med (2019) 25(10):1576-1588. doi: 10.1038/s41591-019-0590-4

193. Chhay P, Tang O, Ai L, Cordwell SJ, Gray MP, Yang JYH, et al. Digging deeper into cardiovascular plasma proteomics: opportunities and limitations of current platforms. Circ Genom Precis Med (2025) 18:e005198. doi: 10.1161/CIRCGEN.125.005198

194. Stastna M. Post-translational modifications of proteins in cardiovascular diseases examined by proteomic approaches. FEBS J (2025) 292:28--46. doi: 10.1111/febs.17108

195. Shah SH, Kraus WE, Newgard CB. Metabolomic profiling for the identification of novel biomarkers and mechanisms related to common cardiovascular diseases: form and function. Circulation (2012) 126:1110--20. doi: 10.1161/CIRCULATIONAHA.111.060

196. Zarzour A, Kim HW, Weintraub NL. Epigenetic regulation of vascular diseases. Arterioscler Thromb Vasc Biol (2019) 39:984--90. doi: 10.1161/ATVBAHA.119.312193

197. Lee HT, Oh S, Ro DH, Yoo H, Kwon YW. The Key Role of DNA Methylation and Histone Acetylation in Epigenetics of Atherosclerosis. J Lipid Atheroscler (2020) 9(3):419-434. doi: 10.12997/jla.2020.9.3.419

198. Ya X, Li H, Ge P, Xu Y, Liu Z, Zheng Z, et al. Single-Cell Atlas of Atherosclerosis Patients by Cytof: Circulatory and Local Immune Disorders. Aging Dis (2024) 15(1):245-258. doi: 10.14336/AD.2023.0426-1

199. Liu C, Wu R, Yang H, Yao Y. Immune cell dynamics and their role in cardiac injury: Mechanisms and therapeutic implications. Biomed Pharmacother (2025) 192:118608. doi: 10.1016/j.biopha.2025.118608

200. Toldo S, Abbate A. The NLRP3 inflammasome in acute myocardial infarction. Nat Rev Cardiol (2018) 15:203--214. doi: 10.1038/nrcardio.2017.161

201. Nordestgaard LT, Magni P, Sopić M, Chemaly M, Matic L, Dalila N, et al. Multiomics for Risk Stratification in Atherosclerotic Cardiovascular Disease. Circ Genom Precis Med (2026) 19(2):e005451. doi: 10.1161/CIRCGEN.125.005451

202. Sharma S, Artner T, Preissner KT, Lang IM. Nucleic acid liquid biopsies in cardiovascular disease: cell-free RNA liquid biopsies in cardiovascular disease. Atherosclerosis (2024) 398:118584. doi: 10.1016/j.atherosclerosis.2024.118584

203. Di Febo R, Saeed Z, Serafini F, Brocco D, D’Ascanio F, Pizzi AD, et al. Diagnostic and prognostic roles of endothelial- and platelet-derived extracellular vesicles in cardiovascular diseases. J Transl Med (2025) 23:553. doi: 10.1186/s12967-025-06522-2

204. Cappucci IP, Tremoli E, Zavan B, Ferroni L. Circulating Extracellular Vesicles in Cardiovascular Disease. Int J Mol Sci (2025) 26(14):6817. doi: 10.3390/ijms26146817

205. Qingpiao S, Yi Z. Exosomal miRNAs: a new frontier in cardiovascular disease diagnosis and treatment. J Cardiovasc Transl Res (2025) 18:1584--1592. doi: 10.1007/s12265-025-10617-y

206. Zhao X, Wang P. Interpretation of the molecular mechanism and therapeutic potential of microRNA-155 in cardiovascular and cerebrovascular diseases (review). Int J Mol Med (2026) 57:10. doi: 10.3892/ijmm.2025.5681

207. Huang X, Cheng J, Zhang L, Jia L, Wu C, Zheng L, et al. Comprehensive Immune-Phenotyping of Peripheral Lymphocytes in Coronary Artery Disease Reveals Potential Roles of Effector Memory and PD-1^+^ CD4^+^ T Cells. Inflammation (2026) 49(1):116. doi: 10.1007/s10753-026-02485-8

208. Tan J, Liang Y, Yang Z, He Q, Tong J, Deng Y, et al. Single-cell transcriptomics reveals crucial cell subsets and functional heterogeneity associated with carotid atherosclerosis and cerebrovascular events. Arterioscler Thromb Vasc Biol (2023) 43:2312--2332. doi: 10.1161/ATVBAHA.123.318974

209. Morgan H, Little K, Dutta S, Chen S, Gong J, Koduri S, et al. Cell-Free Nucleic Acids in Cardiovascular Disease: From Biomarkers to Mechanistic Drivers and Therapeutic Opportunities. Cells (2025) 15(1):33. doi: 10.3390/cells15010033

210. Artner T, Sharma S, Lang IM. Nucleic acid liquid biopsies in cardiovascular disease: cell-free DNA liquid biopsies in cardiovascular disease. Atherosclerosis (2024) 398:118583. doi: 10.1016/j.atherosclerosis.2024.118583

211. Charidemou E, Felekkis K, Papaneophytou C. From Natriuretic Peptides to microRNAs: Multi-Analyte Liquid Biopsy Horizons in Heart Failure. Biomolecules (2025) 15(8):1189. doi: 10.3390/biom15081189

212. Corpuz JCG. Circulating-free DNA and myocardial infarction: From diagnostic promise to clinical translation. Int J Cardiol Cardiovasc Risk Prev (2025) 27:200517. doi: 10.1016/j.ijcrp.2025.200517

213. Krittanawong C, Zhang H, Wang Z, Aydar M, Kitai T. Artificial intelligence in precision cardiovascular medicine. J Am Coll Cardiol (2017) 69:2657--2664. doi: 10.1016/j.jacc.2017.03.571

214. Anastasiou IA, Kounatidis D, Vallianou NG, Rebelos E, Karampela I, Dalamaga M. Clonal hematopoiesis of indeterminate potential and cardiometabolic disease: challenges, controversies and future perspectives. Int J Mol Sci (2025) 27:233. doi: 10.3390/ijms27010233

215. Luo Y, Zhang N, Yang J, Cui M, Tsoi KKF, Lip GYH, et al. AI-based multiomics profiling reveals complementary omics contributions to personalized prediction of cardiovascular disease. Nat Commun (2026) 17:2269. doi: 10.1038/s41467-026-68956-6].

The in-text citation number was incorrect in the main text.

A correction has been made to the section [1 Introduction, paragraph 3]:

“[Social stress causes vascular inflammation via neuro-endocrine-immune axis interactions, and environmental pollutants such as PM2.5 drive immune system sensitization and low-grade inflammation (6, 14, 15).]”

The in-text citation number was incorrect in the main text.

A correction has been made to the section [2 Fundamental Immune and Inflammatory Mechanisms in Cardiovascular Disease]:

“[Notably, emerging concepts such as trained immunity and innate immune memory, as well as age-related genetic immune changes like Clonal Hematopoiesis of Indeterminate Potential (CHIP), are critical to understanding the persistence and individual variability of CVD-related inflammation, and are integrated into the following discussion (18, 19, 90).]”

The in-text citation number was incorrect in the main text.

A correction has been made to the section [2.1 Synergistic Imbalance between Innate and Adaptive Immunity, paragraph 3]:

“[This persistent M1 polarization is further reinforced by innate immune memory: diverse innate signals such as free cholesterol or oxLDL induce low-grade inflammatory memory in innate immune cells not via signal type, but via sustained signal dosage and duration, leading to irreversible chronic inflammation

(21, 30). *In vivo* studies confirm this principle: mice with prolonged high-fat diet (HFD) feeding retain a low-grade inflammatory state even after HFD withdrawal, as innate immunity acts as a biological “timer”—sustained challenges trigger an irreversible low-grade inflammatory state that persists long-term (31, 32).]”

[An unnecessary in-text citation was included in the original text].

A correction has been made to the section [2.3 Immune-Metabolic Interactions and the Persistence of Inflammation, paragraph 9]:

“[Additionally, translational studies have identified novel therapeutic strategies for reversing innate immune memory: short chain fatty acid (SCFA) derivatives such as 4-PBA robustly rejuvenate innate monocytes and erase low-grade inflammatory memory, representing a promising translational approach for treating chronic CVD-related inflammation (79, 80).]”

The in-text citation number was incorrect in the main text.

A correction has been made to the section [2.4 Key Translational and Therapeutic Implications]:

“[1. Targeting innate immune memory/trained immunity: SCFA derivatives (4-PBA) to reverse inflammatory memory in innate monocytes; epigenetic modulators to target central/peripheral trained immunity (80, 81).

4. Cardio-oncology toxicity management: Targeting adaptive immune overactivation for ICI-related cardiovascular damage (85, 174)];”

[An unnecessary in-text citation was included in the original text].

A correction has been made to the section [3 Trained Immunity and Lifestyle Factors, paragraph 1]:

“[Diverse innate signals (e.g., free cholesterol, oxLDL) can induce low-grade inflammatory memory when sustained in the systemic environment for an extended period—this is distinct from the original trained immunity concept, where innate immune cells mount an amplified response after initial priming by specific ligands such as beta-glucan (21, 91).

In the CVD context, this “sustained signal-induced” innate immune memory is far more clinically relevant, as chronic low-level pro-inflammatory stimuli are ubiquitous in CVD pathogenesis. Additionally, trained immunity is now classified into central and peripheral subtypes based on its cellular origin: central trained immunity occurs in bone marrow stem cells, shaping the developmental trajectory of innate immune cells, while peripheral trained immunity is driven by epigenetic and metabolic reprogramming of tissue-resident macrophages (92, 93).]”

The original version of this article has been updated.

[The in-text citation number was incorrect in the main text].

A correction has been made to the section [3.1 Diet-Induced Trained Immunity and Chronic Inflammation, paragraph 2]:

“[*In vivo* studies have validated this memory principle: mice fed a prolonged high-fat diet retain a low-grade inflammatory state even after withdrawal of the high-fat diet, demonstrating that innate immunity acts as a biological “timer”—once pro-inflammatory challenges persist long enough, the system enters an irreversibly sustained low-grade inflammatory state (31, 32).]”

[The in-text citation number was incorrect in the main text].

A correction has been made to the section [3.1 Diet-Induced Trained Immunity and Chronic Inflammation, paragraph 5]:

“[Notably, SCFA derivatives such as 4-PBA have emerged as promising translational interventions: recent studies show 4-PBA can robustly rejuvenate innate monocytes and erase low-grade inflammatory memory in CVD-relevant models, representing a novel strategy to reverse sustained signal-induced trained immunity (80).]”

[The in-text citation number was incorrect in the main text].

A correction has been made to the section [5.1 Environmental Triggers of Cardiovascular-Immune Damage, paragraph 1]:

“[PM2.5 (fine particles of less than 2.5 m diameter) may contribute to higher CVD risks. It may penetrate into the respiratory tract, enter the blood, and enter different organs, including heart and blood vessels (163).]”

[The in-text citation number was incorrect in the main text].

A correction has been made to the section [5.2 Drug-Induced Cardiovascular-Immune Injury: Cardio-Oncology Focus, paragraph 2 and paragraph 3]:

“[Notably, ICIs and CAR-T therapy exhibit distinct mechanistic pathways of cardiovascular toxicity, a critical distinction for clinical management: ICIs targeting PD-1, PD-L1, or CTLA-4 cause cardiovascular toxicity via adaptive immune overactivation: they remove inhibitory signals that normally restrain T-cell responses, and this immune disinhibition can promote myocarditis, pericarditis, arrhythmias, and heart failure (174). ICI-associated myocarditis, though relatively uncommon, carries high mortality and is characterized by T-cell infiltration of the myocardium, elevated cardiac biomarkers (e.g., troponin), and increased inflammatory cytokines. Early recognition and prompt immunosuppressive therapy, typically with corticosteroids, are critical for management (85).

CAR-T therapy induces cardiovascular toxicity primarily through innate immune cell dysregulation, driving systemic inflammatory complications (85). Cytokine release syndrome (CRS), driven by massive cytokine production, can cause hypotension, endothelial dysfunction, capillary leakage, and thromboinflammation, ultimately leading to cardiac dysfunction or shock (85).]”

[The in-text citation number was incorrect in the main text].

A correction has been made to the section [7 Future Directions, paragraph 1, paragraph 2, paragraph 3 and paragraph 4]:

“[Emerging research has refined the trained immunity concept to emphasize dosage/duration-dependent innate immune memory and central/peripheral subtypes, but the clinical relevance of these subtypes in human CVD, as well as the reversibility of sustained signal-induced inflammatory memory, remains to be elucidated (92). And it remains unclear if trained immunity uniformly contributes to cardiovascular diseases or might exert context-dependent protective effects.The discovery of trained immunity has opened new avenues for investigating its causal role in human diseases, highlighting the need for further research beyond associative findings (90). Additionally, the translational potential of SCFA derivatives (e.g., 4-PBA) in erasing inflammatory innate immune memory requires further validation in human clinical trials (80).

Although NET formation has been found to have a negative influence on plaque and thrombosis, it is not certain whether NETs are causes or a primary factor in inflammation. Furthermore, if NET formation is inhibited pharmacologically, it could potentially impede host defense against infection and safety issues (51).

Allostatic load provides a framework for understanding the cumulative biological impact of chronic stress across multiple physiological systems, with ongoing research needed to establish standardized measurement approaches and validate its clinical utility in cardiovascular populations (152).

Multi-omics methods provide unprecedented information about immunometabolic networks, but whether the high dimensional data provide additional information than the traditional cardiovascular risk score is not clear. Cost and interpretability are also important constraints for clinical implementation (201).]”

[The in-text citation number was incorrect in the main text]. A correction has been made to the section [8 Conclusions]:

“[Age-related genetic immune changes (e.g., CHIP), innate immune memory shaped by the dosage and duration of pro-inflammatory signals, and central/peripheral trained immunity subtypes further expand our understanding of the heterogeneous pathogenesis of CVD (3, 21, 92).

Notably, SCFA derivatives such as 4-PBA show promise in rejuvenating innate monocytes and erasing inflammatory memory, representing a novel translational direction for reversing sustained signal-induced trained immunity (80).

The concept of allostatic load, first introduced by McEwen and elaborated by Juster et al., provides a framework for understanding how chronic stress becomes biologically embedded through the cumulative dysregulation of multiple physiological systems (152).

The recent discovery of distinct cardiovascular toxic mechanisms of ICIs (adaptive immune overactivation) and CAR-T therapy (innate immune dysregulation) refines cardio-oncology management (85).

The integration of CHIP screening and trained immunity characterization into precision immunocardiology frameworks is expected to shape cardiovascular prevention and therapy in the coming decade (2, 92).]”

The original version of this article has been updated.

